# Reduced endosomal microautophagy activity in aging associates with enhanced exocyst‐mediated protein secretion

**DOI:** 10.1111/acel.13713

**Published:** 2022-09-18

**Authors:** Gregory J. Krause, Antonio Diaz, Maryam Jafari, Rabia R. Khawaja, Esperanza Agullo‐Pascual, Olaya Santiago‐Fernández, Alicia L. Richards, Kuei‐Ho Chen, Phillip Dmitriev, Yan Sun, Stephanie K. See, Kotb Abdelmohsen, Krystyna Mazan‐Mamczarz, Nevan J. Krogan, Myriam Gorospe, Danielle L. Swaney, Simone Sidoli, Jose Javier Bravo‐Cordero, Martin Kampmann, Ana Maria Cuervo

**Affiliations:** ^1^ Department of Developmental and Molecular Biology Albert Einstein College of Medicine Bronx New York USA; ^2^ Institute for Aging Studies, Albert Einstein College of Medicine Bronx New York USA; ^3^ Microscopy and Advanced Bioimaging Core, Icahn School of Medicine at Mount Sinai New York New York USA; ^4^ Department of Cellular Molecular Pharmacology University of California San Francisco San Francisco California USA; ^5^ The J. David Gladstone Institutes San Francisco California USA; ^6^ Quantitative Biosciences Institute (QBI), University of California San Francisco San Francisco California USA; ^7^ Department of Biochemistry Albert Einstein College of Medicine Bronx New York USA; ^8^ Department of Biochemistry and Biophysics Institute for Neurodegenerative Diseases, University of California, San Francisco San Francisco California USA; ^9^ Laboratory of Genetics and Genomics, National Institute on Aging Intramural Research Program National Institutes of Health Baltimore Maryland USA; ^10^ Department of Medicine, Division of Hematology and Medical Oncology Icahn School of Medicine at Mount Sinai New York New York USA; ^11^ The Tisch Cancer Institute, Icahn School of Medicine at Mount Sinai New York New York USA

**Keywords:** aging, autophagy, chaperones, endosomal microautophagy, exocyst complex, late endosomes, protein secretion, proteostasis

## Abstract

Autophagy is essential for protein quality control and regulation of the functional proteome. Failure of autophagy pathways with age contributes to loss of proteostasis in aged organisms and accelerates the progression of age‐related diseases. In this work, we show that activity of endosomal microautophagy (eMI), a selective type of autophagy occurring in late endosomes, declines with age and identify the sub‐proteome affected by this loss of function. Proteomics of late endosomes from old mice revealed an aberrant glycation signature for Hsc70, the chaperone responsible for substrate targeting to eMI. Age‐related Hsc70 glycation reduces its stability in late endosomes by favoring its organization into high molecular weight protein complexes and promoting its internalization/degradation inside late endosomes. Reduction of eMI with age associates with an increase in protein secretion, as late endosomes can release protein‐loaded exosomes upon plasma membrane fusion. Our search for molecular mediators of the eMI/secretion switch identified the exocyst‐RalA complex, known for its role in exocytosis, as a novel physiological eMI inhibitor that interacts with Hsc70 and acts directly at the late endosome membrane. This inhibitory function along with the higher exocyst‐RalA complex levels detected in late endosomes from old mice could explain, at least in part, reduced eMI activity with age. Interaction of Hsc70 with components of the exocyst‐RalA complex places this chaperone in the switch from eMI to secretion. Reduced intracellular degradation in favor of extracellular release of undegraded material with age may be relevant to the spreading of proteotoxicity associated with aging and progression of proteinopathies.

## Graphical abstract

Endosomal‐microautophagy (eMI) malfunctions in aging due to altered dynamics and stability of the eMI machinery in late endosomes/multivesicular bodies (LE/MVBs). eMI failure associates with higher LE/MVB recruitment of RalA and organization of exocyst subunits into complexes that could mediate secretion of LE/MVB contents upon their fusion with the plasma membrane (PM). The exocyst complex acts as a molecular switch from protein degradation to secretion when eMI malfunctions in aging.

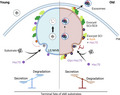

**Abbreviations**
CMA: chaperone‐mediated autophagy
CRISPRi: CRISPR interference
eMI: endosomal‐microautophagy
Endo H: Endoglycosidase H
ESCRT: endosomal sorting complexes required for transport
GAPDH: glyceraldehyde 3‐phosphate dehydrogenase
hsc: heat shock cognate protein
LAMP: lysosome‐associated membrane protein
LC3: light chain protein 3
LE/MVB: late endosome/multivesicular bodies
N/L: ammonium chloride and leupeptin
PNGase F: Peptide: N‐glycosidase F
sgRNA: short guide RNA
STED: Stimulated emission depletion

## 1 INTRODUCTION

Cells constantly renew their proteome to ensure proteostasis and to adjust protein levels to cellular needs. Proteostasis is accomplished through coordinated function of chaperones and proteolytic systems, which work together to identify and eliminate damaged proteins (Sala et al., 2017). Loss of proteostasis is a hallmark of aging and contributes to the pathogenesis of age‐related pathologies such as neurodegenerative diseases, metabolic conditions, or cancer (Kaushik & Cuervo, 2015; Lopez‐Otin et al., 2013). Autophagy, the degradation of intracellular contents in lysosomes or late endosome/multivesicular bodies (LE/MVBs), is an essential component of the proteostasis network. Several forms of autophagy co‐exist in most mammalian cells including macroautophagy, which mediates degradation of proteins and whole organelles entrapped in double membrane vesicles that then fuse with lysosomes (Galluzzi et al., 2017), and chaperone‐mediated autophagy (CMA), dedicated to selective degradation of individual cytosolic proteins that directly cross the lysosomal membrane (Kaushik & Cuervo, 2018). A third type of mammalian autophagy is endosomal microautophagy (eMI), which allows for the selective uptake of cytosolic proteins in LE/MVBs (Sahu et al., 2011). Like CMA, eMI is initiated by the recognition of a pentapeptide KFERQ‐like motif (Kirchner et al., 2019) in the amino acid sequence of substrate proteins by the heat shock cognate protein of 70 kDa (Hsc70) (Sahu et al., 2011). Hsc70 then binds to phosphatidylserine residues on the LE/MVB membrane (Morozova et al., 2016), and through interaction with the chaperone Bag6 (Krause et al., 2022) triggers internalization of the substrate protein via invaginations of the LE/MVB limiting membrane. These invaginations form in a manner dependent on the endosomal sorting complexes required for transport (ESCRT) (Sahu et al., 2011). In addition to protein internalization through eMI and other microautophagy‐related pathways (Schuck, 2020), the LE/MVB receives extracellular cargo through heterophagy pathways (pinocytosis, phagocytosis, endocytosis) (Johannes, 2021; Müller et al., 2015; Piper & Katzmann, 2007) and contributes to the turnover of ubiquitinated plasma membrane receptors via the ESCRT pathway (Shields & Piper, 2011), further emphasizing the importance of this compartment in protein degradation.

Because eMI was only recently discovered, its physiological relevance is still poorly understood. Activation of eMI as part of the starvation response in *Drosophila* has been described (Mukherjee et al., 2016). The effect of nutrient deprivation on eMI in mammalian cells is less clear, with upregulation of a variant of eMI very early in starvation (Mejlvang et al., 2018) and gradual decline in eMI activity as starvation is sustained (Krause et al., 2022). eMI contributes to clearance of prone‐to‐aggregate proteins such as Tau; however, pathogenic Tau variants found in Tauopathies fail to be degraded by eMI and instead hinder this type of autophagy by promoting docking of LE/MVBs in the plasma membrane and subsequent release of the pathogenic Tau variants (Caballero et al., 2018; Caballero et al., 2021). These findings suggest that eMI dysfunction could be involved in the pathogenesis and progression of Tauopathies through propagation of proteotoxicity. Extracellular release of undegraded eMI cargo may represent an alternative way for cells to rid themselves of toxic products, particularly when degradation is impaired, as is the case in aging (Buratta et al., 2020). LE/MVBs isolated from old mice have been found to contain high levels of oxidized proteins, suggesting an age‐related defect in the degradative capacity of LE/MVBs that could result in defective eMI in aging (Cannizzo et al., 2012). However, possible changes in eMI with age and the molecular defect(s) behind those changes have not been directly studied.

In this work, we demonstrate a decline in eMI activity with age and place it at the level of internalization and degradation of substrate proteins. We next identify changes with age in the stability and dynamics of LE/MVB‐associated Hsc70 that we attribute to age‐dependent glycation of this chaperone. In the second part of this study, we focus on the potential relationship between the eMI degradative function of LE/MVBs and their known role in exosome biogenesis and release (Hessvik & Llorente, 2018). We demonstrate an increase in protein secretion in aging that we attribute to reduced intracellular protein degradation and identify an additional previously unknown function of the exocyst complex and the GTPase RalA (Exocyst‐RalA complex), known for their involvement in vesicle secretion (Moskalenko et al., 2003; Wang et al., 2004), as negative regulators of eMI. Abnormally elevated levels of the exocyst/RalA complex in LE/MVBs with age provides a potential mechanism linking reduced eMI degradation and enhanced exocytosis of undegraded material in aging.

## 2 RESULTS

### 2.1 eMI activity decreases with age

**FIGURE 1:**
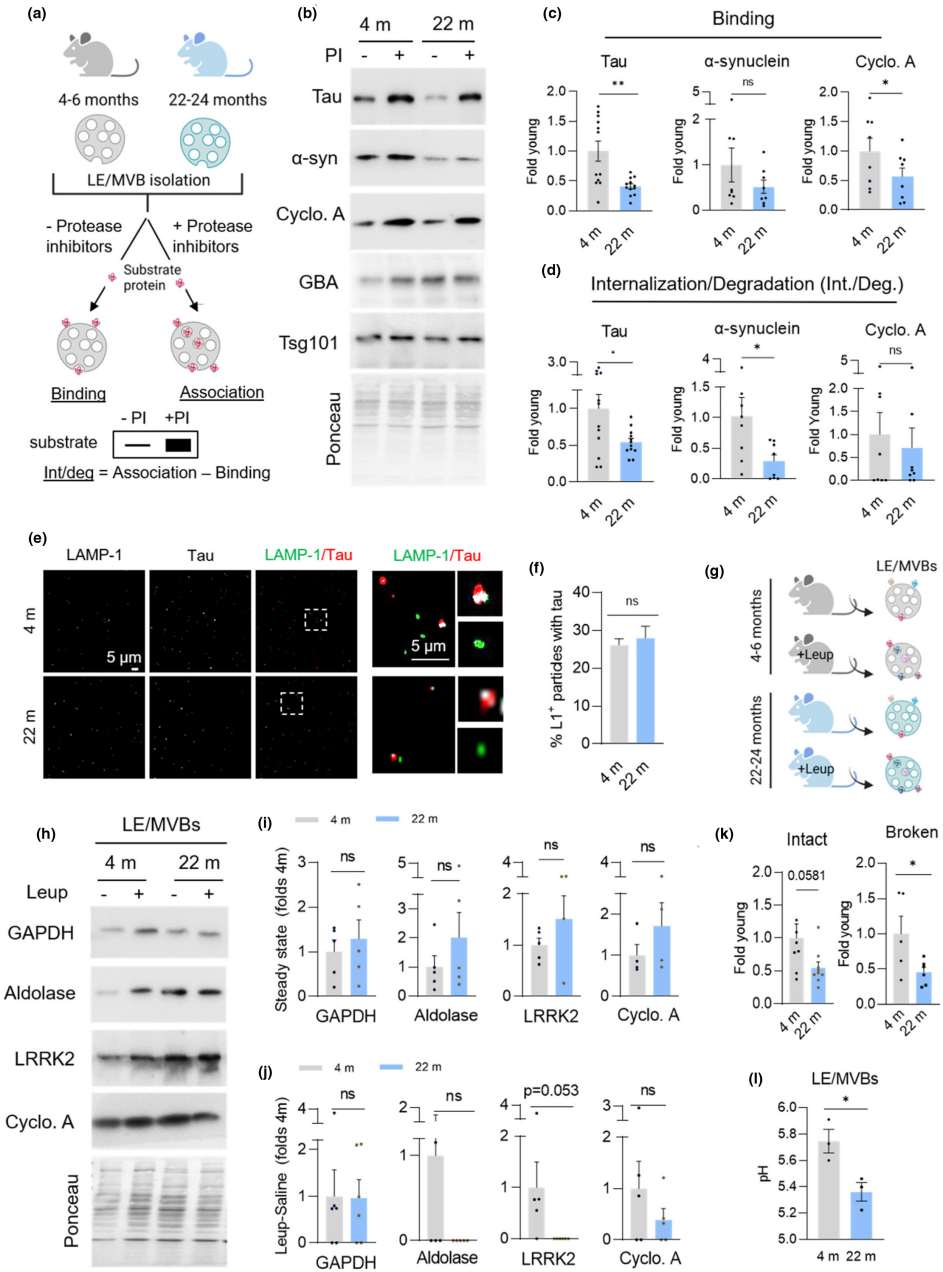
eMI activity decreases with age. (a–d) in vitro eMI reconstitution assay with isolated mouse liver LE/MVBs. Experimental design (a) and representative immunoblots (b) of LE/MVBs isolated from livers of 4 m and 22 m old mice incubated with (+) or without (−) protease inhibitors (PI) and the indicated substrate proteins. GBA and Tsg101 are shown as LE/MVB markers. Substrate binding (c) and internalization/degradation (int./deg.) (d) quantified in n = 12 (Tau) or 8 (Cyclophilin A, α‐synuclein) mice. (e,f) Representative confocal microscopy images (e) and quantification (f) from LE/MVBs isolated from 4 m and 22 m old mice immunostained for the indicated proteins following incubation with Tau (e). Inset: Higher magnification of the boxed area with colocalization mask (in white). N ≥ 15 fields from 6 (4 m) and 3 (22 m) mice. (g‐j) Experimental design (g), representative immunoblots (h) and quantification of steady‐state levels (i) and degradation (j) of the indicated endogenous eMI substrates in LE/MVBs isolated from 4 m and 22 m old mice injected without or with leupeptin to block endolysosomal degradation. Data is expressed relative to average value of saline injected 4 m old animals. N = 5 mice. (k) Proteolytic activity of intact (left) and detergent disrupted (broken, right) LE/MVBs from 4 m and 22 m old mice incubated with a pool of radiolabeled cytosolic proteins. N = 9 (intact), 6 (broken) mice. (l) pH measurement from isolated LE/MVBs of 4 m and 22 m old mice n = 3 mice. All data are mean ± SEM and individual values. Ponceau staining is shown as loading control in the immunoblots. Paired (c,d) and unpaired (rest) two‐tailed t‐test were used. Differences were significant for *p < 0.05 and **p < 0.01. ns, not significant

Previous data showed that LE/MVBs from old animals accumulate undegraded oxidized proteins (Cannizzo et al., 2012), suggesting a possible age‐related decline in eMI function. To investigate this possibility, we isolated LE/MVBs from young (4–6 m) and old (22–24 m) mouse livers and reconstituted the different steps of eMI in vitro using a previously developed assay (Krause & Cuervo, 2021; Sahu et al., 2011) (Figure 1a; comparable purity of fractions isolated from young and old mice was verified by immunoblot for LE/MVBs markers and enzymatic assays for the endolysosomal enzyme β‐hexosaminidase [Figure S1a–c]). LE/MVBs were incubated with two KFERQ‐motif containing proteins (Tau and α‐synuclein), previously shown to undergo degradation by selective eMI (Caballero et al., 2018; Krause et al., 2022) or with Cyclophilin A, which lacks a KFERQ motif and can be internalized and degraded in LE/MVBs through non‐selective eMI (Sahu et al., 2011) (Figure 1b). Incubation in the presence or absence of protease inhibitors allows for the differentiation of substrate binding (substrate recovered in LE/MVBs not treated with protease inhibitors) and substrate internalization/degradation (calculated as the difference between the substrate recovered in LE/MVBs pre‐treated with protease inhibitors and those untreated) (Figure 1a). We found reduced binding for the three proteins in LE/MVBs from old mice (Figure 1b,c), and significantly reduced internalization/degradation for both selective substrates, Tau and α‐synuclein, that was not observed for Cyclophilin A (Figure 1b,d), suggesting that the functional decrease with age preferentially affects KFERQ‐selective eMI.

Defective eMI with age could be a consequence of changes in activity per LE/MVB or in the fraction of cellular LE/MVBs that can perform eMI. To differentiate between these possibilities, we incubated LE/MVBs with the eMI substrate Tau and performed immunofluorescence of the glass‐spotted samples for Tau and the endolysosomal marker LAMP‐1 to measure the percentage of LE/MVBs competent for eMI (LAMP‐1^+^/Tau^+^) (Figure 1e). We observed no difference between the two ages in the percentage of LE/MVBs (LAMP‐1^+^ vesicles) that colocalized with the eMI substrate Tau (Figure 1f), suggesting that the fraction of LE/MVBs capable of performing eMI is preserved with age and that it is the absolute eMI activity per LE/MVB that decreases in the old group.

To determine if the reduction in eMI activity with age is observed in vivo and to explore possible changes in substrate targeting to LE/MVBs with age (a step not reconstituted in the in vitro system), we injected young and old mice with saline or the endolysosomal protease inhibitor leupeptin to prevent degradation in LE/MVBs (Figure 1g,h; efficacy of leupeptin injection was confirmed in total homogenate with proteins known to undergo lysosomal degradation [Figure S1d]). Immunoblot for three KFERQ‐bearing proteins (GAPDH, Aldolase and LRRK2) did not reveal reduction with age in the levels of endogenous eMI substrates associated with LE/MVBs (Figure 1i), suggesting that Hsc70‐mediated targeting to these organelles was comparable in young and old mice. However, once again, we found clear trends of reduced internalization/degradation for two of the selective endogenous substrates (Aldolase and LRRK2) (Figure 1j), which match their higher steady‐state levels in this compartment (Figure 1i) (likely bound to the membrane but not efficiently internalized/degraded). This decrease in eMI was not observed for one of the other endogenous substrates (GAPDH), suggesting possible substrate‐specific differences in their ability to undergo eMI with age and likely competition for LE/MVB internalization/degradation based on the cellular abundance of these proteins.

We next used a modified in vitro assay to analyze substrate internalization separately from degradation by measuring the proteolysis of a pool of radiolabeled proteins incubated with isolated LE/MVBs with intact membranes or upon disruption of their membranes with detergent to allow direct access of the LE/MVB enzymes to the radiolabeled substrates, and thus, eliminate the internalization step. We found a marked reduction in the degradation of radiolabeled proteins in both cases (Figure 1k), suggesting that at least part of the age‐related reduction in eMI is due to less efficient proteolysis by luminal enzymes.

Despite lower proteolytic activity, immunoblot of isolated LE/MVBs from old mice did not reveal major changes with age in levels of the luminal enzymes GBA and Cathepsin D (Figure S1a,b). We next considered the possibility of defective acidification. However, measurement of the pH of isolated LE/MVBs using the radiometric pH probe LysoSensor Yellow/Blue DND‐160 revealed higher acidification in old mice LE/MVBs (pH shift from 5.75 to 5.36) (Figure 1l). Direct measurement of the activity of other pH‐dependent enzymes in LE/MVBs such as β‐hexosaminidase, which hydrolyzes amino sugars, also confirmed higher activity in LE/MVBs from old mice (Figure S1c), further supporting their proper acidification.

Overall, these findings unveil a decline in selective eMI with age mostly due to defective degradation of internalized cargo. Reduced substrate degradation does not seem to be a consequence of overall changes in LE/MVB properties such as acidification but appears to be instead limited to defective protein break down.

### 2.2 LE/MVB‐associated Hsc70 is glycated with age

**FIGURE 2:**
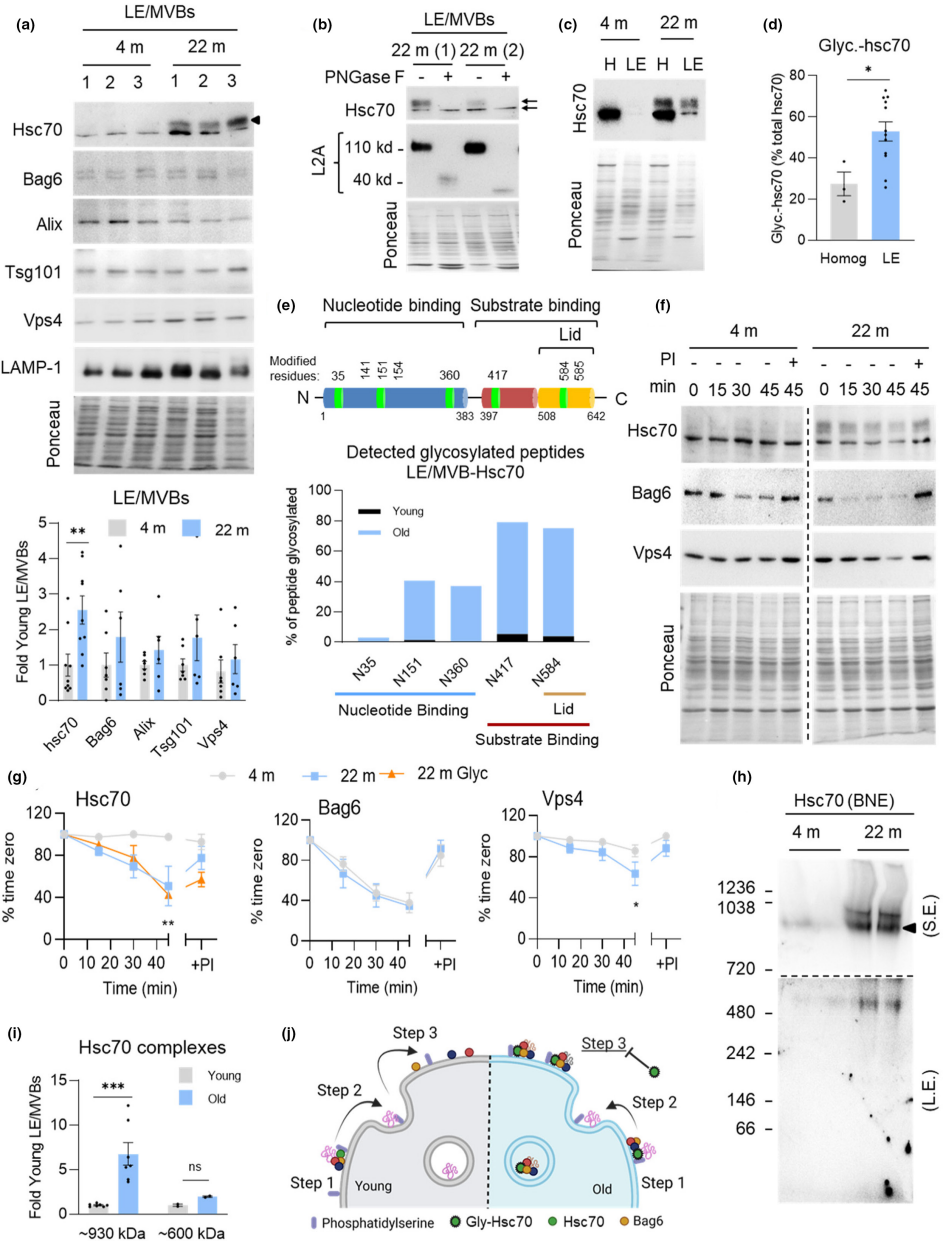
Altered properties of LE/MVB‐Hsc70 in aging. (a) Immunoblot (top) and quantification (bottom) for indicated proteins in LE/MVBs isolated from 4 m and 22 m old mouse livers. Black arrowhead indicates band unique to 22 m old group. N = 6–9 mice. (b) Treatments of LE/MVBs from 22 m old mice with the endoglycosidase PNGase F. LAMP‐2 (L2A) is shown as a control of a known glycosylated protein. Black arrows indicate distinct Hsc70 bands. N = 4 mice. (c,d) Immunoblot (c) and quantification (d) of glycosylated Hsc70 in homogenate (homog) and LE/MVB (LE) fractions from 4 m and 22 m old mouse livers. N = 3 (homog) or 12 (LE) mice. (e) Schematic of Hsc70 (top) with residues affected by age‐related glycosylation in green and comparison of degree of peptide glycosylation between Hsc70 associated with LE/MVBs from young and old mice (bottom). (f,g) Stability of eMI component proteins in LE/MVBs. Representative immunoblots (f) and percentage of protein remaining at the indicated times (g) in LE/MVBs from 4 m and 22 m old livers incubated at 37°C with (+) or without (−) protease inhibitors (PI). N = 4 independent experiments. Vertical dotted black lines indicate where membrane was cut to eliminate non‐relevant bands. (h,i) Immunoblot for Hsc70 after blue‐native electrophoresis of LE/MVBs from 4 m and 22 m old mice (h) and quantification of Hsc70‐containing complex abundance (i). Image of the top half of the membrane after short exposure (S.E.) (top) and the bottom half of the membrane after long exposure (L.E.) (bottom) is shown in h. Black discontinous line indicates the different exposure regions. N = 7. (j) Proposed model of the effect of Hsc70 changes with age on eMI activity. Data are mean ± SEM and individual values. Ponceau staining is shown as loading control in the immunoblots. One sample multiple t‐tests (a,i), unpaired two‐sided t‐test (d), and two‐way ANOVA with Bonferroni multiple comparisons post hoc test (g) were used. Differences were significant for *p < 0.05, **p < 0.01, ***p < 0.001, ns, not significant

We next sought to understand the molecular defects behind the functional decline in eMI by analyzing possible changes in the levels of eMI‐related components with age. Immunoblot analysis revealed that total cellular levels (Figure S2a) and LE/MVB levels (Figure 2a) of eMI components were comparable in both ages, with the exception of Hsc70, which is increased 2.5‐folds in LE/MVBs from old mice. The elevated levels of Hsc70 in old LE/MVBs was driven by an additional higher molecular weight band immunoreactive for Hsc70 only in LE/MVBs from old mice (Figure 2a). Treatment of LE/MVBs from old mice with different endoglycosidases to remove sugar moieties led to a discrete decrease in the molecular weight of the upshifted Hsc70 band after treatment with Endoglycosidase H (Endo H), which deglycosylates mannose glycoproteins (Figure S2b; LAMP‐1 is shown as positive control of glycosylated protein); whereas incubation with PNGase F, which removes all types of asparagine‐linked (N‐linked) glycosylation and advanced glycosylation end‐products from glycation, eliminated the high molecular weight form of Hsc70 (Figure 2b; LAMP‐2A is shown as positive control of glycosylated protein), consistent with an age‐related sugar modification as previously detected for other proteins in LE/MVBs from old organisms (Cannizzo et al., 2012). While this modification is detectable in liver homogenates from old mice (27% of the total cellular Hsc70), it is highly enriched in LE/MVBs where it represents 53% of LE/MVB‐associated Hsc70 (Figure 2c,d). It is unlikely that the glycosylated form of hsc70 in LE/MVBs is a cytosolic contaminant, since only after long exposure a small fraction of cytosolic hsc70 (<10%) displayed the molecular weight shift (Figure S2c). Mass spectrometry analysis identified that Hsc70 in LE/MVBs from old mice had sugar moieties in five asparagine residues not present or barely detectable in Hsc70 from young LE/MVBs (Figure 2e). The modified residues were distributed (i) along the nucleotide binding region (with N^35^ located in the protein/protein interface of the interaction of Hsc70 with co‐chaperones of the Bag family and N^151^ in close proximity to the nucleotide binding pocket), (ii) in the substrate binding region, and (iii) in the lid domain (with N^584^ adjacent to K^583^, one of the lysine residues known to mediate interaction of Hsc70 with phosphatidylserine on the LE/MVB membrane during eMI (Morozova et al., 2016)) (Figure S2d). We propose that glycation of these residues may affect the ability of Hsc70 to hydrolyze ATP, required for releasing bound substrates, its interaction with the co‐chaperone Bag6, recently shown to co‐operate with Hsc70 in the substrate internalization step of eMI (Krause et al., 2022), or interfere with the docking of Hsc70 at the LE/MVB (Morozova et al., 2016).

Since glycation can have a toxic effect on protein function (Simm, 2013), we analyzed changes in the behavior of Hsc70 in the LE/MVB compartment. To investigate the topology of glycated Hsc70, we incubated isolated LE/MVBs at room temperature with increasing concentrations of trypsin, which only degrades surface proteins (mTOR shown as control in Figure S2e). A larger fraction of the glycated form of Hsc70 was still present after trypsinization, suggesting that it may be more readily internalized into LE/MVBs than unmodified Hsc70 (Figure S2e). Although persistence of glycated Hsc70 upon trypsinization could reflect intrinsic resistance to degradation by the protease, we discarded this possibility because we found that both unmodified and glycated Hsc70 displayed comparable kinetics of degradation in LE/MVBs. Incubation of LE/MVBs at physiological temperature (37°C) revealed a sharp reduction in levels of both the glycated and unmodified form of Hsc70 in old LE/MVB that could be prevented by inhibition of proteolysis, in support of the proposed higher internalization and degradation of both forms of Hsc70 with age (Figure 2f,g). In fact, Hsc70 behavior in old LE/MVBs resembled that of the co‐chaperone Bag6 (Figure 2f,g), previously shown to be internalized, with a fraction of it degraded along with internalized substrates as part of the eMI process (Krause et al., 2022). Stability of another eMI component, the ESCRT protein Vps4, was also reduced in old LE/MVBs, albeit to a lower extent (Figure 2f,g). Whether Vps4 instability is related to the change in Hsc70 with age or is an independent event will require further investigation.

To further investigate possible aberrant protein–protein interactions and/or overall changes in the organization of Hsc70 in LE/MVBs with age, we performed blue‐native electrophoresis of isolated organelles. We detected a high molecular weight protein complex (~970 kDa) immunoreactive for Hsc70 only present in old LE/MVBs (Figure 2h), and two additional Hsc70 complexes (~930 and 600 kDa), also detected in young LE/MVBs but in significantly lower abundance than in old LE/MVBs (Figure 2h,i). The increase in abundance of Hsc70 in these high molecular weight complexes in old LE/MVB was almost 3 times the increase in overall levels of Hsc70 in these organelles (Figure 2a), supporting that at any given time, a larger fraction of Hsc70 in old LE/MVBs is organized in protein complexes in these organelles. We favor a model where instead of the typical transient interactions of Hsc70 with substrates and other eMI components in LE/MVBs, glycated forms of Hsc70 establish more stable interactions and consequently limit the number of sites and/or components available for eMI activity and slows down this process (Figure 2j).

**FIGURE 3:**
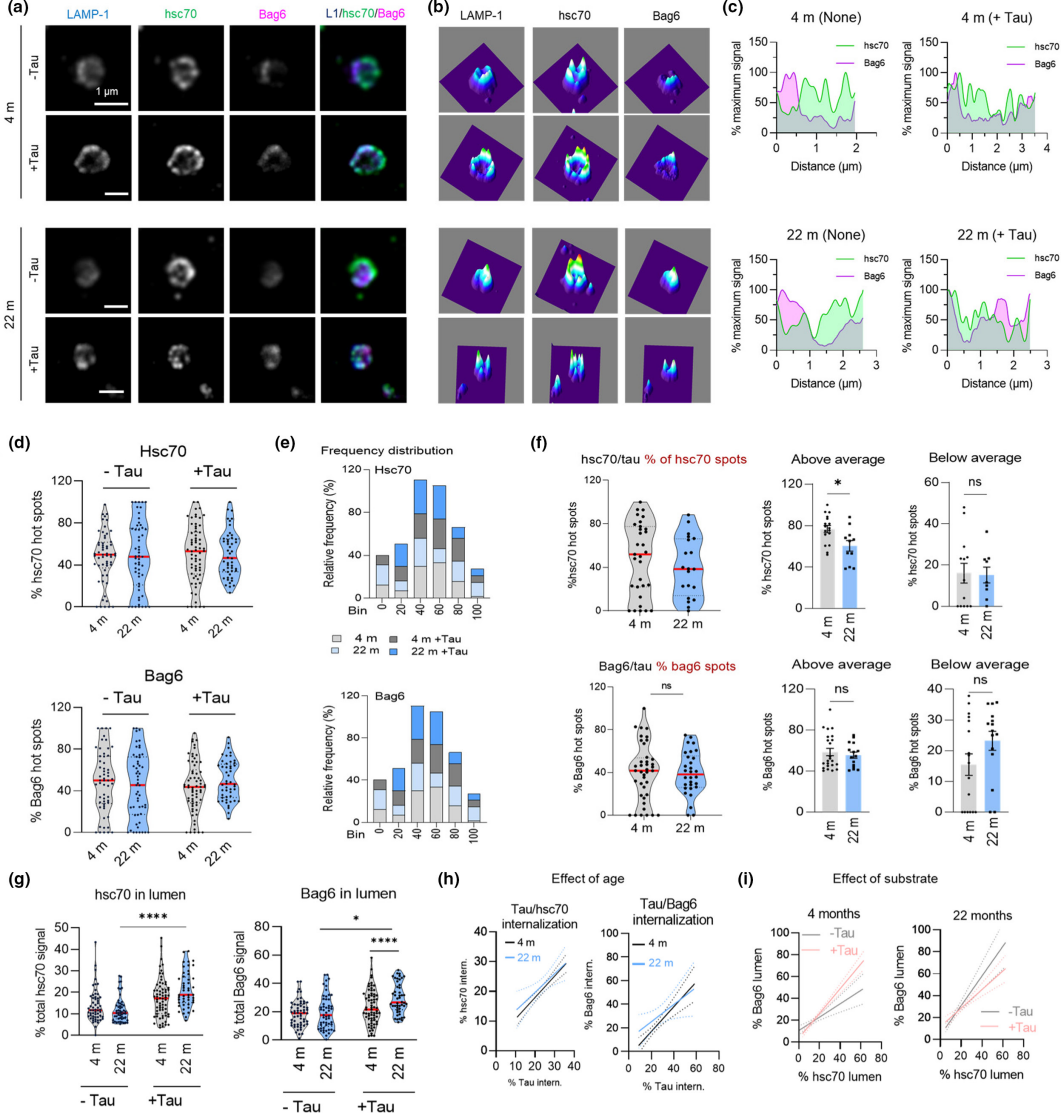
Age‐related changes in the LE/MVB dynamics of Hsc70 and Bag6. (a–c) Representative τ‐STED microscopy images of isolated LE/MVBs from 4 m (as previously reported (Krause et al., 2022)) and 22 m old animals incubated with (+) or without (−) the eMI substrate Tau and immunostained for LAMP‐1, Hsc70, and Bag6. Single channel black and white original images (a, left) and pseudocolored 3 channels merged image (a, right), 2.5‐D density plots (b) and fluorophore images (a), 2.5‐D density plots (b) and fluorophore intensity plotting along the LE/MVB membrane (c) are shown. (d,e) Percentage of protein (d) and relative frequency (e) of Hsc70 (top) and Bag6 (bottom) in hot spots in the membrane of same LE/MVBs as in (a). Red lines in (d) indicate the average for each condition. N = 5 animal, ≥45 LE/MVBs per condition. (f) Overlap of hot spots of Tau and Hsc70 (top) and Tau and Bag6 (bottom) with respect to the total number of hot spots for each eMI chaperone. Violin plots of the percentage of each chaperone in hot spots (left) and percentage of LE/MVBs that are above (middle) or below (right) the average for each age group. Red lines indicate the average for each condition. N = 3 animals, ≥20 LE/MVBs per condition. (g) Quantification of the percentage of internalized Hsc70 (left) and Bag6 (right) in the LE/MVB lumen of each condition. Red lines indicate the average for each condition. N = 5 animal, ≥45 LE/MVBs per condition. (h,i) Effect of age (h) and incubation with the substrate (i) on the fraction of Hsc70 and Bag6 internalized relative to the fraction of Tau internalized (h) or to each other (i) in LE/MVBs isolated from 4 m and 22 m old animals. N = 3 animals, ≥20 LE/MVBs per condition (h) and n = 5 animal, ≥45 LE/MVBs per condition (i). Data are mean ± SEM and individual values. Two‐way ANOVA with Bonferroni's multiple comparisons post hoc test (d,g), unpaired two‐sided t‐test (f), and simple linear correlation (h,i) were used. Differences were significant for *p < 0.05, ***p < 0.001. ns, not significant

We next used stimulated emission‐depletion (τ‐STED) microscopy, a variety of super‐resolution microscopy, in isolated LE/MVBs to investigate changes with age in the organization and dynamics of Hsc70 and Bag6. After incubation or not of isolated LE/MVBs with Tau protein to stimulate eMI activity, we immunostained for LAMP‐1 to label the LE/MVB membrane, Tau, and the two chaperones (Figure 3a,b). Quantitative tracing of the proteins between the external and internal perimeters of individual LE/MVBs revealed a relatively uniform distribution of Hsc70 along the membrane and areas of discrete Bag6 staining previously described as points of active eMI substrate internalization (Krause et al., 2022) (Figure 3c). Analysis of areas with high signal intensity, or “hot spots” (pixels with intensity >1.25 times the average pixel intensity) showed differences in the overall distribution of Hsc70 and of Bag6 in hot spots in the oldest group, mostly upon addition of the substrate protein (Figure 3d). Although the average distribution of both chaperones did not change significantly upon addition of Tau, we found differences with age in the fraction of LE/MVBs with chaperones preferentially inside or outside the “hot spot” regions. Young mice presented a relatively balanced fraction of LE/MVBs displaying the chaperones outside and inside hot spots, likely representative of substrate binding and internalization sites, respectively (Figure 3e). In contrast, in most LE/MVBs from old mice, both proteins remained in areas of high signal intensity (Figure 3e), compatible with the higher Hsc70 clustering observed using biochemical procedures (Figure 2h). Analysis of the distribution of the chaperones relative to the substrate also revealed lower coincidence of substrate and chaperone in the Hsc70 “hot spots” (Figure 3f) in agreement with our hypothesis that part of the clustered modified Hsc70 was no longer capable of engaging in substrate binding/internalization.

We used the same tracing strategy to identify the fraction of each chaperone found in the lumen of LE/MVBs and the impact of activating eMI (by incubation with Tau) on their internalization. We noticed significantly higher levels of luminal Hsc70 in old LE/MVBs upon addition of Tau (11.9% vs. 21.2%) and a similar pattern for Bag6 (19.8% vs. 29.5%) (Figure 3g). Although a fraction of Hsc70 was also internalized in a substrate‐dependent manner in young LE/MVBs, Hsc70 internalized in the old LE/MVBs was 3‐fold more (78% in 22 m old vs. 26% in 4 m old), thus mirroring our biochemical findings (Figure 3f,g). Using simple linear correlation between Tau internalization and Hsc70 and Bag6 internalization (Figure 3h), we found that the strong correlation between substrate and chaperone internalization noted in young LE/MVBs (slope = 1.052, R^2^ = 0.41, *p* < 0.0001) was less evident in old LE/MVBs, where higher amounts of chaperones were internalized per molecule of substrate (slope = 0.7303, R^2^ = 0.14, *p* = 0.037). It is possible that chaperone internalization with age occurs in part in a substrate‐independent manner and/or that the observed clustering of Hsc70 in the old LE/MVBs promotes internalization of multi‐chaperone complexes per substrate in these conditions. In fact, we also noticed some level of dissociation with age in the LE/MVB dynamics of Bag6 relative to Hsc70. We recently found that the ratio of Bag6 internalized for every Hsc70 molecule changes with eMI activity (Krause et al., 2022), with a higher amount of Bag6 to Hsc70 internalized when eMI is activated, as shown here for young LE/MVBs (Figure 3i). However, similar analysis in the old LE/MVBs revealed a loss of the coordinated substrate‐dependent increase in Bag6 internalization with Hsc70 (Figure 3i), in support of altered Bag6/Hsc70 dynamics.

Overall, our findings suggest that reduced eMI with age may be a consequence of altered Hsc70 dynamics at the LE/MVB membrane that lead to its higher internalization and subsequent augmented degradation in this compartment. We attribute the disrupted dynamics to age‐related post‐translational modifications on Hsc70, such as the glycation identified here, that affect the normal interaction of this chaperone with substrates and other eMI components.

### 2.3 Impact of reduced eMI activity with age on the intracellular proteome

Since our analysis of endogenous eMI substrates indicated that the impact of aging on eMI may be, to some extent, substrate‐dependent (Figure 1i,j), we performed comparative quantitative proteomics of isolated LE/MVBs from young and old mice to determine changes in the profile of eMI substrates with age. We injected half of the mice with leupeptin, as in Figure 1g, to inhibit proteolysis in the LE/MVB lumen and thus cause the accumulation of endogenous substrates (proteins with an increase of ≥20% upon leupeptin injection) (Schneider et al., 2014). When proteolysis was not inhibited, more proteins displayed higher levels in LE/MVBs from old mice when compared to young mice (Figure S3a; 29% of the LE/MVB proteome in 22 m old vs. 15% in 4 m old). However, upon leupeptin administration, we detected a clear shift toward accumulation of more proteins in LE/MVBs from young mice and a marked reduction in the number of proteins undergoing degradation in the old (Figure S3b; 39% of the LE/MVB proteome in 4 m old vs. 11% in 22 m old). The more pronounced effect of the proteolysis blockage in young LE/MVBs is in agreement with the observed reduced proteolytic activity of old LE/MVBs (Figure 1k). In fact, under our experimental conditions, we found that 50% of proteins detected in young LE/MVBs were undergoing degradation compared with only 17% of proteins in old LE/MVBs (Figure S3b).

To differentiate substrate proteins reaching LE/MVBs through eMI (from the cytosol), from those degraded in LE/MVBs through heterophagy (pinocytosis, phagocytosis, endocytosis), we used as before (Krause et al., 2022) Gene Ontology (GO) analysis to eliminate proteins associated with the terms “extracellular space” and “plasma membranes”, which included 34% and 25% of the degraded proteins in young and old LE/MVBs, respectively. Analysis of the remaining proteins revealed 813 proteins undergoing degradation in LE/MVBs in young mice and only 334 in old mice (Figure S3c–f). Interestingly, in addition to the 676 proteins no longer degraded in the old LE/MVBs, there was a subset of proteins (197) not previously detected to undergo degradation in this compartment in young mice that were degraded in old mice (Figure S3f). This group included proteins normally present in LE/MVBs as constitutive components that become unstable in this compartment with age (184 proteins), as well as 13 cellular proteins only routed to LE/MVBs for degradation in the old group (Figure S3g). We also identified proteins with defective eMI targeting to LE/MVBs with age as those degraded in young LE/MVBs and no longer detected in the LE/MVBs from old mice injected or not with leupeptin (Figure S3g). Only 7 proteins fulfilled these criteria, further confirming that LE/MVB targeting of eMI substrates is preserved with age and that the primary defect in this autophagic pathway is in internalization/degradation of cargo.

Sequence analysis of proteins degraded in LE/MVBs confirmed similar enrichment in both age groups (~80%) of proteins bearing KFERQ‐like targeting motifs (indicative of selective eMI) (Kirchner et al., 2019) (Figure S3h). These proteins also showed comparable distribution of motifs constitutively present in the protein sequence (canonical) and those that become motifs upon post‐translational modifications, namely phosphorylation or acetylation (Figure S3h). These findings further support that most of the identified cargo reached LE/MVBs in all cases through KFERQ‐selective eMI. To determine the contribution and possible changes with age on the degradation of supersaturated proteins (proteins at risk of aggregation when their cellular concentrations reach their solubility limit), we analyzed the supersaturation scores (Ciryam et al., 2013) of proteins differentially degraded in LE/MVBs with age. We found similar scores for proteins degraded only in young or only in old mice when in their folded state (Figure S3i, left), but we noticed a significantly reduced ability of LE/MVBs from old mice to handle proteins with higher supersaturation scores in the unfolded state (Figure S3i, right). These findings are compatible with the failure of eMI with age contributing to the accumulation of prone‐to‐aggregate proteins often found in old organisms.

To gain information on the possible consequences of the quantitative and qualitative changes with age in the profile of proteins degraded by eMI, we investigated the cellular pathways in which those proteins participate. STRING analysis (Szklarczyk et al., 2019) (Figure S4a,b) revealed that degradation by eMI of proteins involved in other proteolytic systems (proteasome and mitophagy) was lost with age, whereas two major functional groups, ribosome and metabolism, were still enriched as eMI substrates in both ages. However, the fact that the specific proteins in those functional groups were different suggests that eMI may regulate similar pathways in aging, although via the degradation of different substrates, and consequently with different outcomes. To further refine the metabolic processes regulated by eMI in both ages, we used Reactome analysis (Jassal et al., 2020) (Figure S4c–f) and found that eMI predominantly degrades proteins that regulate metabolism of carbohydrates in young mice and those involved in metabolism of lipids, specifically fatty acids, in old mice (Figure S4e,f).

### 2.4 Protein secretion is elevated with age

**FIGURE 4:**
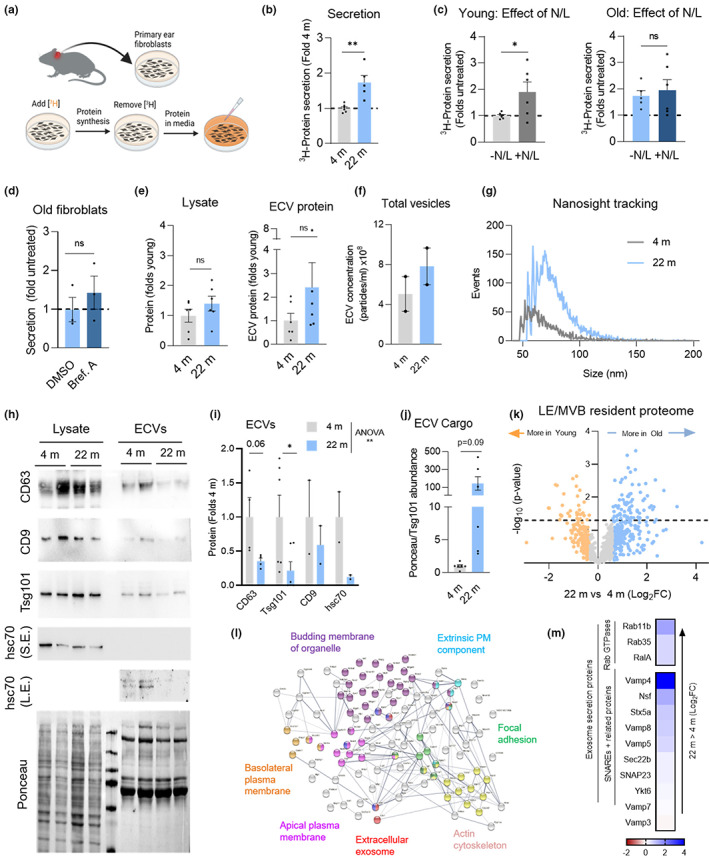
Protein secretion though exocytosis increases with age. (a–d) Measurement of radiolabeled protein secretion in mouse primary ear fibroblasts from 4 m and 22 m old mice. Experimental design (a), basal protein secretion (b), effect of acute inhibition of endolysosomal proteolysis in 4 m (c; left) and 22 m (c; right) old fibroblasts using a combination of ammonium chloride and leupeptin treatment (N/L) and effect of blocking protein secretion through conventional secretion (Brefeldin A) in 22 m old fibroblasts (d). N = 6 (b, c) and n = 3 (d) independent experiments (ie). (e–j) Extracellular vesicle (ECV) isolation from culture media of 4 m and 22 m old fibroblasts. Recovered protein in cell lysates (e; left) and ECV fraction (e; right). (f,g) nFCM NanoAnalyzer tracking analysis of number of total ECV per ml (f) and representative histogram of the size distribution of ECVs (g) isolated from culture media of 4 m and 22 m old fibroblasts. N = 2. (h–j) Representative immunoblots (h) and quantification of exosome marker proteins (CD63, Tsg101, and CD9) and the eMI chaperone Hsc70 (i), and of total protein content in ECV after normalizing to Tsg101 abundance in the ECV fraction in (j). N = 2–6. Ponceau staining is shown as loading control in (h). (k–m) Comparative proteomics LE/MVBs from livers of 4 m and 22 m old mice, after removing proteins undergoing flux. Log_2_ fold change (Log_2_FC) in protein abundance between (k), STRING analysis (l) and heat map of relative abundance (m) of resident proteins found exclusively in old LE/MVBs. All GO terms statistically enriched with *p* < 0.05. Data are mean ± SEM and individual values. Unpaired two‐sided (b–f,j) and one sample multiple t‐tests (i) were used. Differences were significant for *p < 0.05, **p < 0.01, L.E., long exposure; ns, not significant; S.E., short exposure

As the LE/MVB is also the site of exosome biogenesis (Stoorvogel et al., 2002), one of the potential consequences of reduced degradation of cargo internalized through eMI in aging could be increased exosome secretion as a way to remove toxic protein products from the cell. In fact, previous studies from our group have shown that this is the case for pathogenic forms of Tau proteins, which have inhibitory effects on different autophagic pathways. These proteins reach the extracellular space in large part through their eMI‐dependent delivery to LE/MVBs and subsequent fusion of these compartments with the plasma membrane (Caballero et al., 2021). To study the relationship between the impairment of intracellular proteolysis and changes in protein secretion with age, we used primary ear fibroblasts from young and old mice. To avoid the confounding effect of elevated secretion in cellular senescence (Coppé et al., 2008), we only used early passage cultures, which we previously confirmed contained less than 1% of senescent cells (positive for β‐galactosidase) (Bejarano et al., 2018). We used a pulse of metabolic labeling with ^3^H‐leucine and then measured secretion of radiolabeled proteins in the extracellular media of cultured fibroblasts (Figure 4a). We found a significant 73% increase in protein secretion by fibroblasts from old mice compared with young mice (Figure 4b). Interestingly, this increase in secretion can be reproduced in young mouse fibroblasts upon chemical blockage of their endolysosomal degradation with a combination of ammonium chloride and leupeptin (N/L) (Figure 4c, left). The lack of further increase over the already augmented protein secretion in old mouse fibroblasts upon treatment with endolysosomal proteolysis inhibitors (Figure 4c, right) further reinforces that faulty degradation in this compartment may be behind increased protein secretion.

Higher protein secretion in the old fibroblasts was not through conventional secretion because it remained unchanged upon treatment with Brefeldin A (conventional secretion inhibitor) (Ripley et al., 1993) (Figure 4d). In contrast, and in support of the contribution of unconventional secretion mechanisms, we found a markedly higher amount of protein (79% increase) present in extracellular vesicles (ECVs) isolated from the culture media of 22 m old mouse fibroblasts when compared with those from 4 m old mice (Figure 4e). This increase did not result from an increase in total intracellular protein content, which remained unchanged with age (Figure 4e).

nFCM NanoAnalyzer tracking analysis of vesicle size distribution revealed a 50% increase in the number of vesicles obtained in the old ECV fraction compared with the young ECV fraction (Figure 4f), and confirmed a predominant content of vesicles in the 30‐100 nm diameter range, compatible with exosomes (Figure 4g). Despite a higher number of vesicles, immunoblotting of the same amount of total ECV protein showed lower abundance of exosome markers CD63 and Tsg101 as well as of Hsc70 in ECVs from old fibroblasts (Figure 4h,i). We postulated that this reduction may reflect the accumulation of undegraded cargo in the ECVs of the old fibroblasts, which would lower the contribution of integral exosome markers to the total amount of loaded proteins. In fact, normalization of the protein loaded to the amount of the ECV marker Tsg101 confirmed a marked increase in the ratio of cargo to this exosome constituent protein (Figure 4j).

We next used the proteomic analysis of LE/MVBs from young and old mice livers (Figures S3,S4) to identify age‐related changes in the constitutive components of this compartment that could support a higher secretory propensity with age. We first compared the proteomic profile of LE/MVBs intrinsic proteins (those proteins detected in young LE/MVBs whose levels did not change upon inhibition of proteolysis). In the case of old mice LE/MVBs, we eliminated from this list those proteins identified as bona fide substrates in young LE/MVBs, as those represent cargo with compromised degradation with age. This comparison identified changes in 109 of the LE/MVBs resident proteins, with 33 proteins displaying reduced abundance and 76 present at higher levels in old mouse LE/MVBs (Figure 4k). Analysis of LE/MVB resident cathepsins and other hydrolases and of LE/MVB membrane proteins, including subunits of the vacuolar ATPase (V‐ATPase), showed no reduction in their levels with age, and in fact, most of them were more abundant in LE/MVBs from old animals (Figure S3j) in agreement with our immunoblot and enzymatic analysis (Figure S1a–c) and with the higher acidification observed in old LE/MVBs (Figure 1l). When considering all LE/MVB resident proteins, STRING analysis demonstrated enrichment in old LE/MVBs of proteins in functional families related with vesicular trafficking, membrane budding and association to the plasma membrane (Figure 4l), all events that could contribute to LE/MVB‐mediated protein secretion. Examples of proteins related with exosome‐mediated protein secretion that display higher levels in old mouse LE/MVBs are shown in Figure 4m.

### 2.5 The exocyst complex is a negative regulator of eMI

To identify possible molecular mediators of the proposed coordinated functioning of eMI and exocytosis, we performed a CRISPR interference (CRISPRi) screen in cells expressing a nuclease‐dead version of the Cas9 enzyme (dCas9) tagged with the KRAB transcriptional repressor and transduced for stable expression of the KFERQ‐split Venus eMI reporter (Figure S5a). This reporter allows visualization of the sequestration of this artificial eMI substrate in the inward budding vesicles at the LE/MVB limiting membrane (Caballero et al., 2018; Krause & Cuervo, 2021). The reporter only fluoresces when the two halves of the Venus protein come in close proximity in the confined space of the intraluminal vesicles of LE/MVBs, making measurement of the number of fluorescent puncta per cell a reliable measurement of eMI. We used a library of short guide RNAs (sgRNAs) targeting proteostasis‐related genes (including genes involved in protein folding, degradation and secretion and in endolysosomal and autophagy pathways [Horlbeck et al., 2016]) and used fluorescent activated cell sorting to separate cells with high (top 33%) and low (bottom 33%) Split Venus signal to determine the frequency of individual sgRNAs in each population by next‐generation sequencing (Figure S5b).

**FIGURE 5:**
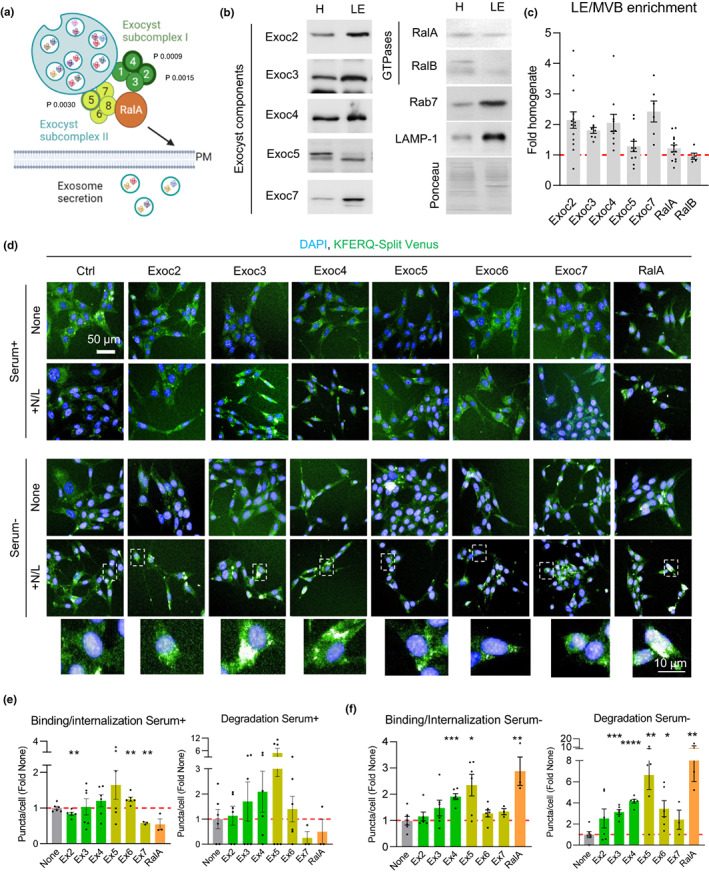
The exocyst complex inhibits eMI activity. (a–c) Presence of exocyst complex proteins on LE/MVBs. (a) Diagram of exocyst subcomplexes and GTPase RalA interaction. Subunits identified as hits with effect on eMI through the CRISPRi screen are marked with a thicker border and *p*‐values from the screen are indicated. Representative immunoblots of rat liver homogenates (H) and LE/MVBs (LE) (b) and quantification of LE/MVB enrichment for the indicated proteins (c). Ponceau staining is shown as loading control. N = 5–14 mice. (d–f) Representative images (d) of NIH3T3 mouse fibroblasts stably expressing the KFERQ‐split Venus reporter control (ctrl) or singly knocked‐down for the indicated components of the exocyst complex or RalA. Cells were untreated (none) or treated with ammonium chloride and leupeptin (+N/L) to block endolysosomal proteolysis and maintained in the presence (serum+) or absence (serum‐) of serum. Nuclei are highlighted with Hoechst. Insets: Higher magnification of the boxed regions in the fields of NIH3T3 serum‐ supplemented with N/L. Quantification of eMI binding (left) and degradation (right) in serum+ (e) and serum‐ (f) conditions. N ≥ 2500 cells from 2 independent experiments (ie). Data are mean ± SEM and individual values. One sample multiple t‐test relative to the control cells was used (e,f). Differences were significant for *p < 0.05, **p < 0.01, ***p < 0.001 and ****p < 0.0001

Among the top hits of the screen, we noticed that transcriptional repression of 3 components of the exocyst complex, previously linked to vesicle secretion (Guo et al., 1999; TerBush et al., 1996), led to significant changes in eMI activity (Figure S5c,d). The exocyst is an eight‐member protein complex organized into two different subcomplexes (SCI and SCII) (Figure 5a) (Ahmed et al., 2018), whose assembly and cellular localization are often regulated by the GTPases RalA and RalB (Moskalenko et al., 2003). The exocyst mediates tethering and localization of secretory vesicles to the plasma membrane, but some exocyst components have been reported to be associated with LE/MVBs (Monteiro et al., 2013). We confirmed that members of the two exocyst subcomplexes, as well as RalA and RalB, associate with LE/MVBs (Figure 5b,c).

Using NIH3T3 mouse fibroblasts stably expressing the KFERQ‐split Venus reporter, we analyzed the effect on different eMI steps of individual knock‐down (KD) of three members of exocyst SCI (Exoc2, Exoc3, and Exoc4), three members of exocyst SCII (Exoc5, Exoc6, and Exoc7) or of RalA (Figure S6a,b shows KD efficiency for each protein). We quantified the amount of reporter sequestered in the LE/MVB (binding/internalization) as the number of fluorescent puncta in cells incubated in the absence of protease inhibitors, and the amount of reporter undergoing eMI degradation as the increase in the number of fluorescent puncta per cell upon inhibition of endolysosomal proteolysis with N/L treatment (Caballero et al., 2018; Krause & Cuervo, 2021). We observed only minimal changes in eMI binding and trends to increased degradation upon knockdown of several exocyst subunits in cells maintained in the presence of serum (Figure 5d,e). However, upon serum deprivation, a condition shown to inhibit eMI (Krause et al., 2022), we found significant increases in both eMI substrate binding/internalization and degradation upon knockdown of components of exocyst SCI (Exoc3, Exoc4) and SCII (Exoc5, Exoc6) (Figure 5d,f). Pharmacological inhibition of exocyst activity with Endosidin 2 (ES2) (Zhang et al., 2016) in control cells stably expressing the KFERQ‐Split Venus reporter demonstrated a similar dose‐dependent stimulatory effect on eMI, mainly at the degradation step (Figure S6c). Interestingly, KD of the regulatory component RalA, although less efficient than for other exocyst components, displayed one of the most pronounced stimulatory effects (approx. 10‐fold increase over control cells) when eMI was repressed by serum deprivation, but it lacked any effect over basal (Serum+) eMI (Figure 5d–f), suggesting that RalA may be a key effector of the inhibition of eMI in the absence of nutrients. Overall, these findings are consistent with an inhibitory role for the exocyst complex and the RalA effector in eMI activity.

**FIGURE 6:**
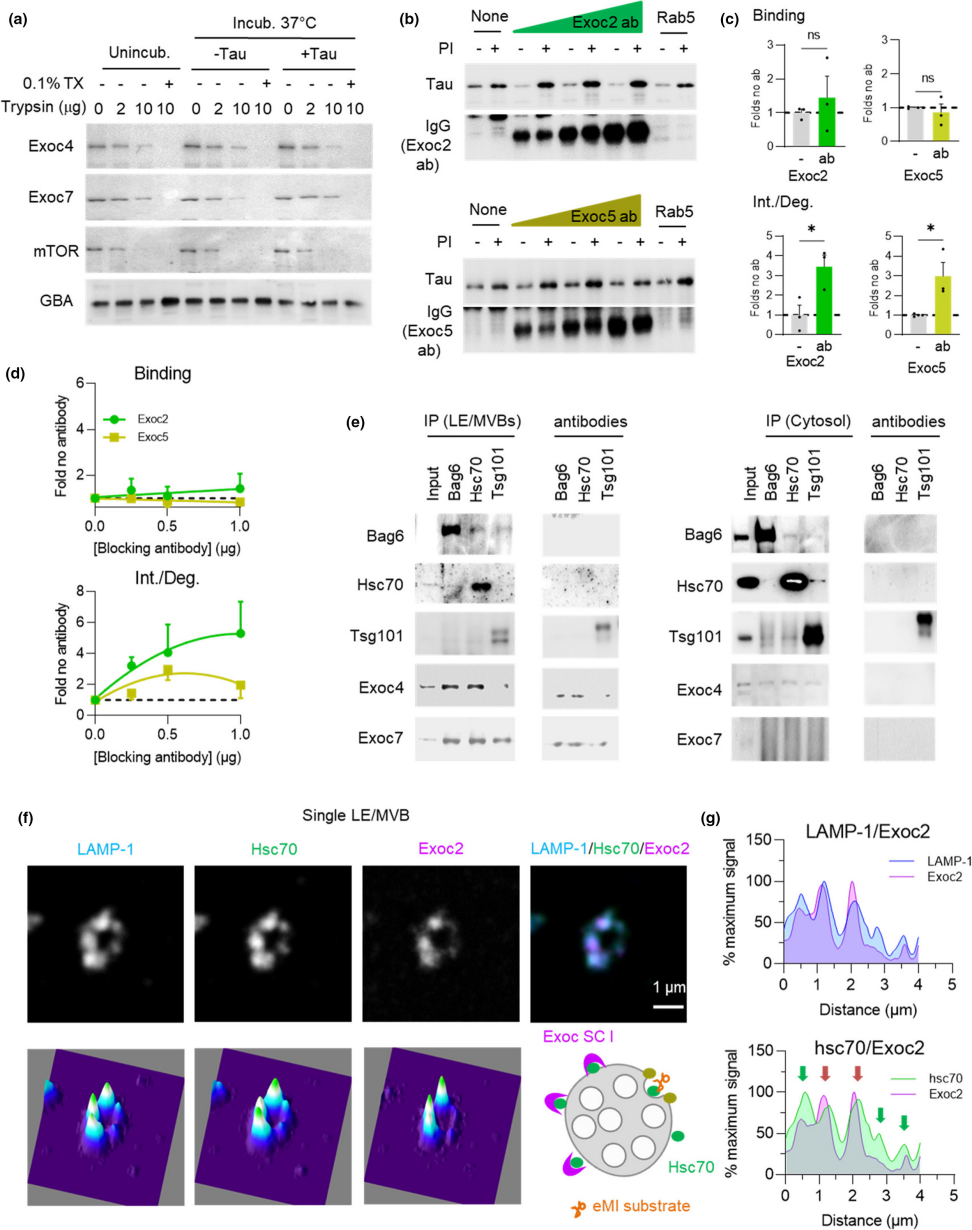
The inhibitory effect of the exocyst complex on eMI occurs at LE/MVBs. (a) LE/MVB topology of Exoc4 and Exoc7. Immunoblot of LE/MVBs incubated with the indicated concentrations of trypsin right after isolation (unincub.) or after incubation without (−) or with (+) recombinant tau protein. Where indicated, 0.1% triton X100 (TX) was added. N = 2 independent experiments (ie). (b–d) Representative immunoblots (b) of LE/MVBs preincubated or not with protease inhibitors (PI) and/or increasing concentrations of an antibody against Exoc2 or Exoc5 and then with tau. Incubation with antibody against Rab5 is shown as control. Immunoblot only with secondary antibody is shown to monitor binding of antibodies to the LE/MVB membranes. Quantification of the effect of eMI binding (top) and internalization/degradation (int./deg.) (bottom) with the concentration of antibody with maximal effect on eMI (c) and dose response curve for each antibody on eMI binding (d; top) and int./deg. (d; bottom). N = 4 (Exoc2), 3 (Exoc5) ie. (e) Immunoblots for the indicated proteins in LE/MVBs (left) and cytosol (right) immunoprecipitated (IP) with antibodies against Bag6, Hsc70 or Tsg101. Immunoblot of lines including only the antibodies are shown on the right and positive and negative controls for IP are shown in Figure S6d. N = 2 ie. (f–g) Representative τ‐STED microscopy images (f) of isolated LE/MVBs from a 4 m old mouse immunolabeled for the indicated proteins. Single channel black and white original images (top left), pseudocolored 3 channels merged image (top right) and 2.5‐D density plots (bottom). Quantification (g) of the intensity of LAMP‐1 and Exoc2 (top) and Hsc70 and Exoc2 (bottom) along the LE/MVB membrane. Scheme at bottom of (f) shows proposed membrane distribution for indicated proteins. N = 3 animals, ≥5 LE/MVBs per animal. Arrows indicate areas of high concentration for Hsc70 (green) and for both proteins (red). Data are mean ± s.e.m. and individual values are shown in (c). Unpaired two‐sided t‐test was used. Differences were significant for *p < 0.05, **p < 0.01. ns, not significant

We next set to elucidate if part of the inhibitory role of the exocyst complex on eMI activity occurred directly at the level of the individual LE/MVB. Trypsinization of isolated LE/MVBs to determine the topology of Exoc4 and Exoc7, as representative examples of components of exocyst SCI and SCII, respectively, showed that the majority of Exoc4 and Exoc7 was present at the surface of LE/MVBs (susceptible to trypsin proteolysis) (Figure 6a). We also detected a small fraction of each protein only degraded upon disruption of the membrane with detergent (Figure 6a), in support of their presence in the lumen of LE/MVBs. This luminal fraction was protected from degradation by LE/MVBs proteases, as it remained unchanged upon incubation of these organelles at 37°C (Figure 6a) and may represent the previously reported presence of exocyst components inside exosomes (Chacon‐Heszele et al., 2014). Activation of eMI by incubation of LE/MVBs with Tau protein did not affect the surface/lumen distribution of exocyst components (Figure 6a), suggesting that internalization of exocyst proteins may be independent of eMI and motivating us to focus on the surface associated exocyst proteins.

We used the in vitro eMI reconstitution assay to investigate the effect on eMI of pre‐incubating LE/MVBs with increasing concentrations of antibodies against Exoc2 (SCI) or Exoc5 (SCII) (Figure 6b; immunoblot confirmed efficient binding of both antibodies to the exocyst proteins to the LE/MVB surface). Antibody blockage of either Exoc2 and Exoc5 did not change Tau binding to LE/MVB but led to a significant dose‐dependent increase in Tau internalization/degradation (Figure 6b–d). These findings are consistent with the data from the cell KD experiments showing a modest effect on eMI binding and a substantial effect on eMI degradation.

Since exocyst complex inhibition of eMI occurs at the level of individual LE/MVBs, we analyzed possible interactions with eMI components in this compartment. Co‐immunoprecipitation for Bag6, Hsc70, Tsg101, or Alix from isolated LE/MVBs revealed that Exoc4 and Exoc7 co‐precipitated with Hsc70 and Bag6 (Figure 6e; RalA and LAMP‐1 are shown in Figure S6d as positive and negative control of Exoc4 interaction, respectively). Although limited by the availability of antibodies suitable for reverse co‐immunoprecipitation, we were able to at least confirm that pull‐down with anti‐Exoc4 co‐immunoprecipitated Hsc70 (Figure S6e). Interaction of Exoc4 or Exoc7 with Hsc70 and Bag6 was not detected in cytosol (Figure 6e), thus making it unlikely that their association in LE/MVBs was a result of Exoc4 or Exoc7 being eMI substrates themselves. Furthermore, none of the exocyst components tested underwent degradation in LE/MVBs (Figure S6f shows levels of Exoc2, 4 and 7 in LE/MVBs from mice injected or not with leupeptin).

Since we did not find major changes in the abundance of exocyst components in LE/MVBs with changes in eMI activity (Figure S6g,h shows comparable levels of Exoc2, Exoc4, Exoc7 and RalA in fed and starved mice, where eMI activity is reduced), we next investigated possible differences in their distribution at the LE/MVB membrane in these conditions. τ‐STED microscopy of isolated LE/MVBs immunostained for LAMP‐1, Hsc70, and Exoc2 (Figure 6f) identified regions of coincidence of Exoc2 and Hsc70 signal along the LE/MVB membrane, with discrete foci of Exoc2 compared with the uniform distribution of Hsc70 (Figure 6g). Given the inhibitory effect of the exocyst complex on eMI and the interaction of Exoc4 with Hsc70 and Bag6, we propose a model where the exocyst complex exerts its inhibitory effect on eMI through sequestration of part of the LE/MVB‐associated Hsc70, thus limiting its contribution to eMI (Figure 6f, lower right scheme).

### 2.6 Age‐related changes in exocyst complex organization in LE/MVBs

**FIGURE 7:**
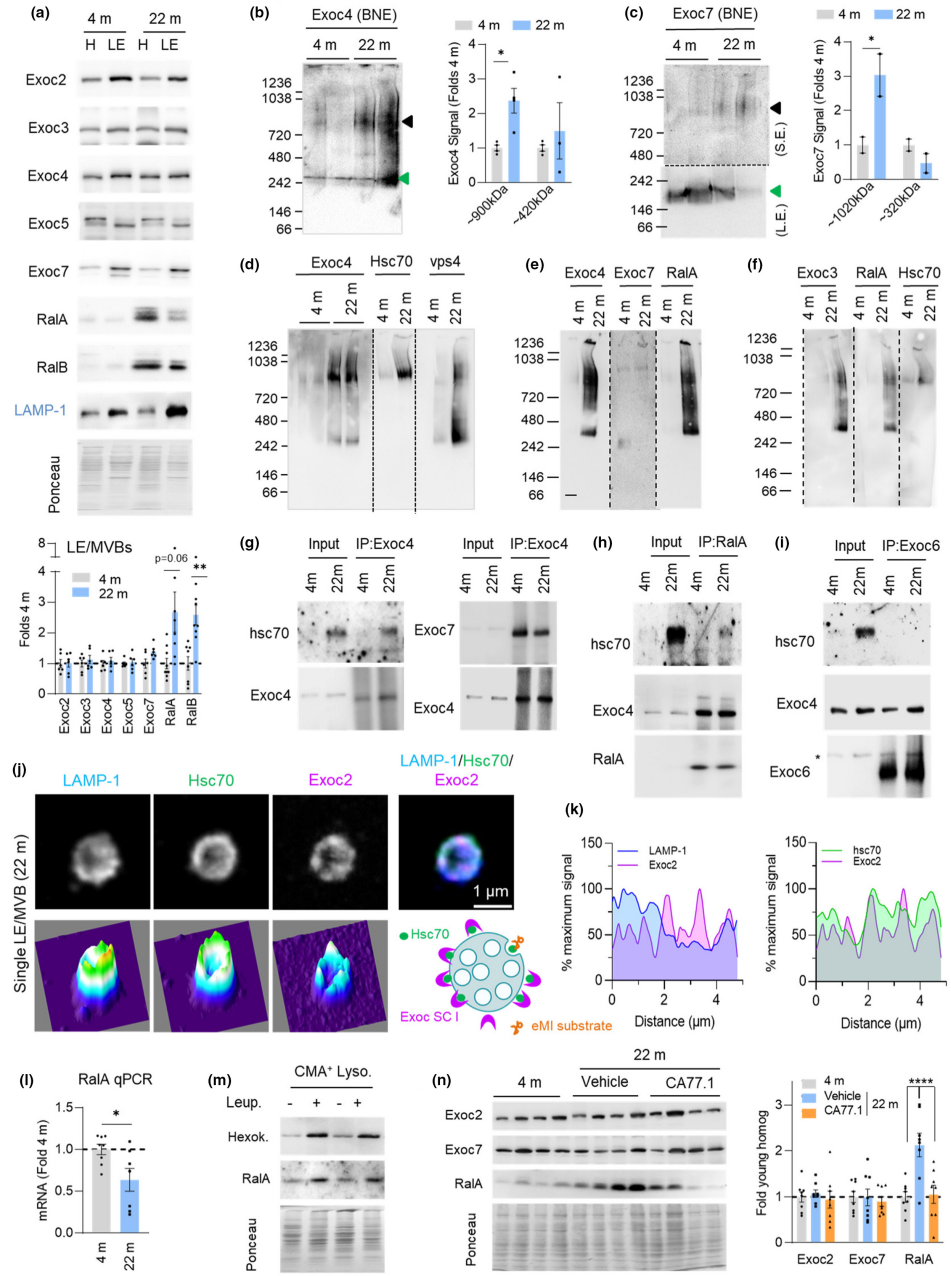
Age‐related changes in the multimeric state of the exocyst in LE/MVBs. (a,b) Representative immunoblots (top) and quantification (bottom) of abundance of the indicated exocyst components in homogenate (H) and LE/MVBs (LE) from 4 m and 22 m old mouse livers. Values are expressed relative to those in 4 m old LE/MVBs. N = 6–9 mice. (b,c) Representative immunoblots for Exoc4 (b) and Exoc7 (c) after blue‐native electrophoresis of 4 m and 22 m old LE/MVBs. Images of the top of the membrane in c after short exposure (S.E.) (top) and the bottom half of the membrane after long exposure (L.E.) (bottom) are shown. Black discontinous line indicates the different exposure regions. Right shows quantification of the abundance of the proteins in the different complexes. Arrows: Black (full exocyst complex), green (subcomplex I in b and II in c). N = 4 (Exoc4) and 2 (Exoc7) mice. (d–f) Representative immunoblots for the indicated proteins after blue‐native electrophoresis of 4 m and 22 m old LE/MVBs. Samples in each of the panels were run in the same gel and black dotted lines indicate where membranes were cut to blot for each protein separately. (g–i) Immunoblots for the indicated proteins in LE/MVBs from 4 m and 22 m old mice subjected to immunoprecipitation (IP) with antibodies against Exoc4 (g), RalA (h) or Exoc6 (i). N = 2 ie. (j,k) Representative τ‐STED microscopy images of isolated LE/MVBs from a 22 m old animal immunolabeled for the indicated proteins. Single channel black and white original images (top left), pseudocolored 3 channels merged image (top right) and 2.5‐D density plots (bottom) (j). Quantification (k) of the intensity of LAMP‐1 and Exoc2 (top) and Hsc70 and Exoc2 (bottom) along the LE/MVB membrane. Scheme at bottom of (j) shows proposed membrane distribution for indicated proteins. N = 3 animals, ≥5 LE/MVBs per animal. (l) Quantification of RalA mRNA in livers of 4 m or 22 m old mice. Values are expressed relative to those in 4 m old mice. N = 8 (4 m) and 7 (22 m) mice. (m) Immunoblot for the indicated proteins in lysosomes active for CMA (CMA^+^ Lyso) isolated from mice injected with saline (−) or leupeptin (+). Hexokinase is shown as a control of a known CMA substrate. N = 3 mice per condition. (n) Representative immunoblot for the indicated proteins (left) and quantification (right) of liver homogenates from 4 m and 22 m old mice after 5 months of daily oral administration of jelly pills with vehicle or the chemical CMA activator CA77.1, starting at 18 months. N = 8 mice. All data are mean ± SEM and individual values. Ponceau staining is shown as loading control in the immunoblots. One sample multiple t‐tests (a–c), unpaired two‐sided t‐test (l) and two‐way ANOVA with Bonferroni's multiple comparisons post hoc test (n) were used. Differences were significant for *p < 0.05, **p < 0.01, ****p < 0.0001. ns, not significant

Given the inhibitory effect of the exocyst complex on eMI, we investigated if changes in this complex with age may underlie some of the reduction in eMI with aging. While overall levels of the tested exocyst components (Exoc2, Exoc3, Exoc4, Exoc5, and Exoc7) did not change with age, total cellular levels and LE/MVB levels of both regulatory components RalA and RalB increased over twofold with age (Figure 7a and S7a). Since RalA and RalB have been shown to regulate localization and assembly of the exocyst complex (Zago et al., 2019), we performed blue‐native electrophoresis of young and old LE/MVBs to investigate the oligomeric status of their exocyst complexes. Immunoblot for Exoc4 (SCI) and Exoc7 (the only component of SCII for which we found antibodies able to recognize the native protein) revealed increased abundance of high molecular weight complexes (approximately 900 kDa for Exoc4 and 1020 KDa for Exoc7) in 22 m old LE/MVBs (Figure 7b,c black arrow). The lower molecular weight complex for both proteins (green arrow) may indicate the four‐member exocyst subcomplexes, whereas the high molecular weight band may represent homo or hetero octameric exocyst complexes (~734 kDa) bound to additional proteins (Ahmed et al., 2018). In fact, the Exoc4 high molecular weight complex detected in old LE/MVBs was also positive for Hsc70 (Figure 7d) and coincided in size with the Hsc70‐positive protein complex that we described to be present mostly in old LE/MVBs (Figure 2h). A fraction of Vps4 (Figure 7d), RalA and other components of SCI such as Exoc3 (Figure 7e,f) were also detected in this same high molecular weight region in higher abundance in old LE/MVBs, in further support of disrupted dynamics of both the exocyst complex and eMI machinery with age. Although resolution of the precise size of the different complexes for each of these proteins will require more accurate procedures, our blue‐native electrophoresis studies support coincidence of subunits of SCI, RalA, and Hsc70 in similar size complexes (in the 890–950 kDa range), whereas Exoc7 preferentially distributed in a higher molecular weight complex (1020 kDa) along with a small fraction of Exoc4 (Figure S7b shows the high molecular weight region in more detail).

To confirm direct interaction of the exocyst/RalA components with Hsc70 in LE/MVBs and to determine if the Hsc70 observed in these complexes was the glycosylated form of the chaperone, we performed co‐immunoprecipitation experiments in LE/MVBs. We found that glycosylated Hsc70 co‐precipitated with both Exoc4 (Figure 7g, left) and RalA (Figure 7h) (note that the unmodified hsc70 is barely visible to avoid saturation of the glycosylated form). The presence of glycosylated Hsc70 in the co‐immunoprecipitated fractions was not a contamination because it was not observed, for example, in similar pull‐down experiments for Exoc6 (the only subunit of SCII with antibodies suitable for immunoprecipitation), although Exoc6 was still interacting with a fraction of LE/MVB Exoc4 (Figure 7g right and 7i). These findings are in agreement with the blue‐native electrophoresis data, that placed hsc70 mostly in RalA/SCI complexes (Figure 7d,f and S7b).

Since a fraction of Hsc70 in LE/MVBs interacts with exocyst SCI (Figure 7g) and our knock‐down and τ‐STED experiments suggest that this interaction may be inhibitory on eMI, we postulated that the age‐dependent glycation of Hsc70 may change Hsc70/exocyst SCI dynamics at the membrane of old LE/MVBs and contribute to reduced eMI. Analysis of the distribution of Hsc70 and Exoc2 in isolated 22 m old LE/MVBs using τ‐STED microscopy (Figure 7j,k) and comparison with their distribution in 4 m old LE/MVBs (Figure 3a–c), revealed that Exoc2 is more diffusely distributed in the membranes of old LE/MVBs and displays higher overlap with Hsc70. We propose that sequestration of a higher fraction of LE/MVB‐Hsc70 by the exocyst SCI may decrease the availability of free Hsc70 for eMI with age (Figure 7j, lower right scheme).

To further characterize the changes in association of exocyst SCI and SCII within individual LE/MVBs with age, we performed immunofluorescence for Exoc2 and Exoc7, as representative members of each subcomplex, in spotted LE/MVBs previously incubated with Tau protein (to identify eMI‐active LE/MVBs as those positive for LAMP‐1 and the substrate Tau). We found that the fraction of eMI‐competent LE/MVBs that contained Exoc2 significantly increased with age (18.3% in 4 m old vs. 33.5% in 22 m old) while those containing Exoc7 remained unchanged (9% in 4 m old vs. 11.5% in 22 m old) (Figure S7c,d). These findings suggest that the association of exocyst SCI to eMI‐competent LE/MVBs changes with age and that, because the overall abundance of Exoc2 in LE/MVBs does not change with age (Figure 7a), the increased colocalization of Exoc2 with eMI‐competent LE/MVBs may result from its redistribution from LE/MVBs not engaged in eMI. Immunostaining for RalA in spotted LE/MVBs processed in the same conditions revealed a very marked age‐related increase in the association of RalA with LAMP‐1^+^/Exoc7^+^ vesicles (from 17.6% in 4 m old to 53.8% in 22 m old) (Figure S7e). These findings are in agreement with the increase in RalA levels in LE/MVBs with age (Figure 7a) and higher abundance of octameric exocyst complexes (Figure 7b‐f), which could mediate the observed bias with age of LE/MVBs from degradative to secretory compartments (Figure 4).

The pronounced physiological inhibitory effect of RalA on eMI, whereby a 30% reduction in RalA levels led to >10‐fold increase in eMI degradation (Figure 5d–f), suggests that even small changes in RalA levels can have a marked impact on eMI activity and made us consider that the increase in RalA cellular levels with age (Figure 7a) could be a key contributor to the decline of eMI in aging. To investigate the mechanism(s) behind increased RalA levels in aging, we first analyzed possible transcriptional upregulation, but qPCR analysis revealed instead a significant reduction with age in RalA mRNA (Figure 7l). We instead considered changes in RalA degradation with age and noticed the presence of a KFERQ‐like motif in its sequence (^197^KRIRE^201^ becomes a motif upon acetylation of the lysine residue). Since our proteomic analysis (Figure 2 and S7f) did not support degradation of RalA in LE/MVBs and given that KFERQ pentapeptides are shared as targeting motifs for both eMI and CMA (Krause & Cuervo, 2021), we investigated if RalA could be degraded by CMA. We found that a fraction of RalA could be detected in CMA active lysosomes and that in vivo injection of leupeptin resulted in an accumulation of RalA in these lysosomes (Figure 7m), a behavior characteristic of CMA substrates (Hexokinase is shown as an example of a validated CMA substrate). Using LE/MVBs isolated from livers of CMA‐deficient mice (knock‐out for the limiting CMA component LAMP‐2A), we confirmed that blockage of CMA‐dependent degradation of RalA led to significantly higher association of RalA with LE/MVBs (Figure S7g) and led to an accumulation of RalA in total liver homogenate from LAMP‐2A KO mice (Figure S7h). Conversely, pharmacological activation of CMA in old mice, where CMA activity is markedly reduced (Cuervo & Dice, 2000), normalized RalA to the levels observed in young mice. Daily oral administration of CA77.1, a novel, specific CMA activator suitable for in vivo use (Bourdenx et al., 2021) in old mice significantly reduced the age‐related accumulation of RalA without affecting levels of Exoc2 (SCI) or Exoc7 (SCII) (Figure 7n).

Overall, our findings are consistent with an inhibitory role of the exocyst complex in eMI activity and with RalA‐dependent changes with age in exocyst complex dynamics in LE/MVBs, likely as a result of its interaction with glycosylated Hsc70, that result in an inhibitory effect on eMI activity (Figure S8). Defective degradation of RalA by CMA provides a basis for the age‐related accumulation of RalA and places CMA as a key regulator of eMI activity. The age‐related increase in the organization of exocyst proteins into complexes may reflect both a mechanism for the reduced eMI activity observed in aging and a link between eMI and protein secretion when substrates can no longer be effectively degraded by the pathway (Figure S8).

## 3 DISCUSSION

This work provides evidence for reduced eMI activity with age and identifies the causes and possible consequences of the failure of this component of the proteostasis network. We propose that the age‐related post‐translational modification of the eMI chaperone Hsc70 in LE/MVBs contributes to the observed alterations in the stability and membrane dynamics of the chaperone and other eMI regulators in this compartment. Added to the changes in the intracellular proteome, we found that the decline in eMI activity in aging is matched with elevated extracellular release of proteins and identify the exocyst complex and the GTPase RalA as novel inhibitors of eMI activity and possible mediators of the link between reduced eMI degradation and elevated protein secretion in aging.

Our work supports that the reduction in eMI activity with age occurs at the level of substrate internalization and degradation, whereas targeting of eMI substrate proteins to LE/MVBs remains, for the most part, unchanged. These findings resemble those described for CMA, the type of autophagy most closely related to eMI, where proper targeting of substrate proteins by Hsc70 to lysosomes was still observed in old organisms (Cuervo & Dice, 2000). However, while in CMA, the decrease with age in levels of the lysosomal receptor for this pathway makes substrate binding to lysosomes the first affected step in aging (Cuervo & Dice, 2000), in the case of eMI we found that substrate internalization and mostly degradation in LE/MVBs are the main altered steps with age. In fact, reduced binding only becomes apparent after exposure of old LE/MVBs to an excess of substrate protein in vitro (Figure 1b,c), but was not observed under physiological conditions, where instead a trend toward higher binding with age (Figures 1h,i) supports defective substrate internalization and degradation. One limitation of currently available tools and assays to monitor eMI is their inability to fully discriminate sequestration of cargo in membrane forming vesicles from complete pinching off and internalization in these vesicles, which forces the use of degradation as ultimate evidence of internalization. The use of τ‐STED microscopy in this work helps to partially circumvent this limitation by allowing the discrimination of proteins in the membrane and luminal regions. However, some of the recently formed vesicles could still remain too close to the membrane to attribute them a luminal localization. Nevertheless, combination of the biochemical and image‐based approaches in this work supports our conclusions of reduced eMI substrate internalization and degradation with age. Reduced internalization of substrates necessarily leads to decreased degradation, but we also show a primary defect in proteolysis of substrates in the lumen of old LE/MVBs, independent of their internalization (Figure 1k). Although aged LE/MVBs have a more acidic pH and higher levels of proteolytic enzymes (Figures 1l and S3j), the capacity of those enzymes to degrade substrates was sharply reduced. Future studies are required to clarify the basis of this reduced enzymatic activity and whether age‐related post‐translational modifications in the enzymes, as the ones described here for Hsc70, could contribute to it. Previous studies have shown that part of the degradation of eMI internalized substrates occurs directly in LE/MVBs whereas part is attained upon fusion of these compartments with lysosomes (Sahu et al., 2011). The reduced ability of LE/MVBs to degrade internalized cargo with age may increase the need for fusion with lysosomes at a time when lysosomes have also been shown to have lower degradative capability (Nixon, 2020), further contributing to overwhelming of the autophagic/lysosomal system.

Changes with age in the eMI chaperone Hsc70 may partially explain the reduction in eMI activity in aging. We propose that the high abundance of glycated Hsc70 in aged LE/MVBs (Figure 2) is behind the altered membrane dynamics and reduced stability of Hsc70 in LE/MVBs during aging. Although this abnormal behavior was noticed for both glycated and non‐glycated forms of Hsc70, it is likely that the reported self‐interaction (dimerization and oligomerization) of this chaperone (Takakuwa et al., 2019), is behind the malfunctioning of the unmodified Hsc70 when it interacts with the glycated variant. Since the age‐related reduction in eMI does not associate with reduced substrate targeting, it is unlikely that glycation of Hsc70 affects its ability to recognize KFERQ‐like motifs (Kirchner et al., 2019) of substrate proteins or reduces hs70 interaction with phosphatidylserine at the LE/MVB required for substrate delivery to this compartment (Morozova et al., 2016). Instead, we propose that glycation of Hsc70 leads to persistent Hsc70 interactions with other components in the LE/MVB membrane, reducing its availability for eMI or affecting the stoichiometry of other eMI regulators, such as Vps4, exocyst subunits, and RalA. This would explain both the toxic effect of Hsc70 glycation on eMI and the accumulation of glycated Hsc70 in the LE/MVB compartment, despite a fraction of Hsc70 undergoing faster degradation in old LE/MVBs.

The direct functional consequences of each of the glycosylation sites detected in Hsc70 from old LE/MVBs requires future study. Unfortunately, experimental deglycosylation of Hsc70 in intact LE/MVBs is not possible because the denaturing conditions disrupt the integrity of this compartment. Although mutagenesis of the specific glycosylated residues is needed to assess precisely how age‐related glycosylation of Hsc70 at each of the detected sites affects eMI activity, based on their placement in the Hsc70 3‐D structure (Figure S2d), we propose that glycosylation could interfere with its ATPase activity, interaction with Bag6, and Hsc70 binding to phosphatidylserine residues at the LE/MVB membrane, all properties closely linked to the known roles of Hsc70 in eMI.

An additional outstanding question is where Hsc70 age‐related glycosylation takes place. Based on the findings that (i) glycosylated Hsc70 is barely detected in cytosol from old mice, (ii) eMI substrate targeting and binding to LE/MVBs is unaffected by aging, and (iii) glycosylated Hsc70 is highly enriched in LE/MVBs, we propose that Hsc70 undergoes this modification directly in the LE/MVB compartment. It is possible that this modification could be enzymatically mediated (glycosylation) as result of mistargeting/leakage of Golgi glycosylating enzymes to LE/MVBs with age. However, we favor the possibility that Hsc70 undergoes non‐enzymatic glycosylation (glycation) directly in LE/MVBs, as oxidatively modified proteins with advanced glycation end‐products have been previously described to accumulate in this compartment with age (Cannizzo et al., 2012). Local occurrence of Hsc70 glycation may restrict its impact to LE/MVBs, leaving earlier endocytic pathways unaffected by this modification. Of note, cytosolic protein aggregation, as observed in neurodegenerative conditions, has proven to be inhibitory on clathrin‐mediated endocytosis because sequestration of Hsc70 in protein aggregates reduced the availability of this chaperone for removal of clathrin from early endocytic vesicles (Yu et al., 2014). In the case of LE/MVBs, we observed instead an increase in Hsc70 levels with age (Figure 2a), suggesting that either different endocytic process are differentially affected by age or that the chaperone depletion observed in the context of neurodegeneration may not occur until more advanced ages than tested here or only in instances with high levels of cytosolic protein aggregation. Future studies will determine the possible contribution of the failure of endocytosis in those instances to eMI function.

A growing number of examples support that if proteins are unable to be degraded due to a blockage in the intracellular proteolytic systems, a last resort to rid the cell of toxic products could be to secrete them outside of the cell (Caballero et al., 2021; Ferreira et al., 2019; Solvik et al., 2022). Different mechanisms contribute to this release of undegraded materials, likely dependent on the origin of the proteolytic blockage. Failure to acidify lysosomes has been shown to increase secretion of proteins in extracellular vesicles in a macroautophagy‐dependent manner (secretory autophagy) (Solvik et al., 2022). In contrast, selective inhibition of CMA leads to rerouting of substrate proteins toward LE/MVBs via eMI and subsequent extracellular release in exosomes upon docking of LE/MVBs with the plasma membrane (Caballero et al., 2021). Here, we demonstrated that protein secretion is elevated during aging using primary fibroblasts from young and old mice and that this secretion occurs in a manner independent of conventional secretion mechanisms based on the lack of sensitivity to Brefeldin A treatment (Figure 4). The studies in the first part of this work were all done in isolated liver LE/MVBs because the amount and purity of the recovered organelles allows for the reconstitution of eMI in vitro to directly analyze changes with age in this pathway separately from other age‐related changes. In contrast, the studies on protein secretion were performed in primary fibroblasts in culture given their suitability for collection of ECVs from a single cell type. However, the fact that elevation in secretion was reproduced by acute blockage of proteolysis in young primary mouse fibroblasts helped link the observations in the two different experimental systems and suggests that the inability to degrade proteins may trigger their extracellular release rather than risk their intracellular accumulation. Future studies comparing degradation and secretion in the same model system are needed to elucidate the molecular mechanisms behind the crosstalk between both processes.

The absence of the previously reported increase in autophagy receptors in the secreted material when released through secretory autophagy (Solvik et al., 2022), along with the marked increase of RalA and other exocytosis‐related components in LE/MVB with age, make us propose exocytosis as one of the major contributors to the observed increase in protein secretion with age. Interestingly, a recent study has shown that inhibition of proteolysis with chloroquine in several mammary cancer cell lines led to changes in cargo released in EVs but not in the total amount of secreted protein in these vesicles (Xu et al., 2022). It is possible that consequences of endo/lysosomal proteolysis inhibition are different between primary, untransformed cells and cancer cells, as the rapid division of the latter makes protein quality control less of a priority. Alternatively, failure to increase protein content in EVs in that study could be due to the previously reported inhibitory effect of chloroquine on protein synthesis (Ciak & Hahn, 1966), not observed with the endolysosomal proteolysis inhibitors used in our work (leupeptin and ammonium chloride).

We have uncovered a previously unknown role for the exocyst complex and the GTPase RalA in regulating eMI activity (Figures 5 and 6). The exocyst complex inhibits eMI substrate internalization and degradation directly at the LE/MVB membrane. This inhibition may be mediated through an interaction with the eMI chaperones Hsc70 and Bag6 based on co‐precipitation of Exoc4 and 7 with these chaperones in the LE/MVB compartment. Elucidating the exact mechanism of exocyst inhibition of eMI activity will require future studies, but based on our findings we favor a model where the exocyst complex, by interacting with Hsc70 and Bag6, limits the amount of these chaperones for eMI and thus limits substrate loading into intraluminal vesicles. Despite the potent inhibitory effect of RalA on eMI in cultured cells (Figure 5), pre‐incubation of LE/MVBs with an antibody against RalA did not result in changes in substrate internalization or degradation in the in vitro system. Although the lack of effect in vitro could be related with failure of the only selective antibody available to block RalA function, we propose that recruitment of RalA to LE/MVBs exerts its inhibitory effect on eMI, at least in part, by directing this organelle to fusion with the plasma membrane. Although the effect of RalA on eMI could be independent on the exocyst, we favor an exocyst‐dependent effect based on the well‐known regulatory role of RalA in exocyst complex localization and assembly (Moskalenko et al., 2003; Zago et al., 2019), and the marked increase in the amount of high molecular weight exocyst complexes that we observed in old LE/MVBs, where the most pronounced change in exocyst‐related protein abundance was for RalA (Figure 7). Future studies are needed to dissect the protein composition of the high molecular weight complexes of SCI and SCII that accumulate in old LE/MVB (Figure 7); however, the fact that similar size complexes, albeit less abundant, are also visible in young LE/MVBs supports that they may be a result of enhanced assembly or reduced disassembly, rather than non‐specific interactions with other proteins at the old LE/MVB membrane. Since glycosylated Hsc70 can be precipitated with SCI components and RalA, we propose that this post‐translational modification may be the culprit of the disrupted kinetics of SCI and RalA in LE/MVBs with age.

RalA‐mediated exocyst assembly may be one mechanism through which LE/MVBs can be transitioned from a degradative to a secretory compartment and explain the relationship between reduced degradation and increased secretion observed in this work (Figures 6 and 7). The exocyst complex has been shown to work both independently (Monteiro et al., 2013) or in conjunction with RalA (Wang et al., 2004) to facilitate vesicle fusion with the plasma membrane. It is unclear which of these mechanisms is primarily responsible for the elevated protein secretion observed in aging, although the above‐mentioned coincidence of higher RalA levels and enhanced exocyst complex assembly in old LE/MVBs makes it likely that the exocyst and RalA act coordinately in the switch from eMI degradation to secretion. Future studies are needed to determine if the high molecular weight complex containing subunits of SCI and SCII but free of Hsc70 is enough to promote secretion. Alternatively, the Hsc70‐SCI‐RalA complex detected in higher abundance in old LE/MVBs, could be the one targeting these organelles to SCII containing regions of the plasma membrane to promote full exocyst assembly and vesicular content release.

We found it interesting that the observed increase with age in cellular levels of RalA can be in large part explained by a decrease in its degradation through CMA (Figure 7), providing an additional point of interplay between these two selective types of autophagy. CMA‐dependent degradation of RalA may be a way to titrate eMI activity, as we found that small changes in RalA levels can have a dramatic effect on this pathway (Figure 5) and to coordinate CMA and eMI activities. Furthermore, this previously unknown degradation of RalA by CMA highlights a possible impact of changes in CMA activity on the many cellular processes regulated by RalA (Yan & Theodorescu, 2018). Finally, the efficient normalization in RalA levels upon chemical activation of CMA provides a potential mechanism to reduce release of undegraded or toxic protein products that fail to undergo degradation through eMI. This may have therapeutic potential in the context of neurodegenerative diseases as a way to limit the extracellular release of pathogenic proteins like Tau and α‐synuclein in favor of their intracellular degradation, which may help limit the propagation of disease (Miranda & Di Paolo, 2018).

Overall, this work establishes that eMI, like other proteostasis pathways, declines in function with age and provides possible mechanisms and consequences for this decline. The characterization of the exocyst complex and RalA as endogenous inhibitory modulators of eMI further adds to our still limited knowledge of how this pathway is regulated and establishes a link between eMI and protein secretion, which is used by aging cells to ameliorate proteotoxicity.

## 4 EXPERIMENTAL PROCEDURES

### 4.1 Animal models and cell culture

This study used adult (3–6 months) male Wistar rats (Charles River Laboratories) and male young (4–6 months) and old (22–24 months old) mice (NIA colony), and mice with systemic knockout for LAMP‐2A (L2A^−/−^) (Schneider et al., 2014). All animal experiments were under an animal study protocol approved by the Institutional Animal Care and Use Committee of the Albert Einstein College of Medicine. In vitro studies were done using *NIH3T3 mouse fibroblasts* from the American Type Culture Collection (ATCC) or mouse primary ear fibroblasts from 4 m and 22 m old mice, isolated as described previously (Varela et al., 2005). Knock‐down of the proteins of interest was performed using lentivirus containing shRNAs listed in Table S1. Details on animal maintenance and treatments and on cell culture conditions and in vitro treatments are provided under Extended Experimental Procedures (Appendix S1).

### 4.2 Chemicals and antibodies

Sources of chemicals and dilutions and sources of antibodies used in this study are detailed under Extended Experimental Procedures (Appendix S1).

### 4.3 Studies on isolated organelles

Rodent liver lysosomes and LE/MVBs were isolated through differential centrifugation and separation into discontinuous density gradients. Cytosol was prepared by centrifugation at 100,000×*g* for 1 h of the post‐nuclear supernatant. Extracellular vesicles (ECVs) were isolated from the culture media by sequential centrifugation and concentration through molecular exclusion columns. LysoSensor Yellow/Blue DND‐160 was used for pH determination, PNGase F and Endo H for deglycosylation, trypsinization to determine protein topology and incubation at physiological temperature and immunoblot to determine protein stability as described in detail under Extended Experimental Procedures (Appendix S1).

### 4.4 eMI measurements

In vitro analysis of eMI was performed using isolated *LE/MVBs* and purified substrate proteins as described before (Krause & Cuervo, 2021). Binding was calculated as the amount of protein associated with LE/MVBs untreated with protease inhibitors and internalization/degradation as the difference between proteins present in organelles treated with protease inhibitors after subtracting for the amount bound. *Proteolysis* in isolated LE/MVBs was assayed using a pool of radiolabeled proteins as before (Sahu et al., 2011). Measurement of eMI in cultured cells was done using the KFERQ‐Split Venus reporter (Caballero et al., 2018) described in detail under Extended Experimental Procedures (Appendix S1).

### 4.5 Protein analysis procedures

*Protein secretion* was measure upon ^3^H‐leucine metabolic labeling of cultured cells by analysis of radioactivity on the acid precipitable fraction collected from the culture media. *Co‐immunoprecipitation* was performed upon solubilization in a mild detergent/low salt buffer using primary antibodies and Protein A/G Plus agarose beads. *Protein electrophoresis and immunoblot, quantitative proteomics, and protein pathway analysis and glycoproteomics* were performed using standard procedures described in detail under Extended Experimental Procedures (Appendix S1).

### 4.6 Image‐based procedures

*Immunofluorescence* was performed in glass spotted organelles, fixed, and incubated with primary and secondary antibodies following standard procedures. *STED microscopy* of isolated organelles was performed in a Leica TCS SP8 STED 3X outfitted with a τ‐STED module and analysis of membrane/lumen distribution and organization of proteins in hot spots based on protein abundance were performed as described before (Krause et al., 2022) and detailed under Extended Experimental Procedures (Appendix S1).

### 4.7 CRISPRi screen and RNA quantification

*CRISPR interference (CRISPRi) screen* with an sgRNA library targeting 1176 genes related to proteostasis pathways was performed using cells expressing nuclease‐dead form of Cas9 fused to the transcriptional repressor KRAB (dCas9‐KRAB) and the KFERQ‐Split Venus fluorescent eMI reporter as described in detail under Extended Experimental Procedures (Appendix S1). *Quantitative RT‐PCR* was performed after reverse transcription of RNA into cDNA using the primers shown in Table S2 following isolation of total RNA using the RNeasy Plus kit (Qiagen) according to the manufacturer's instructions.

### 4.8 Quantification, statistical analysis, and software

All data presented are mean ± SEM and individual values. Detailed description of the statistical analysis and software used for acquisition, analysis and presentation of data can be found under Extended Experimental Procedures (Appendix S1). Raw data and statistical analyses from data presented in main and supplementary figures are provided as a Raw Data Excel file (Appendix S1) and uncropped images of the unprocessed immunoblots shown in all figures of the manuscript are compiled as figure in Appendix S1.

## AUTHOR CONTRIBUTIONS

GJK designed and performed most of the experiments, analyzed and interpreted the data and wrote the first draft of the manuscript; AD performed high content microscopy, metabolic labeling for intracellular protein degradation and secretion, cell culture and lentiviral packing; MJ isolated lysosomal fractions and assisted with LE/MVBs isolation; RRK performed the experiments with CA77.1 treatments; EA‐P and JJ B‐C advised with experimental design and assisted with τ‐STED microscopy experiments; OS‐F prepared the dermal mouse ear fibroblasts; ALR, KC, NJK and DLS performed and analyzed comparative LE/MVB proteomics; KA, KMM and MG performed and analyzed the nFCM NanoAnalyzer tracking of ECVs; PD performed and analyzed confocal microscopy experiments in isolated LE/MVBs; YS and SS performed and analyzed targeted proteomics for post‐translational modifications in Hsc70; MK conceived, coordinated and directed the CRISPRi screen; AMC conceived, coordinated and directed the study, contributed to manuscript writing and edited the final version of the manuscript.

## FUNDING INFORMATION

This work was supported by grants from the National Institutes of Health/National institute on Aging AG021904, AG054108 (to AMC), AG031782 (to AMC and JJBC) and AG062359 (to MK), National institutes of Health/National Institute of Diabetes and Digestive and Kidney Diseases DK098408 (to AMC), National Institutes of Health/National Institute on Neurological Disorders and Stroke NS100717 (to AMC and NJK) and NS095435 (to DS) and National Institutes of Health/National Cancer Institute CA244780 (to JJBC), the Tisch Cancer Institute NIH Cancer Center grant P30‐CA196521 (to JJBC), the NIH Office of the Director 1S10OD030286‐01 grant (to SS), AFAR Sagol Network GerOmics Award (to SS), Deerfield Xseed award (to SS) and the generous support of the Rainwaters Foundation (to AMC and MK), the JPB Foundation and the Glen Foundation (to AMC) and of Relay Therapeutics and Merk (to SS). GJK was supported by NIH training grants T32GM007288 and T32GM007491, MJ by T32HL14445, PD by T32GM007288 and RRK by the IRACDA program grant K12 GM102779. Stimulated emission‐depletion microscopy was performed in the Microscopy and Advanced Bioimaging CoRE at the Icahn School of Medicine at Mount Sinai, supported with funding from NIH Shared Instrumentation Grant (FAIN: S10OD021838). KA, KMM, and MG were supported by the NIA IRP, NIH.

## CONFLICT OF INTEREST

AMC is the founder and serves in the Scientific Board of Selphagy (a program under Life Biosciences) and she consults for Generian Pharmaceuticals and Cognition Therapeuticals. The NJK laboratory has received research support from Vir Biotechnology and F. Hoffmann‐La Roche. NJK has consulting agreements with the Icahn School of Medicine at Mount Sinai, New York, Maze Therapeutics and Interline Therapeutics, is a shareholder of Tenaya Therapeutics and has received stocks from Maze Therapeutics and Interline Therapeutics. DLS has a consulting agreement with Maze Therapeutics. MK is an inventor on US patent 11,254,933 related to CRISPRi/a screening; serves on the Scientific Advisory Boards of Engine Biosciences, Casma Therapeutics, Cajal Neuroscience and Alector; and is a consultant to Modulo Bio and Recursion Therapeutics. The rest of the authors declare no competing interests in relation to this work.

## Supporting information


Appendix S1

## Data Availability

There are no restrictions on data availability in this manuscript. All the information is included in the manuscript. All Main and Supplementary Figures have associated raw data that are provided as an Excel worksheet organized by figures and it includes statistics along with exact p‐values. The raw data of the proteomic analysis of LE/MVBs have been deposited to the ProteomeXchange Consortium via the PRIDE partner repository with dataset identifier PXD035128. Mass spectrometry raw files for the glycosylation analysis have been uploaded on the public repository Chorus (https://chorusproject.org) at the project number 1779. This manuscript does not report original code. Further information and requests for reagents may be directed to and will be fulfilled by the co‐corresponding author Ana Maria Cuervo (ana-maria.cuervo@einsteinmed.edu) upon completion of a Material Transfer Agreement.

## References

[acel13713-bib-0001] Ahmed, S. M. , Nishida‐Fukuda, H. , Li, Y. , McDonald, W. H. , Gradinaru, C. C. , & Macara, I. G. (2018). Exocyst dynamics during vesicle tethering and fusion. Nature Communications, 9(1), 5140. 10.1038/s41467-018-07467-5 PMC6277416 30510181

[acel13713-bib-0002] Bejarano, E. , Murray, J. W. , Wang, X. , Pampliega, O. , Yin, D. , Patel, B. , Yuste, A. , Wolkoff, A. W. , & Cuervo, A. M. (2018). Defective recruitment of motor proteins to autophagic compartments contributes to autophagic failure in aging. Aging Cell, 17(4), e12777. 10.1111/acel.12777 29845728 PMC6052466

[acel13713-bib-0003] Bourdenx, M. , Martín‐Segura, A. , Scrivo, A. , Rodriguez‐Navarro, J. A. , Kaushik, S. , Tasset, I. , Diaz, A. , Storm, N. J. , Xin, Q. , Juste, Y. R. , Stevenson, E. , Luengo, E. , Clement, C. C. , Choi, S. J. , Krogan, N. J. , Mosharov, E. V. , Santambrogio, L. , Grueninger, F. , Collin, L. , … Cuervo, A. M. (2021). Chaperone‐mediated autophagy prevents collapse of the neuronal metastable proteome. Cell, 184(10), 2696–2714.e25. 10.1016/j.cell.2021.03.048 33891876 PMC8152331

[acel13713-bib-0004] Buratta, S. , Tancini, B. , Sagini, K. , Delo, F. , Chiaradia, E. , Urbanelli, L. , & Emiliani, C. (2020). Lysosomal exocytosis, exosome release and secretory autophagy: The autophagic‐ and endo‐lysosomal systems go extracellular. International Journal of Molecular Sciences, 21(7), 2576–2596. 10.3390/ijms21072576 PMC7178086 32276321

[acel13713-bib-0005] Caballero, B. , Bourdenx, M. , Luengo, E. , Diaz, A. , Sohn, P. D. , Chen, X. , Wang, C. , Juste, Y. R. , Wegmann, S. , Patel, B. , Young, Z. T. , Kuo, S. Y. , Rodriguez‐Navarro, J. A. , Shao, H. , Lopez, M. G. , Karch, C. M. , Goate, A. M. , Gestwicki, J. E. , Hyman, B. T. , … Cuervo, A. M. (2021). Acetylated tau inhibits chaperone‐mediated autophagy and promotes tau pathology propagation in mice. Nature Communications, 12(1), 2238. 10.1038/s41467-021-22501-9 PMC8047017 33854069

[acel13713-bib-0006] Caballero, B. , Wang, Y. , Diaz, A. , Tasset, I. , Juste, Y. R. , Stiller, B. , Mandelkow, E. M. , Mandelkow, E. , & Cuervo, A. M. (2018). Interplay of pathogenic forms of human tau with different autophagic pathways. Aging Cell, 17(1), e12692. 10.1111/acel.12692 PMC5770880 29024336

[acel13713-bib-0007] Cannizzo, E. S. , Clement, C. C. , Morozova, K. , Valdor, R. , Kaushik, S. , Almeida, L. N. , Follo, C. , Sahu, R. , Cuervo, A. M. , Macian, F. , & Santambrogio, L. (2012). Age‐related oxidative stress compromises endosomal proteostasis. Cell Reports, 2(1), 136–149. 10.1016/j.celrep.2012.06.005 22840404 PMC3408590

[acel13713-bib-0008] Chacon‐Heszele, M. F. , Choi, S. Y. , Zuo, X. , Baek, J.‐I. , Ward, C. , & Lipschutz, J. H. (2014). The exocyst and regulatory GTPases in urinary exosomes. Physiological Reports, 2(8), e12116. 10.14814/phy2.12116 25138791 PMC4246586

[acel13713-bib-0009] Ciak, J. , & Hahn, F. E. (1966). Chloroquine: Mode of action. Science, 151(3708), 347–349. 10.1126/science.151.3708.347 4955293

[acel13713-bib-0010] Ciryam, P. , Tartaglia, G. G. , Morimoto, R. I. , Dobson, C. M. , & Vendruscolo, M. (2013). Widespread aggregation and neurodegenerative diseases are associated with supersaturated proteins. Cell Reports, 5(3), 781–790. 10.1016/j.celrep.2013.09.043 24183671 PMC3883113

[acel13713-bib-0011] Coppé, J. P. , Patil, C. K. , Rodier, F. , Sun, Y. , Muñoz, D. P. , Goldstein, J. , Nelson, P. S. , Desprez, P. Y. , & Campisi, J. (2008). Senescence‐associated secretory phenotypes reveal cell‐nonautonomous functions of oncogenic RAS and the p53 tumor suppressor. PLoS Biology, 6(12), 2853–2868. 10.1371/journal.pbio.0060301 19053174 PMC2592359

[acel13713-bib-0012] Cuervo, A. M. , & Dice, J. F. (2000). Age‐related decline in chaperone‐mediated autophagy. The Journal of Biological Chemistry, 275(40), 31505–31513. 10.1074/jbc.M002102200 10806201

[acel13713-bib-0013] Ferreira, J. V. , Rosa Soares, A. , Ramalho, J. S. , Ribeiro‐Rodrigues, T. , Máximo, C. , Zuzarte, M. , Girão, H. , & Pereira, P. (2019). Exosomes and STUB1/CHIP cooperate to maintain intracellular proteostasis. PLoS One, 14(10), e0223790. 10.1371/journal.pone.0223790 31613922 PMC6794069

[acel13713-bib-0014] Galluzzi, L. , Baehrecke, E. H. , Ballabio, A. , Boya, P. , Bravo‐San Pedro, J. M. , Cecconi, F. , Choi, A. M. , Chu, C. T. , Codogno, P. , Colombo, M. I. , Cuervo, A. M. , Debnath, J. , Deretic, V. , Dikic, I. , Eskelinen, E. L. , Fimia, G. M. , Fulda, S. , Gewirtz, D. A. , Green, D. R. , … Kroemer, G. (2017). Molecular definitions of autophagy and related processes. The EMBO Journal, 36(13), 1811–1836. 10.15252/embj.201796697 28596378 PMC5494474

[acel13713-bib-0015] Guo, W. , Grant, A. , & Novick, P. (1999). Exo84p is an exocyst protein essential for secretion. The Journal of Biological Chemistry, 274(33), 23558–23564. 10.1074/jbc.274.33.23558 10438536

[acel13713-bib-0016] Hessvik, N. P. , & Llorente, A. (2018). Current knowledge on exosome biogenesis and release. Cellular and Molecular Life Sciences, 75(2), 193–208. 10.1007/s00018-017-2595-9 28733901 PMC5756260

[acel13713-bib-0017] Horlbeck, M. A. , Gilbert, L. A. , Villalta, J. E. , Adamson, B. , Pak, R. A. , Chen, Y. , Fields, A. P. , Park, C. Y. , Corn, J. E. , Kampmann, M. , & Weissman, J. S. (2016). Compact and highly active next‐generation libraries for CRISPR‐mediated gene repression and activation. eLife, 5, e19760. 10.7554/eLife.19760 27661255 PMC5094855

[acel13713-bib-0018] Jassal, B. , Matthews, L. , Viteri, G. , Gong, C. , Lorente, P. , Fabregat, A. , Sidiropoulos, K. , Cook, J. , Gillespie, M. , Haw, R. , Loney, F. , May, B. , Milacic, M. , Rothfels, K. , Sevilla, C. , Shamovsky, V. , Shorser, S. , Varusai, T. , Weiser, J. , … D'Eustachio, P. (2020). The reactome pathway knowledgebase. Nucleic Acids Research, 48(D1), D498–D503. 10.1093/nar/gkz1031 31691815 PMC7145712

[acel13713-bib-0019] Johannes, L. (2021). The cellular and chemical biology of endocytic trafficking and intracellular delivery‐the GL‐Lect hypothesis. Molecules, 26(11), 3299–3314. 10.3390/molecules26113299 34072622 PMC8198588

[acel13713-bib-0020] Kaushik, S. , & Cuervo, A. M. (2015). Proteostasis and aging. Nature Medicine, 21(12), 1406–1415. 10.1038/nm.4001 26646497

[acel13713-bib-0021] Kaushik, S. , & Cuervo, A. M. (2018). The coming of age of chaperone‐mediated autophagy. Nature Reviews. Molecular Cell Biology, 19(6), 365–381. 10.1038/s41580-018-0001-6 29626215 PMC6399518

[acel13713-bib-0022] Kirchner, P. , Bourdenx, M. , Madrigal‐Matute, J. , Tiano, S. , Diaz, A. , Bartholdy, B. A. , Will, B. , & Cuervo, A. M. (2019). Proteome‐wide analysis of chaperone‐mediated autophagy targeting motifs. PLoS Biology, 17(5), e3000301. 10.1371/journal.pbio.3000301 31150375 PMC6561683

[acel13713-bib-0023] Krause, G. J. , & Cuervo, A. M. (2021). Assessment of mammalian endosomal microautophagy. Methods in Cell Biology, 164, 167–185. 10.1016/bs.mcb.2020.10.009 34225914 PMC8826487

[acel13713-bib-0024] Krause, G. J. , Kirchner, P. , Stiller, B. , Morozova, K. , Diaz, A. , Chen, K.‐H. , & Cuervo, A. M. (2022). Molecular determinants of the crosstalk between endosomal microautophagy and chaperone‐mediated autophagy. Cell Reports. (under revision) 10.1016/j.celrep.2023.113529 PMC10807933 38060380

[acel13713-bib-0025] Lopez‐Otin, C. , Blasco, M. A. , Partridge, L. , Serrano, M. , & Kroemer, G. (2013). The hallmarks of aging. Cell, 153(6), 1194–1217. 10.1016/j.cell.2013.05.039 23746838 PMC3836174

[acel13713-bib-0026] Mejlvang, J. , Olsvik, H. , Svenning, S. , Bruun, J. A. , Abudu, Y. P. , Larsen, K. B. , Brech, A. , Hansen, T. E. , Brenne, H. , Hansen, T. , Stenmark, H. , & Johansen, T. (2018). Starvation induces rapid degradation of selective autophagy receptors by endosomal microautophagy. The Journal of Cell Biology, 217(10), 3640–3655. 10.1083/jcb.201711002 30018090 PMC6168274

[acel13713-bib-0027] Miranda, A. M. , & Di Paolo, G. (2018). Endolysosomal dysfunction and exosome secretion: Implications for neurodegenerative disorders. Cell Stress, 2(5), 115–118. 10.15698/cst2018.05.136 31225476 PMC6551703

[acel13713-bib-0028] Monteiro, P. , Rossé, C. , Castro‐Castro, A. , Irondelle, M. , Lagoutte, E. , Paul‐Gilloteaux, P. , Desnos, C. , Formstecher, E. , Darchen, F. , Perrais, D. , Gautreau, A. , Hertzog, M. , & Chavrier, P. (2013). Endosomal WASH and exocyst complexes control exocytosis of MT1‐MMP at invadopodia. The Journal of Cell Biology, 203(6), 1063–1079. 10.1083/jcb.201306162 24344185 PMC3871436

[acel13713-bib-0029] Morozova, K. , Clement, C. C. , Kaushik, S. , Stiller, B. , Arias, E. , Ahmad, A. , Rauch, J. N. , Chatterjee, V. , Melis, C. , Scharf, B. , Gestwicki, J. E. , Cuervo, A. M. , Zuiderweg, E. R. , & Santambrogio, L. (2016). Structural and biological interaction of hsc‐70 protein with phosphatidylserine in endosomal microautophagy. The Journal of Biological Chemistry, 291(35), 18096–18106. 10.1074/jbc.M116.736744 27405763 PMC5000059

[acel13713-bib-0030] Moskalenko, S. , Tong, C. , Rosse, C. , Mirey, G. , Formstecher, E. , Daviet, L. , Camonis, J. , & White, M. A. (2003). Ral GTPases regulate exocyst assembly through dual subunit interactions. The Journal of Biological Chemistry, 278(51), 51743–51748. 10.1074/jbc.M308702200 14525976

[acel13713-bib-0031] Mukherjee, A. , Patel, B. , Koga, H. , Cuervo, A. M. , & Jenny, A. (2016). Selective endosomal microautophagy is starvation‐inducible in drosophila. Autophagy, 12(11), 1984–1999. 10.1080/15548627.2016.1208887 27487474 PMC5103356

[acel13713-bib-0032] Müller, M. , Schmidt, O. , Angelova, M. , Faserl, K. , Weys, S. , Kremser, L. , Pfaffenwimmer, T. , Dalik, T. , Kraft, C. , Trajanoski, Z. , Lindner, H. , & Teis, D. (2015). The coordinated action of the MVB pathway and autophagy ensures cell survival during starvation. eLife, 4, e07736. 10.7554/eLife.07736 25902403 PMC4424281

[acel13713-bib-0033] Nixon, R. A. (2020). The aging lysosome: An essential catalyst for late‐onset neurodegenerative diseases. Biochimica et Biophysica Acta ‐ Proteins & Proteomics, 1868(9), 140443. 10.1016/j.bbapap.2020.140443 32416272 PMC7388076

[acel13713-bib-0034] Piper, R. C. , & Katzmann, D. J. (2007). Biogenesis and function of multivesicular bodies. Annual Review of Cell and Developmental Biology, 23, 519–547. 10.1146/annurev.cellbio.23.090506.123319 PMC2911632 17506697

[acel13713-bib-0035] Ripley, C. R. , Fant, J. , & Bienkowski, R. S. (1993). Brefeldin A inhibits degradation as well as production and secretion of collagen in human lung fibroblasts. The Journal of Biological Chemistry, 268(5), 3677–3682. 8429043

[acel13713-bib-0036] Sahu, R. , Kaushik, S. , Clement, C. C. , Cannizzo, E. S. , Scharf, B. , Follenzi, A. , Potolicchio, I. , Nieves, E. , Cuervo, A. M. , & Santambrogio, L. (2011). Microautophagy of cytosolic proteins by late endosomes. Developmental Cell, 20(1), 131–139. 10.1016/j.devcel.2010.12.003 21238931 PMC3025279

[acel13713-bib-0037] Sala, A. J. , Bott, L. C. , & Morimoto, R. I. (2017). Shaping proteostasis at the cellular, tissue, and organismal level. The Journal of Cell Biology, 216(5), 1231–1241. 10.1083/jcb.201612111 28400444 PMC5412572

[acel13713-bib-0038] Schneider, J. L. , Suh, Y. , & Cuervo, A. M. (2014). Deficient chaperone‐mediated autophagy in liver leads to metabolic dysregulation. Cell Metabolism, 20(3), 417–432. 10.1016/j.cmet.2014.06.009 25043815 PMC4156578

[acel13713-bib-0039] Schuck, S. (2020). Microautophagy ‐ distinct molecular mechanisms handle cargoes of many sizes. Journal of Cell Science, 133(17), 1‐9. 10.1242/jcs.246322 32907930

[acel13713-bib-0040] Shields, S. B. , & Piper, R. C. (2011). How ubiquitin functions with ESCRTs. Traffic, 12(10), 1306–1317. 10.1111/j.1600-0854.2011.01242.x 21722280 PMC3171646

[acel13713-bib-0041] Simm, A. (2013). Protein glycation during aging and in cardiovascular disease. Journal of Proteomics, 92, 248–259. 10.1016/j.jprot.2013.05.012 23702329

[acel13713-bib-0042] Solvik, T. A. , Nguyen, T. A. , Tony Lin, Y. H. , Marsh, T. , Huang, E. J. , Wiita, A. P. , Debnath, J. , & Leidal, A. M. (2022). Secretory autophagy maintains proteostasis upon lysosome inhibition. The Journal of Cell Biology, 221(6), e202110151–e202110170. 10.1083/jcb.202110151 35446347 PMC9036093

[acel13713-bib-0043] Stoorvogel, W. , Kleijmeer, M. J. , Geuze, H. J. , & Raposo, G. (2002). The biogenesis and functions of exosomes. Traffic, 3(5), 321–330. 10.1034/j.1600-0854.2002.30502.x 11967126

[acel13713-bib-0044] Szklarczyk, D. , Gable, A. L. , Lyon, D. , Junge, A. , Wyder, S. , Huerta‐Cepas, J. , Simonovic, M. , Doncheva, N. T. , Morris, J. H. , Bork, P. , Jensen, L. J. , & Mering, C. V. (2019). STRING v11: Protein‐protein association networks with increased coverage, supporting functional discovery in genome‐wide experimental datasets. Nucleic Acids Research, 47(D1), D607–D613. 10.1093/nar/gky1131 30476243 PMC6323986

[acel13713-bib-0045] Takakuwa, J. E. , Nitika , Knighton, L. E. , & Truman, A. W. (2019). Oligomerization of Hsp70: Current perspectives on regulation and function. Frontiers in Molecular Biosciences, 6, 81‐88. 10.3389/fmolb.2019.00081 31555664 PMC6742908

[acel13713-bib-0046] TerBush, D. R. , Maurice, T. , Roth, D. , & Novick, P. (1996). The exocyst is a multiprotein complex required for exocytosis in *Saccharomyces cerevisiae* . The EMBO Journal, 15(23), 6483–6494. 8978675 PMC452473

[acel13713-bib-0047] Varela, I. , Cadiñanos, J. , Pendás, A. M. , Gutiérrez‐Fernández, A. , Folgueras, A. R. , Sánchez, L. M. , Zhou, Z. , Rodríguez, F. J. , Stewart, C. L. , Vega, J. A. , Tryggvason, K. , Freije, J. M. , & López‐Otín, C. (2005). Accelerated ageing in mice deficient in Zmpste24 protease is linked to p53 signalling activation. Nature, 437(7058), 564–568. 10.1038/nature04019 16079796

[acel13713-bib-0048] Wang, L. , Li, G. , & Sugita, S. (2004). RalA‐exocyst interaction mediates GTP‐dependent exocytosis. The Journal of Biological Chemistry, 279(19), 19875–19881. 10.1074/jbc.M400522200 14978027

[acel13713-bib-0049] Xu, J. , Yang, K. C. , Go, N. E. , Colborne, S. , Ho, C. J. , Hosseini‐Beheshti, E. , Lystad, A. H. , Simonsen, A. , Guns, E. T. , Morin, G. B. , & Gorski, S. M. (2022). Chloroquine treatment induces secretion of autophagy‐related proteins and inclusion of Atg8‐family proteins in distinct extracellular vesicle populations. Autophagy, 1‐14, 1–14. 10.1080/15548627.2022.2039535 PMC9629075 35220892

[acel13713-bib-0050] Yan, C. , & Theodorescu, D. (2018). RAL GTPases: Biology and potential as therapeutic targets in cancer. Pharmacological Reviews, 70(1), 1–11. 10.1124/pr.117.014415 29196555 PMC5712631

[acel13713-bib-0051] Yu, A. , Shibata, Y. , Shah, B. , Calamini, B. , Lo, D. C. , & Morimoto, R. I. (2014). Protein aggregation can inhibit clathrin‐mediated endocytosis by chaperone competition. Proceedings of the National Academy of Sciences, 111(15), E1481–E1490. 10.1073/pnas.1321811111 PMC3992642 24706768

[acel13713-bib-0052] Zago, G. , Biondini, M. , Camonis, J. , & Parrini, M. C. (2019). A family affair: A Ral‐exocyst‐centered network links Ras, Rac, rho signaling to control cell migration. Small GTPases, 10(5), 323–330. 10.1080/21541248.2017.1310649 28498728 PMC6748358

[acel13713-bib-0053] Zhang, C. , Brown, M. Q. , van de Ven, W. , Zhang, Z. M. , Wu, B. , Young, M. C. , Synek, L. , Borchardt, D. , Harrison, R. , Pan, S. , Luo, N. , Huang, Y. M. , Ghang, Y. J. , Ung, N. , Li, R. , Isley, J. , Morikis, D. , Song, J. , Guo, W. , … Raikhel, N. V. (2016). Endosidin2 targets conserved exocyst complex subunit EXO70 to inhibit exocytosis. Proceedings of the National Academy of Sciences of the United States of America, 113(1), E41–E50. 10.1073/pnas.1521248112 26607451 PMC4711834

