# Supplementary material for: Reduced endosomal microautophagy activity in aging associates with enhanced exocyst‐mediated protein secretion
Source: Aging Cell. 2022 Sep 18;21(10):e13713. doi: 10.1111/acel.13713 (PMC9577956; doi:10.1111/acel.13713)
Supplement: Supplementary file 1 — Appendix S1 [file ACEL-21-e13713-s001.docx]

Reduced endosomal microautophagy activity in aging associates with enhanced exocyst-mediated protein secretion

Gregory J. Krause^1,2^, Antonio Diaz^1,2^, Maryam Jafari^1,2^, Rabia R. Khawaja^1,2^, Esperanza Agullo-Pascual^3^, Olaya Santiago-Fernández^1,2^, Alicia L. Richards^4,5,6^, Kuei-Ho Chen^4,5,6^, Phillip Dmitriev^1,2^, Yan Sun^7^, Stephanie K. See^8^, Kotb Abdelmohsen^9^, Krystyna Mazan-Mamczarz^9^, Nevan J. Krogan^4,5,6^, Myriam Gorospe^9^, Danielle L. Swaney^4,5,6^, Simone Sidoli^7^, Jose Javier Bravo-Cordero^10,11^, Martin Kampmann^8*^, Ana Maria Cuervo^1,2*^

**Supplementary Material**

**Extended Experimental Procedures**

**References**

**Supplementary Figures S1 – S8**

**Supplementary Tables ST1-ST3**

**Uncropped blots**

**Raw data and statistical analysis** (please see Excel File)

**Extended Experimental Procedures**

***Animal Models***

Adult (3-6 months) male Wistar rats (Charles River Laboratories) and male young (4-6 months) and old (22-24 months old) mice (NIA colony), were used in this study. Mice with systemic knockout for LAMP-2A (L2A^-/-^) were generated by insemination of a wild-type female with spermatozoids from LAMP-2A^flox/flox^ (Schneider, Suh, & Cuervo, 2014) with L2A floxed to excise the gene in all tissues in the offspring (Bourdenx et al., 2021). All mice were genotyped at weaning and genotyping was re-confirmed post-mortem to correct for possible misplacement during husbandry. All mice were in the C57BL/6J background. Animals were maintained under specific pathogen-free conditions in ventilated cages with no more than 5 mice or 2 rats per cage. Only males were used in this study due to the difficulty in achieving meaningful sample size in both males and females in experiments utilizing isolated subcellular compartments and the current quota limits to access animals from the NIA colony. Animals were maintained at 19-23°C in a 12h light/dark cycle and fed *ad libitum,* except for starvation experiments when food was removed but they were given *ad libitum* access to water. To study LE/MVB protein degradation *in vivo*, rodents were injected i.p. with a single dose of leupeptin (Sigma L5793; 40mg/kg b.w. in saline (0.9% NaCl)) or with saline only and tissues were collected 4h after injection. Animals were randomly assigned to the leupeptin or saline injection group. Experiments using the chemical CMA activator (CA77.1) were performed as before (Bourdenx et al., 2021). Briefly, animals were randomly assigned to groups where they were fed a sucralose jelly pellet without (vehicle) or with CA77.1 at a daily dose of 30mg/kg b.w.. To prepare jelly pellets, the needed amount of CA77.1 per day was dissolved in ethanol and then mixed with a warm gelatin solution (100mg/ml, 10mg/ml sucralose in water) and aliquoted into a 24-well plate for solidification. During jelly pellet administration, animals were separated by the placement of a grid spacer in the same cage where they were housed to eliminate stress and competition while eating the pellets and to ensure each animal received a single pellet. Pellet consumption was monitored and the grid spacer was removed (average time 2 min). Animals were fed with jelly pellets for 5 consecutive days a week over the course of 5 months (18 months to 23 months of age). All animal breeding, handling, genotyping, and treatments in this study were done according to protocol and all animal studies were under an animal study protocol approved by the Institutional Animal Care and Use Committee of the Albert Einstein College of Medicine.

***Cell culture and treatments***

*NIH3T3 mouse fibroblasts* from the American Type Culture Collection (ATCC) were maintained in DMEM (Sigma-Aldrich) in the presence of 10% newborn calf serum (NCS) (Atlanta Biologicals) and 1% penicillin/streptomycin/fungizone (Invitrogen) in a 37°C incubator with 5% CO_2_. For eMI flux assays, cells were treated with lysosomal inhibitors ammonium chloride (20mM, American Bioanalytical) and leupeptin (100µM, Fisher Scientific) for the indicated times. To chemically inhibit the exocyst complex, cells were treated with the indicated increasing doses of Endosidin2 (Zhang et al., 2016) (Sigma, SML1681). Where indicated, experiments were performed in DMEM in the presence or absence of serum after several washes with Hanks Balanced Salt Solution. Cells knocked-down for the indicated proteins were transduced with lentivirus containing shRNAs listed in Table **S1**. Cell lines were routinely tested for mycoplasma contamination using a DNA staining protocol with Hoechst 33342 (Invitrogen, H3570) weekly and with MycoSenser PCR Assay Kit (Agilent Technologies) monthly. *Mouse primary ear fibroblasts* from 4 m and 22 m old mice were isolated as described previously (Varela et al., 2005). Briefly, after ears were removed from euthanized mouse, ears were digested in 4mg/ml collagenase (Sigma, C0130) and 4mg/ml dispase (Sigma, D4693) for 45 minutes before supplementation with additional DMEM and incubation overnight. Digested solution was put through a 45µm cell strainer to separate individual cells. Cells were maintained in DMEM media supplemented with 15% FBS (Corning, 35010CV), 1x non-essential amino acids (Invitrogen, 11140-050), 10mM HEPES buffer (Gibco, 15630080), 1mM sodium pyruvate (Corning, 25-000-CI), 66µM 2-mercaptoethanol (Fisher, 21985023), 2x anti-biotic/anti-mycotic solution (Gibco, 15240062) in a humidified 37°C, 5% CO_2_ incubator. Senescence was monitored using β-galactosidase staining and cells were discarded when percentage of senescent cells was >1%. For the isolation of extracellular vesicles, cells were cultured in media supplemented with 15% FBS that had exosomes removed by centrifugation for 18h at 110000xg at 4°C, with recovered supernatant filtered through a 0.2µm pore, as described previously (Shelke, Lasser, Gho, & Lotvall, 2014).

***Chemicals***

Chemicals were from the following sources: Percoll (GE Healthcare), recombinant tau 441 protein (rpeptide), recombinant α-synuclein protein (rpeptide), recombinant Cyclophilin A protein (R&D Systems), Endosidin2 (Sigma Aldrich), Brefeldin A (Sigma Aldrich), metrizamide (Cedarlane Laboratories), trypsin (Sigma Aldrich), PNGase F (Promega), Endo H (Promega), LysoSensor Yellow/Blue DND-160 (Invitrogen). Synthesis of the CMA activating compound was prepared as described previously (Bourdenx et al., 2021).

**A*ntibodies***

The dilutions and sources of antibodies used for immunoblot (IB), immunoprecipitation (IP), and isolated LE/MVB immunofluorescence (LE-IF) in this study were as follows: (dilutions, commercial source, and catalog number indicated in parentheses): rabbit anti-aldolase (1:1000 (IB), Cell Signaling, 3188S), rabbit anti-Alix (1:500 (IB), 1µg (IP), Novus, NBP1-90201), mouse anti-α synuclein (211) (1:2000 (IB), Santa Cruz, sc-12767), rabbit anti-ATP6V1B (1:1000 (IB), Abcam, ab200839), sheep anti-Bag6 (1µg (IP), R&D Systems, AF6438), rabbit anti-Bag6 (1:1000 (IB), 1:100 (LE-IF), 1µg (IP), Abcam, ab137076), rabbit anti-cathepsin D (E179) (1:500 (IB), Cell Signaling, 69854), mouse anti-CD63 (EPR21151) (1:500 (IB), Abcam, ab217345), rabbit anti-CD9 (SA35-08) (1:500 (IB), Novus, NBP2-67310), rabbit anti-cyclophilin A (1:1000 (IB), Cell Signaling, 2175), rabbit anti-Exoc2 (1:1000 (IB), 1:100 (LE-IF), Novus, NBP1-83786), mouse anti-Exoc3 (1:1000 (IB), Novus, NBP1-97500), mouse anti-Exoc4 (2E12) (1:1000 (IB), 1:100 (LE-IF), 1µg (IP), Sigma, MABC570), rabbit anti-Exoc5 (1:1000 (IB), Genetex, orb323262), rabbit anti-Exoc6 (1:1000 (IB), 1µg (IP), Proteintech, 12723-1-AP), rabbit anti-Exoc7 (1:1000 (IB), 1:100 (LE-IF), Abcam, ab118792), rabbit anti-GAPDH (1:2000 (IB), Cell Signaling, 2118S), rabbit anti-GBA (1:1000 (IB), Sigma, G4171), rabbit anti-hexokinase (1:1000 (IB), Cell Signaling, 2024S), mouse IgM anti-Hsc70 (13D3) (1:3000 (IB) and 1:200 (LE-IF), Novus, nb120-2788), rat anti-LAMP-1 (1D4B) (1:3000 (IB) 1:200 (LE-IF), 1µg (IP), Hybridoma Bank, 1D4B), mouse-anti LAMP-1 (LyC16) (1:1000 (IB) Enzo, adi-vam-en001), rabbit anti-LAMP-2A (1:5000 (IB), ThermoFisher Scientific, 512200) rabbit anti-LC3 (1:1000 (IB), Cell Signaling, 2775), rabbit anti-LRRK2 (MJFF3) (1:1000 (IB), Abcam, ab133475), rabbit anti-mTOR (1:2000 (IB), Cell Signaling, 2972), mouse anti-p62 (1:1000 (IB), Abcam, ab56416), rabbit anti-Rab5 (Cell Signaling, 3547), rabbit anti-Rab7 (D95F2) (1:500 (IB), Cell Signaling, 9367), mouse anti-RalA (F-5) (1:200 (IB), 1:100 (LE-IF), 1µg (IP), Santa Cruz, sc-374462), mouse anti-RalB (C-8) (1:200 (IB), Santa Cruz, sc-390108), mouse anti-total Tau (DA9) (1:3000 (IB) and 1:200 (LE-IF), gift from Dr. Peter Davies), rabbit anti-Tsg101 (1:1000 (IB), 1µg (IP), Abcam, ab30871), mouse anti-Vps4 (E-8) (1:500 (IB) and 1:100 (LE-IF), Santa Cruz, sc-133122), rabbit anti-Hsp70 (1µg (IP), Enzo, ADI-SPA-757), rabbit normal IgG (1µg (IP), Cell Signaling, 2729S). All secondary antibodies for routine immunofluorescence were from ThermoFisher Scientific. Secondary antibodies for stimulated emission depletion microscopy were from the following sources: goat anti-mouse 594 ATTO (1:200, Rockland, 610-155-121), goat anti-rabbit 594 ATTO (1:200, Sigma, 77671-1ML-F), goat anti-mouse 647 ATTO (1:200, Sigma, 50185-1ML-F), and goat anti-rabbit 647N ATTO (1:200, Rockland, 611-156-122). All antibodies used were validated following the multiple dilution method and, where available, using cell lines or tissues from animals knock-out for the antigen.

***Isolation of subcellular compartments***

Rodent liver lysosomes and LE/MVBs were isolated after tissue homogenization and centrifugation in density gradients. For lysosomes, homogenates were subjected to differential centrifugation to obtain a light mitochondrial/lysosome fraction, which was subsequently ultracentrifuged in a discontinuous metrizamide density gradient (Wattiaux, Wattiaux-De Coninck, Ronveaux-Dupal, & Dubois, 1978). Lysosomes active or inactive for CMA were separated by differential centrifugation as described before (Cuervo, Dice, & Knecht, 1997). LE/MVBs were isolated from rodent livers through consecutive discontinuous density gradients of sucrose and Percoll (GE Healthcare) as described previously (Krause & Cuervo, 2021; Sahu et al., 2011). Endolysosomal compartment integrity after isolation was assessed by measuring enzymatic β-hexosaminidase activity released from the isolated organelles (Storrie & Madden, 1990). Isolations with greater than 10% membrane breakage were discarded from further analyses. Cytosol was obtained by centrifugation at 100000xg for one hour of the post-nuclear supernatant. Except for the functional studies of LE/MVBs, all fractions were supplemented with protease inhibitor (10mM leupeptin, 10mM 4-(2-aminoethyl) benzenesulfonyl fluoride hydrochloride, 1mM pepstatin, 100mM EDTA) and phosphatase inhibitor cocktails 2 (Sigma P5726) and 3 (Sigma P0044) prior to freezing.

***pH measurement in isolated LE/MVBs***

Isolated LE/MVBs were incubated in 1µM LysoSensor Yellow/Blue DND-160 (Invitrogen, L7545) diluted in 20mM MOPS, 0.3M sucrose, pH 7.3 (MOPS-Sucrose) at 37°C for 5 minutes before pelleting at 25,000xg for 5 minutes at 4°C and resuspended in MOPS-Sucrose. Fluorescence was measured in a microplate reader (Tecan Infinite 200 Pro) (485/530nm), kept at 37°C. To generate a calibration curve, LysoSensor Yellow/Blue DND-160 was diluted to 1µM in 25mM MES calibration buffer, pH 4-7.5, containing 125mM KCl and 25mM NaCl. Fluorescence was measured using a microplate reader (340/440nm and 380/530nm) at 37°C.

***Protein deglycosylation***

Deglycosylation was performed according to manufacturer’s recommendations. Briefly, for PNGase F (Promega, V4831) 20µg of isolated LE/MVBs were denatured in 0.35% SDS, 7mM DTT in water before treatment with PNGase F for one hour at 37°C in 0.5M sodium phosphate buffer (pH 7.5) supplemented with NP-40 to a final concentration of 1%. For Endo H (Promega, V4875), 20µg of isolated LE/MVBs were denatured in provided denaturing solution before deglycosylation for 18h at 37°C with Endo H supplemented with provided reaction buffer. After deglycosylation with both reagents, samples were processed for standard SDS-PAGE immunoblotting.

***eMI measurements in isolated organelles***

*LE/MVB binding and internalization/degradation* of substrate proteins were analyzed using a previously described *in vitro* system (Krause & Cuervo, 2021). Briefly, LE/MVBs were preincubated or not with a cocktail of protease inhibitors for 10 min on ice and then incubated with recombinant proteins (Tau (rPeptide, T-1001-2), Cyclophilin A (R&D systems, 3589-CA-100), or α-synuclein (rPeptide, S-1001-2)) in MOPS-Sucrose buffer (20mM MOPS, 0.3M sucrose, pH 7.3) at 37°C for 30 min. At the end of the incubation, LE/MVBs were pelleted by centrifugation, washed to remove unbound substrate and processed for SDS-PAGE and immunoblotting. Binding was calculated as the amount of protein associated with organelles untreated with protease inhibitors and internalization/degradation as the difference between proteins present in organelles treated with protease inhibitors after subtracting for the amount bound. Where indicated, LE/MVBs were pre-incubated with antibodies for 10 min at room temperature prior to adding substrate protein. *Proteolysis* in isolated LE/MVBs was assayed by incubating with a pool of radiolabeled proteins in 20mM MOPS, 0.3M sucrose, 1mM DTT and 5.4µM cysteine, pH 7.3. This assay was performed in intact LE/MVBs or while incubating with 0.1% Triton-X100 (BioRad) to rupture the LE/MVB membrane and release the luminal proteases.

***eMI measurements in cultured cells***

eMI activity was measured in cells stably transduced with lentivirus containing the KFERQ-Split Venus reporter (Caballero et al., 2018; Uytterhoeven et al., 2015). Cells were plated in glass-bottom 96-well plates and eMI flux was assessed with the addition or not of endolysosomal protease inhibitors (20mM ammonium chloride and 100μM leupeptin; +N/L in text). Atter 16h, cells were fixed with 4% PFA and imaged using high-content microscopy (Operetta system, Perkin Elmer) set to collect images from > 800 cells/condition. Images were quantified using the manufacturer’s software to detect fluorescent puncta, and changes in eMI activity were quantified as changes in the number of fluorescent puncta per cell. Nuclei were labeled with Hoechst.

***Metabolic labeling and protein secretion***

Metabolic labeling was performed with ^3^H-leucine (2 µCi/ml) for 48h at 37°C (Auteri, Okada, Bochaki, & Dice, 1983). After extensive washing, cells were kept in media containing an excess of unlabeled leucine (2.8mM) to prevent the reutilization of radiolabeled leucine, supplemented with or without 10% NCS. Aliquots of media taken at different times were precipitated with 20% tri-carboxylic acid (TCA) and radioactivity in the precipitated fraction was used to determine protein secretion. Data were normalized to volume remaining in well at time of collection and is expressed as fold change from a control condition. Total radioactivity incorporated into the cellular proteins was determined as the amount of acid-precipitable radioactivity in labeled cells immediately after washing. Where indicated, cells were incubated with N/L or 5µg/mL Brefeldin A (Sigma, B5936) for up to 12h.

***Isolation of Extracellular Vesicles***

Extracellular vesicles (ECVs) were isolated from media of cells cultured for 48h in full DMEM media. Media was collected and centrifuged sequentially at 300xg, 2000xg, and 10000xg to remove debris, followed by concentration using a 30000 molecular weight cut-off (MWCO) column (Sartorius, VS2021). ECVs were pelleted by ultracentrifugation at 110000xg for 3h at 4°C, followed by a second spin with the same settings for 1 hour after washing with PBS. ECVs were resuspended in PBS and prepared for standard SDS-PAGE immunoblotting as described below.

**Nano-Flow Cytometry**

The nFCM flow NanoAnalyzer was used to measure the concentration and size of particles following the manufacturer's instructions and as described previously (Arab et al., 2021). Briefly, two single photon-counting avalanche photodiodes (APDs) were used to detect individual particles' side scatter (SSC) simultaneously. The instrument was calibrated separately for concentration and size using 250 nm PE- and AF488 fluorophore-conjugated silica beads and a Silica Nanosphere Cocktail. PBS was used as a background signal which was removed from each sample. Samples were diluted to optimal concentration to collect 2000-12000 events per 1-minute run.

***Co-Immunoprecipitation***

Isolated LE/MVBs were resuspended in co-immunoprecipitation (coIP) buffer (20mM MOPS, 0.3M sucrose, 1mM DTT, 0.5% NP-40, pH 7.3), supplemented with a cocktail of protease inhibitors and incubated on ice for ten minutes before centrifugation at 16000xg for 15 minutes at 4°C to remove remaining intact membranes. Solubilized proteins were incubated with primary antibodies for 16h under continuous rotation. Protein A/G Plus agarose beads (Santa Cruz, sc-2003) were added for an additional 1h. After extensive washing, immunoprecipitated proteins were eluted and prepared for standard SDS-PAGE and immunoblotting.

***Protein topology and stability in isolated organelles***

*Trypsinization assay*: To assess the percentage of protein that is membrane-bound or internalized in the LE/MVB lumen, isolated organelles were incubated with trypsin (Sigma, Cat # T1426) in MOPS-Sucrose (20mM MOPS, 0.3M sucrose, pH 7.3) at room temperature for 15 minutes. Where, indicated, Triton-X100 (0.1%) was added to a control sample to lyse membrane and expose internalized proteins to trypsin degradation.

*Stability assay:* To assess the stability of a protein in the membrane, LE/MVBs were incubated at 37°C for time intervals up to 45 min in 20mM MOPS, 0.3M sucrose, 1mM DTT and 5.4µM cysteine pH 7.3, with a control condition incubated with protease inhibitors to assess for degradation as the cause for stability loss during incubation. Protein stability is determined in reference to unincubated organelles. After each time point, samples are prepared for standard SDS-PAGE immunoblotting.

***Protein electrophoresis and immunoblotting***

Protein concentration was determined using the Lowry method (Lowry, Rosebrough, Farr, & Randall, 1951) with bovine serum albumin as the standard. Immunoblotting was performed after transferring SDS-PAGE gels to nitrocellulose membrane and blocking with 5% milk in 0.01% Tween-TBS for 1h at room temperature (Towbin, Staehelin, & Gordon, 1979). The proteins of interest were visualized after incubation with primaries by chemiluminescence using horseradish peroxidase-conjugated secondary antibodies in the LAS-3000 Imaging System (Fujifilm, Tokyo, Japan). Densitometric quantification of the membranes was performed using ImageJ (NIH; (Schindelin et al., 2012)). All protein quantifications were done after normalization of protein levels to Ponceau S staining. When the number of experimental conditions exceeded the number of lanes available in the gel, all gels for the same experiment included one lane with the same sample, which was used to normalize samples across gels after densitometric quantification. *Blue-native electrophoresis:* LE/MVB membranes were solubilized in 1% octyl glycoside diluted in 20mM MOP, 0.3M sucrose, pH 7.3 for 15 min on ice followed by centrifugation at 16000xg for 15 min. The supernatant was prepared for blue-native electrophoresis with NativePAGE Sample Prep Kit (ThermoFisher, BN2008). Electrophoresis was done using NativePAGE 3-12% Novex Bis-Tris pre-cast gels (Invitrogen, bn1001) before transferring to a PVDF membrane (Immobilon).

***RNA isolation and qPCR analysis***

Total RNA was isolated using the RNeasy Plus kit (Qiagen) according to the manufacturer’s instructions. RNA was extracted from liver tissue after homogenization in Trizol (Invitrogen). Total RNA was reverse transcribed into cDNA using Superscript II (Invitrogen). Quantitative RT-PCR was performed using Podwer SYBR Green PCR mix (Applied Biosystems) on a StepOne Plus Real-Time PCR system (Applied Biosystems) using the primers shown in Table **S2**. Relative RNA abundance was calculated using the comparative 2^-ΔΔCt^ method (Livak & Schmittgen, 2001). Water was used as a negative control. All reactions were performed in triplicate.

***Immunofluorescence***

LE/MVB imaging was performed by spotting isolated LE/MVBs on a PTFE printed slide, 6mm diameter (Electron microscopy sciences) and fixing with 8% PFA for 15 minutes. Permeabilization was performed with 0.1% glycine, 2% fetal bovine serum, 1% BSA, with 0.01% Triton X-100, after which LE/MVBs were incubated with primary and secondary antibodies at the described concentrations for 10 min each at room temperature. Mounting was performed with ProLong Diamond Anti-Fade Mountant (ThermoFisher Scientific). Confocal images were acquired with a Leica TCS SP8 (Leica Microsystem) with a 63x objective and a 1.4 numerical aperture. All images were prepared using the LASX software (Leica Microsystem) and analyzed with ImageJ (NIH) software (Schindelin et al., 2012). The colocalization plugin in ImageJ was used to determine the percentage of co-localization after the thresholding of individual images.

***τSTED imaging***

Samples were imaged on a Leica TCS SP8 STED 3X outfitted with a *τ*STED module. The system was equipped with a White Light Laser and a STED pulsed laser 775nm, using a Leica HC Plan-Apo 100x/1.4 NA Oil lens. 1024x1024-pixel images were acquired at 200 Hz using HyD detectors. Samples were illuminated with the white light laser at 488, 594, and 647 nm wavelengths with the following parameters based on primary and secondary antibody staining: 1) *Alexa Fluor 488* acquisition parameters were: 6%-10% (excitation laser strength), 500-570nm (emission wavelength range), 454 (gain), line repetition of 3, frame repetition of 4. 2) *anti-mouse IgM Atto594* acquisition parameters were: 1.5-3% (excitation laser strength), 610-630nm (emission wavelength range), 364 (gain), line and frame repetition of 2, with the 775nm STED pulsed laser at 50%. *τ*STED parameters of 130 (Tau strength) and 0.3-10.9ns (time gating) were used. 3) *anti-mouse IgG Atto647 (Tau DA9 primary antibody)* acquisition parameters were: 8% (excitation laser strength), 659-749 nm (emission wavelength range), 418 (gain), line repetition of 3, frame repetition of 4, with the 775nm STED pulsed laser at 50%. *τ*STED parameters were 50 (*τ* strength) and 0.2-10.6ns (time gating). 4) *anti-rabbit IgG Atto647N (Tau Y9 primary antibody and Exoc2 primary antibody)* acquisition parameters were: 8% (excitation laser strength), 659-749 nm (emission wavelength range), 463 (gain), line and frame repetition of 4, with the 775nm STED pulsed laser at 50%. *τ*STED parameters were: 30 (*τ* strength) and 0.1-10ns (time gating). 5) *anti-rabbit IgG Atto647N (Bag6 primary antibody)* acquisition parameters were: 8-20% (excitation laser strength), 659-749 nm (emission wavelength range), 364 (gain), line and frame repetition of 2, with the 775nm STED pulsed laser at 60%. *τ* STED parameters were 100 (*τ* strength) and 0.2-11.1ns (time gating). 6) *anti-rabbit IgG Atto594 (Bag6 primary antibody)* acquisition parameters were: 20% (excitation laser strength), 610-630nm (emission wavelength range), 304 (gain), line and frame repetition of 4, with the 775nm STED pulsed laser at 50%. *τ* STED parameters were 10 (Tau strength) and 0.1-10.6ns (time gating).

***τ* *STED image analysis***

Images were prepared using the LASX software (Leica Microsystem) and analyzed with ImageJ (NIH) software (Schindelin et al., 2012). Briefly, images were saved as individual channels and a gaussian deblur of sigma=2 was applied. The percentage of total signal in the membrane and lumen for each channel was assessed by using the LAMP-1 channel to draw a region of interest (ROI) around the whole LE/MVB and the inner portion of the membrane to select the lumen. Both ROIs were saved and applied to the other channels in the same LE/MVB. Values were calculated as the percentage of total LE/MVB signal in each region (membrane or lumen). The distribution of the signal in the LE/MVB membrane was measured by using the plot profile function in the LAMP-1 channel to trace the membrane. This ROI was then saved and applied to all channels for a given LE/MVB. Signal for each channel was normalized independently to the average signal in that channel. Signal that was greater than 1.25x the average signal was defined as a “hot spot” for that protein the LE/MVB membrane. Overlap of hot spots in different channels was defined as two or more channels having signal >1.25x the average signal for those channels in the same location in the profile around the LE/MVB membrane. Values are expressed as the percentage of total hot spots for a given channel either in reference to the LE/MVB perimeter length or the number of total hot spots for a given channel.

***Quantitative Proteomics and Protein Pathway Analysis***

For the mass spectrometry analysis of LE/MVBs from 4 m and 22 m old mice*,* isolated LE/MVBs were denatured by the addition of 50uL of LYSE buffer solution from the PreOmics iST sample preparation kit, and further sample preparation was performed according to the iST kit protocol. Dried peptide samples were then resuspended in 0.1% formic acid, and approximately 500ng of digested peptides per sample were loaded onto a 75μm ID column packed with 15cm of BEH C18 1.7μm, 130Å particles (Waters) by an EASY-nLC 1200 (Thermo Fisher) high-pressure liquid chromatography system (interfaced to the mass spectrometer via a Nanospray Flex source (Thermo Fisher). Peptides were directly eluted into an Orbitrap Fusion Lumos Tribrid mass spectrometer (Thermo Fisher) operated in positive data-dependent acquisition mode over the course of a 80-minute acquisition by gradient elution from 0-22%B in from 0-56min, followed by an increase to 32%B from 56-63min, and then a ramp to 95%B to wash the column. The gradient was delivered by the EASY-nLC 1200 (Thermo Fisher), and the composition of mobile phase A and B were 0.1% formic acid in water and 0.1% formic acid in 80% acetonitrile, respectively.

MS parameter settings were set as follows: Fourier transform (FT) MS1 resolution (240,000 @ 200 m/z) with automatic gain control (AGC) target of 1e6, and maximum injection time (IT) of 50 ms. MS1 scan range was 350-1350 m/z. Advanced peak detection (APD) was enabled with a default charge state of 2. Dynamic exclusion was set to 40 seconds with a 10 ppm mass tolerance. Monoisotopic precursor selection (MIPS) was set to peptide mode, and charge states of 2-6 were selected for MS2 higher-energy collisional dissociation (HCD) fragmentation at 32% in the ion trap using rapid scans. Quadrupole isolation width was set to 0.7 m/z. MS2 scans were acquired over a range of 200-1200 m/z with an AGC target if 3e4 and maximum IT of 20 ms. MS2 cycle time was set at 1 second, where MS2 scans were acquired for a maximum time of 1 second before acquiring an MS1 scan.

All data were searched against the Uniprot mouse database (downloaded 7/2/2020). Peptide and protein identification searches were performed using the Andromeda search engine (Jürgen Cox et al., 2011) within MaxQuant (J. Cox et al., 2014). Search parameters included fixed modification settings for carbamidomethylation of cysteine residues variable modification settings for methionine oxidation and protein N-terminal acetylation, and a maximum of two missed tryptic cleavages. All peptide and protein identifications were filtered to a 1% false-discovery rate (FDR) at the peptide spectrum match (PSM) and protein level using the target-decoy strategy (Elias & Gygi, 2007). Label-free quantification and statistical testing was performed using the MSstats statistical R-package (Choi et al., 2014). The Benjamini-Hochberg protocol for false discovery rate or control of false discovery rate by using fold change cutoff (|fold change| > 1.25), as previously suggested (Pascovici, Handler, Wu, & Haynes, 2016) were used. Relevant protein lists were ranked according to fold change compared to Ctr mice and submitted to rank-rank hypergeometric overlap test in R (v. 3.6.2) (Team, 2018) using default settings (Plaisier, Taschereau, Wong, & Graeber, 2010).

Analysis of the presence of KFERQ-like motifs was performed as previously described (Kirchner et al., 2019) using the publicly available tool: <https://rshine.einsteinmed.org/>. For the proteomics studies with isolated LE/MVBs, the mass spectrometry data files (raw and search results) have been deposited to the ProteomeXchange Consortium (Deutsch et al., 2017) via the PRIDE partner repository with dataset identifier

**Glycan proteomics**

Proteins were resuspended in a buffer containing 5% SDS, 5 mM DTT and 50 mM ammonium bicarbonate (pH = 8), and left on the bench for 1 hour for disulfide bond reduction. Samples were then alkylated with 20 mM iodoacetamide in the dark for 30 minutes. Afterward, phosphoric acid was added to the sample at a final concentration of 1.2%. Samples were diluted in six volumes of binding buffer (90% methanol and 10 mM ammonium bicarbonate, pH 8.0). After gentle mixing, the protein solution was loaded to an S-trap filter (Protifi) and spun at 500 g for 30 sec. The sample was washed twice with binding buffer. Finally, 1 µg of sequencing grade trypsin (Promega), diluted in 50 mM ammonium bicarbonate, was added into the S-trap filter and samples were digested at 37^o^C for 18 h. Peptides were eluted in three steps: (i) 40 µl of 50 mM ammonium bicarbonate, (ii) 40 µl of 0.1% TFA and (iii) 40 µl of 60% acetonitrile and 0.1% TFA. The peptide solution was pooled, spun at 1,000 g for 30 sec and dried in a vacuum centrifuge. After elution, samples were resuspended in 10 µl of Glycoprotein Denaturing Buffer and 1 µl of PNGase F (New England Biolabs). The incubation occurred for 1 hour at 37^o^C.

Prior to mass spectrometry analysis, samples were desalted using a 96-well plate filter (Orochem) packed with 1 mg of Oasis HLB C-18 resin (Waters). Briefly, the samples were resuspended in 100 µl of 0.1% TFA and loaded onto the HLB resin, which was previously equilibrated using 100 µl of the same buffer. After washing with 100 µl of 0.1% TFA, the samples were eluted with a buffer containing 70 µl of 60% acetonitrile and 0.1% TFA and then dried in a vacuum centrifuge.

Samples were resuspended in 10 µl of 0.1% TFA and loaded onto a Dionex RSLC Ultimate 300 (Thermo Scientific), coupled online with an Orbitrap Fusion Lumos (Thermo Scientific). Chromatographic separation was performed with a two-column system, consisting of a C-18 trap cartridge (300 µm ID, 5 mm length) and a picofrit analytical column (75 µm ID, 25 cm length) packed in-house with reversed-phase Repro-Sil Pur C18-AQ 3 µm resin. To analyze the proteome, peptides were separated using a 120 min gradient from 4-30% buffer B (buffer A: 0.1% formic acid, buffer B: 80% acetonitrile + 0.1% formic acid) at a flow rate of 300 nl/min. The mass spectrometer was set to acquire spectra in a data-dependent acquisition (DDA) mode. Briefly, the full MS scan was set to 300-1200 m/z in the orbitrap with a resolution of 120,000 (at 200 m/z) and an AGC target of 5x10e5. MS/MS was performed in the ion trap using the top speed mode (2 secs), an AGC target of 1x10e4 and an HCD collision energy of 35. Data analysis was performed using the Protein Metrics software using the glycoproteomics node.

**CRISPRi screen and data analysis**

CRISPR interference (CRISPRi) screen was performed in human K562 myelogenous leukemia cells using an sgRNA library targeting 1176 genes related to proteostasis pathways as described in (Horlbeck et al., 2016). The sgRNA library consists of 5 sgRNAs targeting each gene and a pool of nontargeting control sgRNAs. K562 cells stably expressing a nuclease-dead form of Cas9 fused to the transcriptional repressor KRAB (dCas9-KRAB) and the KFERQ-Split Venus fluorescent eMI reporter were transfected at a low multiplicity of infection with the sgRNA library to ensure infection with one sgRNA per cell. After 3 days of selection with puromycin, fluorescent activated cell sorting (FACS; FACS Aria II (BD Bioscience)) was used to separate cells with high (top 33%) and low (bottom 33%) Split Venus signal and their genomic DNA was extracted (NucleoSpin Blood XL, Machery Nagel). Extracted DNA was used as a PCR template to amplify sgRNA sequence (Q5 polymerase, New England Biolabs), after which it was size-selected for the sample size using SPRIselect beads (Beckman Coulter). Purified PCR sample was quantified (Qubit 4 fluorometer, ThermoFisher) before submitting for deep sequencing analysis (HiSeq4000, Illumina).

Data analysis for CRISPRi screen was performed as described in (Chen et al., 2019). Significance is calculated based on the distribution of sgRNA phenotypes using the Mann-Whitney *U* test. Selected hits were further analyzed for biochemical validation.

***Quantification, Statistical Analysis and Software***

All data presented are mean±s.e.m and individual values. Prior to statistical testing, normality was assessed using the Shapiro Wilk test. Statistical significance was compared by two-tailed unpaired Student’s t-test for two groups, one-way ANOVA for a single parameter in multiple groups or two-way ANOVA for multiple parameters in multiple groups. The post hoc test used for multiple comparisons is stated in the legend of the figures. The number of animals used per experiment was calculated through power analysis based on previous results. Statistical analyses were performed either in GraphPad Prism 9.0 or using Python (Python software foundation v.3.7.4 available at <https://www.python.org/>) and the scientific python stack: scipy (v.1.3.1) (Jones, Oliphant, & Peterson, 2001), numpy (v.1.17.2) (van der Walt, Colbert, & Varoquaux, 2011), and matplotlib (v.3.1.1) (Hunter, 2007). Hsc70 interaction predictions we done using the crystal structure information from the RCSB-Protein Data Bank (<https://www.rcsb.org/>): 3HSC Structure of the ATPase fragment of Hsc70, 4H5T Structure of Hsc70 nucleotide binding site with ADP and Mg. 1KAY Structure of Hsc70 ATPase domain K71A mutant, 3FZF Structure of Hsc70/Bag1 in complex with ATP; 1HX1 Structure of a Bag domain in complex with the Hsc70 ATPase domain. Pathway analysis was performed using the STRING database (<https://string-db.org/>) (Szklarczyk et al., 2019) and Reactome 2022 (https://reactome.org/) (Gillespie et al., 2022). Schematics for this paper, including the graphical abstract and the following figure panels: 1a, 1g, 3j, 4a, 5a, 6f, and 7f were generated with BioRender (© BioRender 2022).

**References Extended Experimental Methods**

Arab, T., Mallick, E. R., Huang, Y., Dong, L., Liao, Z., Zhao, Z., . . . Witwer, K. W. (2021). Characterization of extracellular vesicles and synthetic nanoparticles with four orthogonal single-particle analysis platforms. *J Extracell Vesicles, 10*(6), e12079. doi:10.1002/jev2.12079

Auteri, J. S., Okada, A., Bochaki, V., & Dice, J. F. (1983). Regulation of intracellular protein degradation in IMR- 90 human diploid fibroblasts. *J Cell Physiol, 115*, 159-166.

Bourdenx, M., Martin-Segura, A., Scrivo, A., Rodriguez-Navarro, J. A., Kaushik, S., Tasset, I., . . . Cuervo, A. M. (2021). Chaperone-mediated autophagy prevents collapse of the neuronal metastable proteome. *Cell, 184*(10), 2696-2714 e2625. doi:10.1016/j.cell.2021.03.048

Caballero, B., Wang, Y., Diaz, A., Tasset, I., Juste, Y. R., Stiller, B., . . . Cuervo, A. M. (2018). Interplay of pathogenic forms of human tau with different autophagic pathways. *Aging Cell, 17*(1). doi:10.1111/acel.12692

Chen, J. J., Nathaniel, D. L., Raghavan, P., Nelson, M., Tian, R., Tse, E., . . . Kampmann, M. (2019). Compromised function of the ESCRT pathway promotes endolysosomal escape of tau seeds and propagation of tau aggregation. *J Biol Chem, 294*(50), 18952-18966. doi:10.1074/jbc.RA119.009432

Choi, M., Chang, C. Y., Clough, T., Broudy, D., Killeen, T., MacLean, B., & Vitek, O. (2014). MSstats: an R package for statistical analysis of quantitative mass spectrometry-based proteomic experiments. *Bioinformatics, 30*(17), 2524-2526. doi:10.1093/bioinformatics/btu305

Cox, J., Hein, M. Y., Luber, C. A., Paron, I., Nagaraj, N., & Mann, M. (2014). Accurate proteome-wide label-free quantification by delayed normalization and maximal peptide ratio extraction, termed MaxLFQ. *Mol Cell Proteomics, 13*(9), 2513-2526. doi:10.1074/mcp.M113.031591

Cox, J., Neuhauser, N., Michalski, A., Scheltema, R. A., Olsen, J. V., & Mann, M. (2011). Andromeda: A Peptide Search Engine Integrated into the MaxQuant Environment. *Journal of Proteome Research, 10*(4), 1794-1805. doi:10.1021/pr101065j

Cuervo, A. M., Dice, J. F., & Knecht, E. (1997). A population of rat liver lysosomes responsible for the selective uptake and degradation of cytosolic proteins. *J Biol Chem, 272*(9), 5606-5615. doi:10.1074/jbc.272.9.5606

Deutsch, E. W., Csordas, A., Sun, Z., Jarnuczak, A., Perez-Riverol, Y., Ternent, T., . . . Vizcaino, J. A. (2017). The ProteomeXchange consortium in 2017: supporting the cultural change in proteomics public data deposition. *Nucleic Acids Res, 45*(D1), D1100-d1106. doi:10.1093/nar/gkw936

Elias, J. E., & Gygi, S. P. (2007). Target-decoy search strategy for increased confidence in large-scale protein identifications by mass spectrometry. *Nature Methods, 4*(3), 207-214. doi:10.1038/nmeth1019

Gillespie, M., Jassal, B., Stephan, R., Milacic, M., Rothfels, K., Senff-Ribeiro, A., . . . D'Eustachio, P. (2022). The reactome pathway knowledgebase 2022. *Nucleic Acids Res, 50*(D1), D687-d692. doi:10.1093/nar/gkab1028

Horlbeck, M. A., Gilbert, L. A., Villalta, J. E., Adamson, B., Pak, R. A., Chen, Y., . . . Weissman, J. S. (2016). Compact and highly active next-generation libraries for CRISPR-mediated gene repression and activation. *Elife, 5*. doi:10.7554/eLife.19760

Hunter, J. D. (2007). Matplotlib: A 2D graphics environment. Computing in Science and Engineering. *9*, 90-95.

Jones, E., Oliphant, T., & Peterson, P. (2001). SciPy: Open Source Scientific Tools for Python. .

Kirchner, P., Bourdenx, M., Madrigal-Matute, J., Tiano, S., Diaz, A., Bartholdy, B. A., . . . Cuervo, A. M. (2019). Proteome-wide analysis of chaperone-mediated autophagy targeting motifs. *PLoS Biol, 17*(5), e3000301. doi:10.1371/journal.pbio.3000301

Krause, G. J., & Cuervo, A. M. (2021). Assessment of mammalian endosomal microautophagy. *Methods Cell Biol, 164*, 167-185. doi:10.1016/bs.mcb.2020.10.009

Livak, K. J., & Schmittgen, T. D. (2001). Analysis of relative gene expression data using real-time quantitative PCR and the 2(-Delta Delta C(T)) Method. *Methods, 25*(4), 402-408. doi:10.1006/meth.2001.1262

Lowry, O. H., Rosebrough, N. J., Farr, A. L., & Randall, R. J. (1951). Protein measurement with the Folin phenol reagent. *J Biol Chem, 193*(1), 265-275.

Pascovici, D., Handler, D. C., Wu, J. X., & Haynes, P. A. (2016). Multiple testing corrections in quantitative proteomics: A useful but blunt tool. *Proteomics, 16*(18), 2448-2453. doi:10.1002/pmic.201600044

Plaisier, S. B., Taschereau, R., Wong, J. A., & Graeber, T. G. (2010). Rank-rank hypergeometric overlap: identification of statistically significant overlap between gene-expression signatures. *Nucleic Acids Res, 38*(17), e169. doi:10.1093/nar/gkq636

Sahu, R., Kaushik, S., Clement, C. C., Cannizzo, E. S., Scharf, B., Follenzi, A., . . . Santambrogio, L. (2011). Microautophagy of cytosolic proteins by late endosomes. *Dev Cell, 20*(1), 131-139. doi:10.1016/j.devcel.2010.12.003

Schindelin, J., Arganda-Carreras, I., Frise, E., Kaynig, V., Longair, M., Pietzsch, T., . . . Cardona, A. (2012). Fiji: an open-source platform for biological-image analysis. *Nat Methods, 9*(7), 676-682. doi:10.1038/nmeth.2019

Schneider, J. L., Suh, Y., & Cuervo, A. M. (2014). Deficient chaperone-mediated autophagy in liver leads to metabolic dysregulation. *Cell Metab, 20*(3), 417-432. doi:10.1016/j.cmet.2014.06.009

Shelke, G. V., Lasser, C., Gho, Y. S., & Lotvall, J. (2014). Importance of exosome depletion protocols to eliminate functional and RNA-containing extracellular vesicles from fetal bovine serum. *J Extracell Vesicles, 3*. doi:10.3402/jev.v3.24783

Storrie, B., & Madden, E. A. (1990). Isolation of subcellular organelles. *Methods Enzymol, 182*, 203-225. doi:10.1016/0076-6879(90)82018-w

Szklarczyk, D., Gable, A. L., Lyon, D., Junge, A., Wyder, S., Huerta-Cepas, J., . . . Mering, C. V. (2019). STRING v11: protein-protein association networks with increased coverage, supporting functional discovery in genome-wide experimental datasets. *Nucleic Acids Res, 47*(D1), D607-D613. doi:10.1093/nar/gky1131

Team, R. C. (2018). R: A language and environment for statistical computing. R Foundation for Statistical Computing,. Retrieved from <https://www.R-project.org/>

Towbin, H., Staehelin, T., & Gordon, J. (1979). Electrophoretic transfer of proteins from polyacrylamide to nitrocellulose sheets: procedure and some applications. *Proc Natl Acad Sci, 76*, 4350-4354.

Uytterhoeven, V., Lauwers, E., Maes, I., Miskiewicz, K., Melo, M. N., Swerts, J., . . . Verstreken, P. (2015). Hsc70-4 Deforms Membranes to Promote Synaptic Protein Turnover by Endosomal Microautophagy. *Neuron, 88*(4), 735-748. doi:10.1016/j.neuron.2015.10.012

van der Walt, S., Colbert, S. C., & Varoquaux, G. (2011). The NumPy Array: A Structure for Efficient Numerical Computation. *Computing in Science and Engineering, 13*, 22-30.

Varela, I., Cadiñanos, J., Pendás, A. M., Gutiérrez-Fernández, A., Folgueras, A. R., Sánchez, L. M., . . . López-Otín, C. (2005). Accelerated ageing in mice deficient in Zmpste24 protease is linked to p53 signalling activation. *Nature, 437*(7058), 564-568. doi:10.1038/nature04019

Wattiaux, R., Wattiaux-De Coninck, S., Ronveaux-Dupal, M., & Dubois, F. (1978). Isolation of rat liver lysosomes by isopycnic centrifugation in a metrizamide gradient. *J Cell Biol, 78*, 349-368.

Zhang, C., Brown, M. Q., van de Ven, W., Zhang, Z. M., Wu, B., Young, M. C., . . . Raikhel, N. V. (2016). Endosidin2 targets conserved exocyst complex subunit EXO70 to inhibit exocytosis. *Proc Natl Acad Sci U S A, 113*(1), E41-50. doi:10.1073/pnas.1521248112

**Supplementary Figures**

**
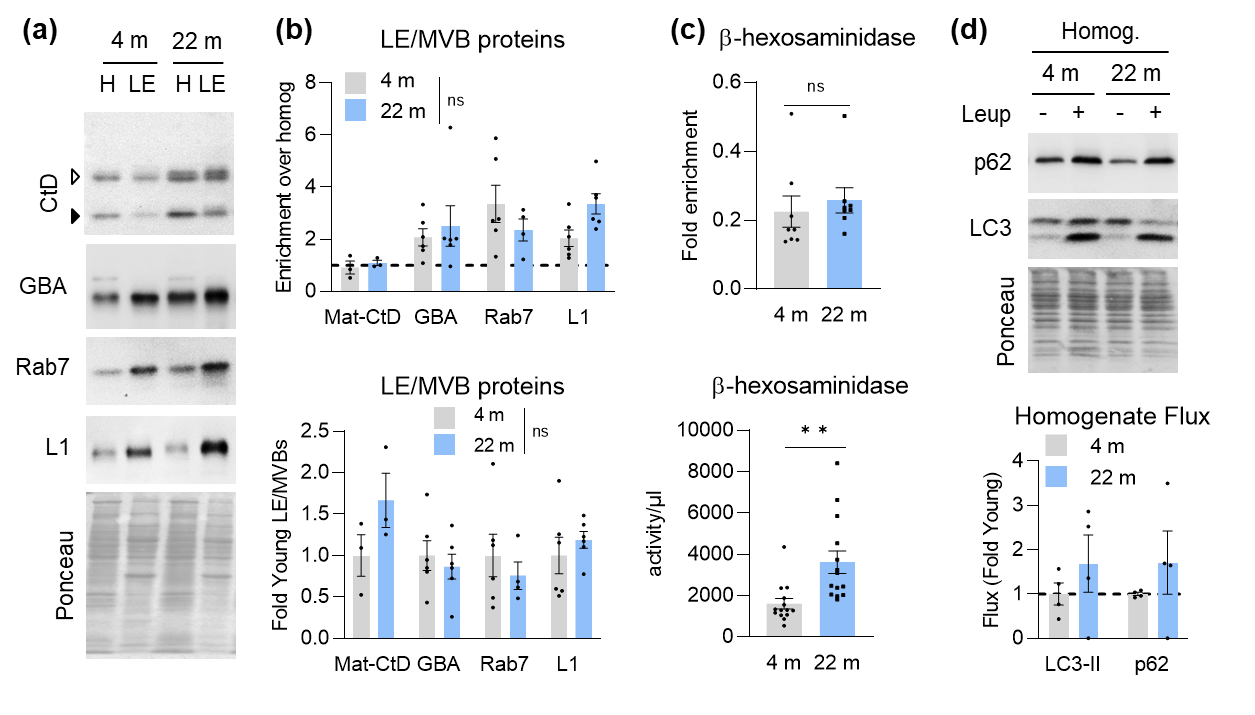
**

**Supplementary Figure 1. Characterization of LE/MVB compartments in aging.** (a,b) Representative immunoblots (a) and quantification (b) for luminal (Cathepsin D; CtD and GBA) and membrane proteins (Rab7 and LAMP-1; L1) in LE/MVBs isolated from 4 m and 22 m old mice. Quantification represents enrichment over homogenate (b; top) and fold change from 4m old LE/MVB average (b; bottom) n =3-6 mice. (c) β-hexosaminidase activity enrichment (top) and activity/µl (bottom) in LE/MVB isolated from 4 m and 22 m old mice. n =14 mice. (d) representative immunoblot (top) and quantification (bottom) for p62 and LC3 to demonstrate the efficacy of leupeptin (Leup) injections to block endolysosomal proteolysis in livers from 4 m and 22 m old mice in Figure 2. n=4 mice. All data are mean+s.e.m. and individual values. Ponceau staining is shown as loading control in the immunoblots. One sample multiple t-tests (b, d) and unpaired two-sided t-test (c) were used. Differences were significant for **p<0.01. ns: not significant.

**
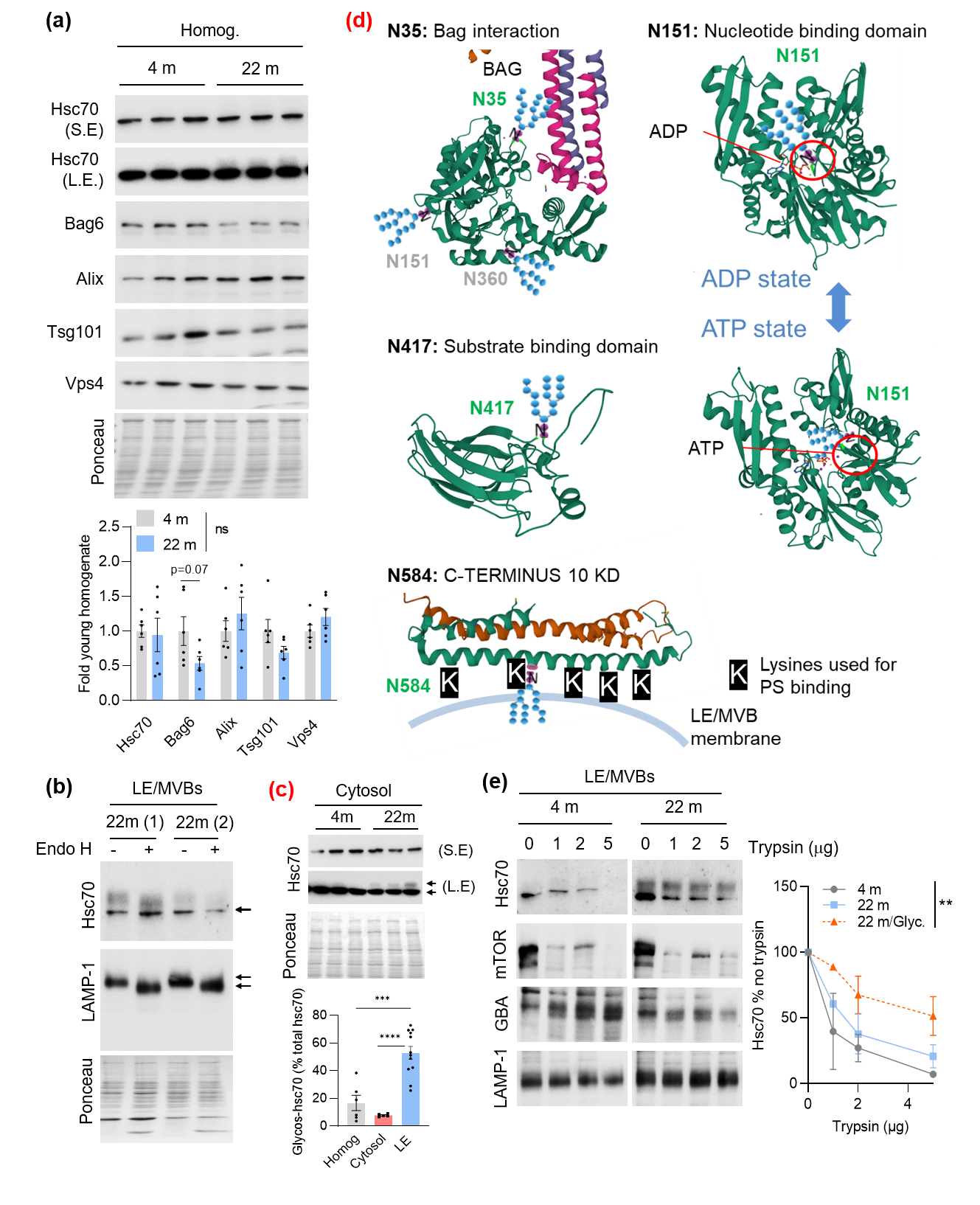
**

**Supplementary Figure 2. Characterization of changes in eMI components with age.** (a) Representative immunoblots (top) and quantification (bottom) for eMI components in liver homogenate from 4 m and 22 m old animals, matching LE/MVB fractions from Figure 2a. n=6 mice. Short exposure (S.E.) and long exposure (L.E.) images are shown. (b) Treatment of LE/MVBs from 22 m old animals with endoglycosidase enzyme Endo H. LAMP-1 is a control of a known glycosylated protein. n=4 mice. (c) Representative immunoblots (top) of cytosols from 4 m and 22 m old mice livers, matching LE/MVB fractions from Figure 2a. Quantification (bottom) of the fraction of Hsc70 glycosylated in each of the indicated compartments. Data from homogenate and LE from Figure 2d are been included for comparative statistical purposes. n=6 mice. (d) Crystal structure models of Hsc70 interaction with BAG domain-containing proteins (PBD:3CQX), Hsc70 in the ATP (PBD: 1KAX) and ADP (PBD: 2QWL) binding states, Hsc70 substrate binding region (PBD:1CKR) and Hsc70 lid (PBD:1UD0) with asparagine residues in each region identified as glycosylated in old LE/MVB marked in green with blue glycosylation cartoon. Lysines known to participate in phosphatidylserine binding on the LE/MVB membrane are indicated in black in the c-terminus model. (e) LE/MVB topology of glycosylated Hsc70. Left: Representative immunoblots of 4 m and 22 m old LE/MVBs incubated with increasing concentrations of trypsin. GBA, LAMP-1 and mTOR are shown as controls of luminal, transmembrane and membrane surface proteins, respectively. Right: Quantification of protein remaining for each concentration expressed as percentage of control condition incubated without trypsin. N=2 independent experiments. Data are mean+s.e.m. and individual values are shown in (a). One sample multiple t-tests (a) and two-way ANOVA with Bonferroni’s multiple comparisons post hoc test (e) were used. ns: not significant.

**
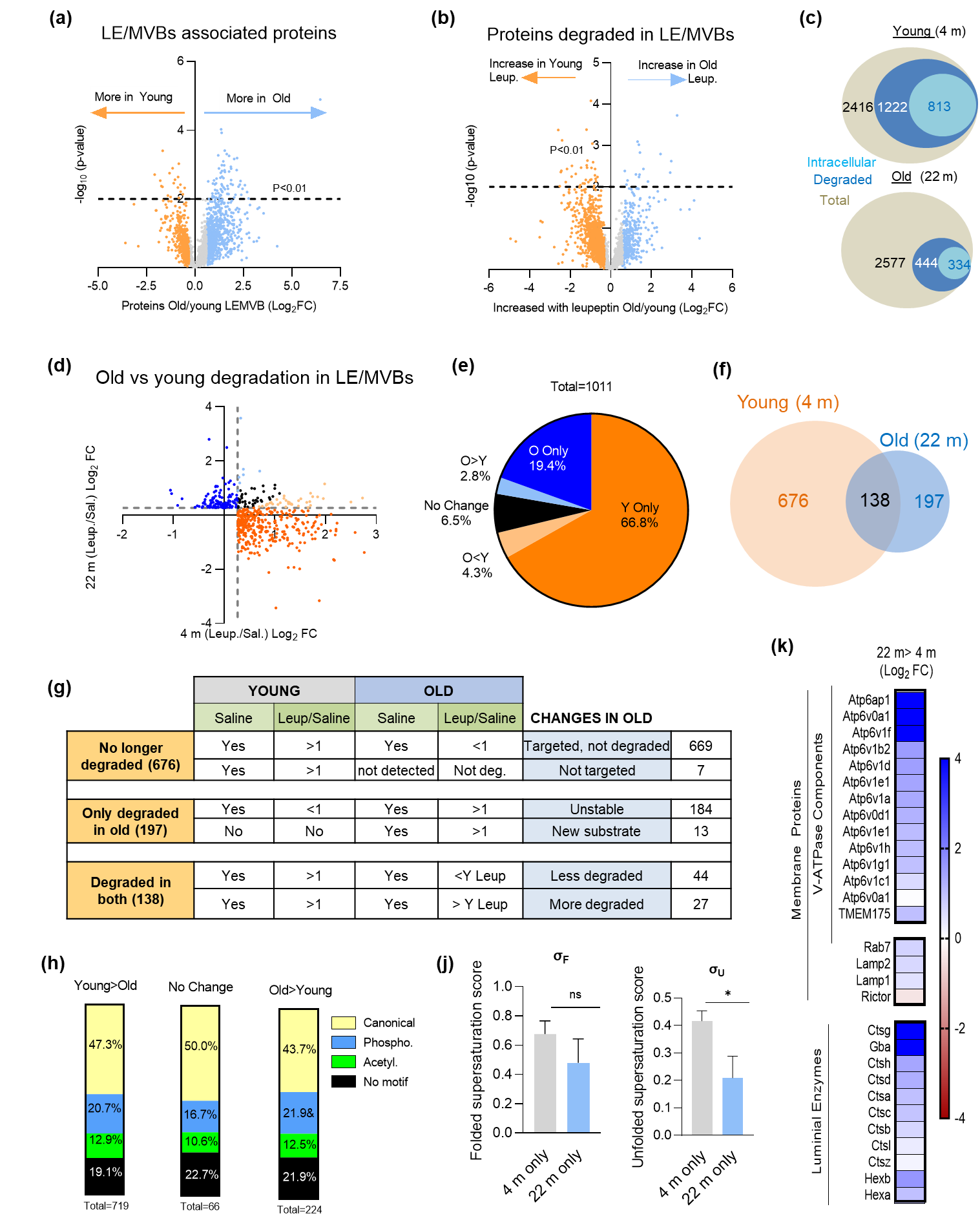
**

**Supplemental Figure 3. Changes with age in the sub-proteome degraded by eMI.** Comparative quantitative proteomics of LE/MVBs isolated from livers of 4 m and 22 m old mice injected or not with leupeptin (Leup.) to block endolysosomal degradation. Volcano plot showing log_2_ fold change (FC) in protein abundance between 4 m and 22 m old LE/MVBs in basal conditions (saline injection) (a) and after Leupeptin injection (b). Origin and degradation susceptibility of proteins detected in 4 m (top) and 22 m old (bottom) LE/MVBs (c). Log_2_FC in rates of protein degradation in LE/MVBs from 4 m and 22 m old mice. Blue: only (Dark) or increased (light) degradation in old. Orange: absent (dark) or reduced (light) degradation in old. Black: equal degradation in both groups (d). Percentage of proteins undergoing degradation in each identified category (e) and Venn Diagram of unique and shared LE/MVB substrates between ages (f). (g) Table categorizing changes in the degradation of proteins in LE/MVB from 22 m old identified in (f). (h) Analysis of the KFERQ-like motifs in the groups of proteins shown in (e). (i) Supersaturation scores in the folded state (σ_F_, left) or the unfolded state (σ_U_, right) of proteins degraded in LE/MVBs from 4 m old animals (Y only) or 22 m old animals (O only). Data are mean+s.e.m. (j) Heat map of Log_2_ fold change (FC) in protein abundance between 4 m and 22 m old mice in the indicated LE/MVBs proteins. Unpaired two-tailed t test was used in (j). Differences were significant for *p<0.01. ns: not significant.

**
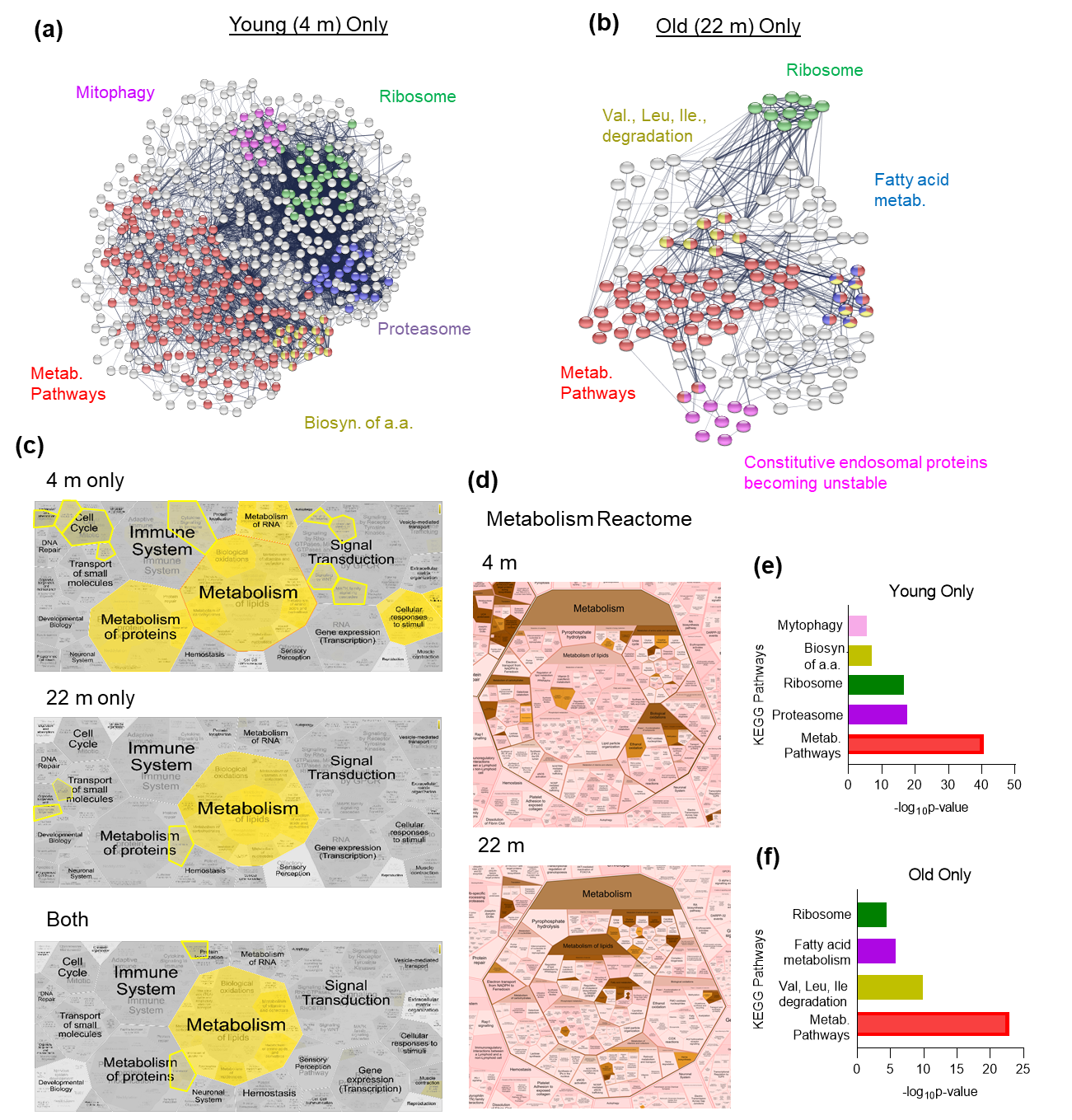
**

**Supplemental Figure 4. Age-related changes in the intracellular proteome degraded in LE/MVBs.** (a,b) STRING analysis of proteins degraded exclusively in 4 m or 22 m old LE/MVBs, respectively. (c,d) Voronoi flattened visualization Reactome pathway analysis of intracellular proteins undergoing degradation in LE/MVBs in the indicated age groups (c) and of metabolism-related pathways (d). (e,f) Differential enrichment of functional protein families in metabolic pathways of substrates degraded in LE/MVBs only in 4 m old (e) and only in 22 m old (f) mice. All GO terms are statistically enriched with p<0.001.

**
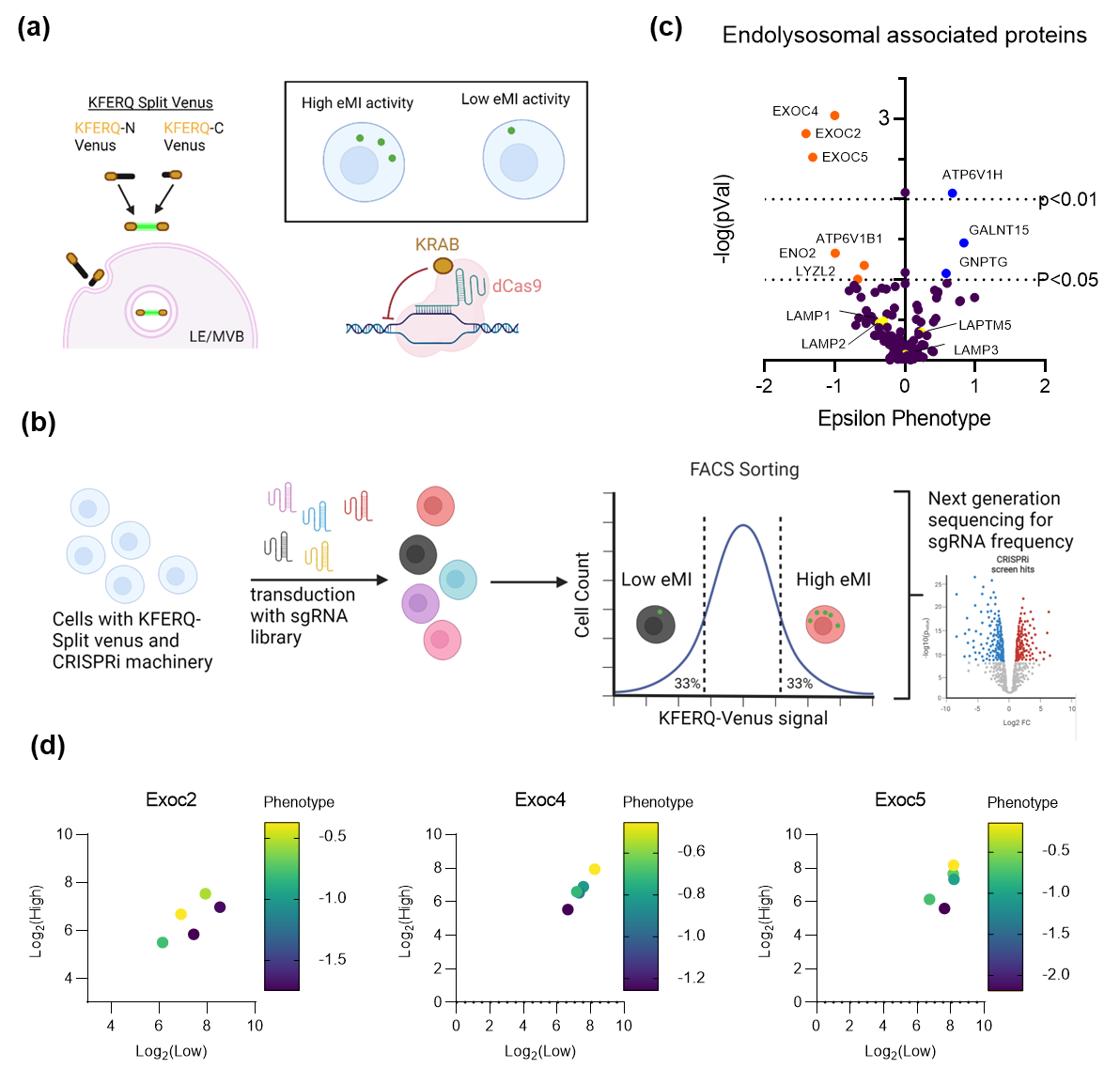
**

**Supplemental Figure 5. CRISPRi screen for basal eMI activity.** (a) Schematic of cells used for CRISPRi screen. Left: basis of the fluorescent KFERQ-Split Venus reporter, in which two fragments of the Venus protein are tagged with KFERQ-like motifs for eMI targeting. Right: examples of reporter read-outs for cells with high and low eMI (top) and scheme of nuclease dead Cas9 (dCas9) fused to the transcriptional repressor KRAB (dCas9-KRAB) expressed in the screen cells (bottom). (b) Experimental workflow for CRISPRi screen. Cells stably expressing the KFERQ-Split Venus reporter and the CRISPRi machinery were transduced with a pooled lentivirus library containing sgRNAs targeting proteostasis genes. After selection, cells were separated based on KFERQ-Split Venus fluorescence into high (top 33%) and low (bottom 33%) eMI populations, and genomic DNA was extracted for sequencing to identify sgRNA frequency in eMI high and eMI low populations. (c) Volcano plot of selected knockdown phenotypes (as epsilon values) for genes of the proteostasis library related with the endolysosomal system and their statistical significance (two-sided Mann-Whitney *U* Test). (d) Scatter plots of Log_2_ fold enrichment of counts for each of the 5 sgRNA tested for genes of the exocyst complex in eMI high (y-axis) and eMI low (x-axis) populations. sgRNA color indicates intensity of phenotype (enrichment in eMI high or eMI low population).

**
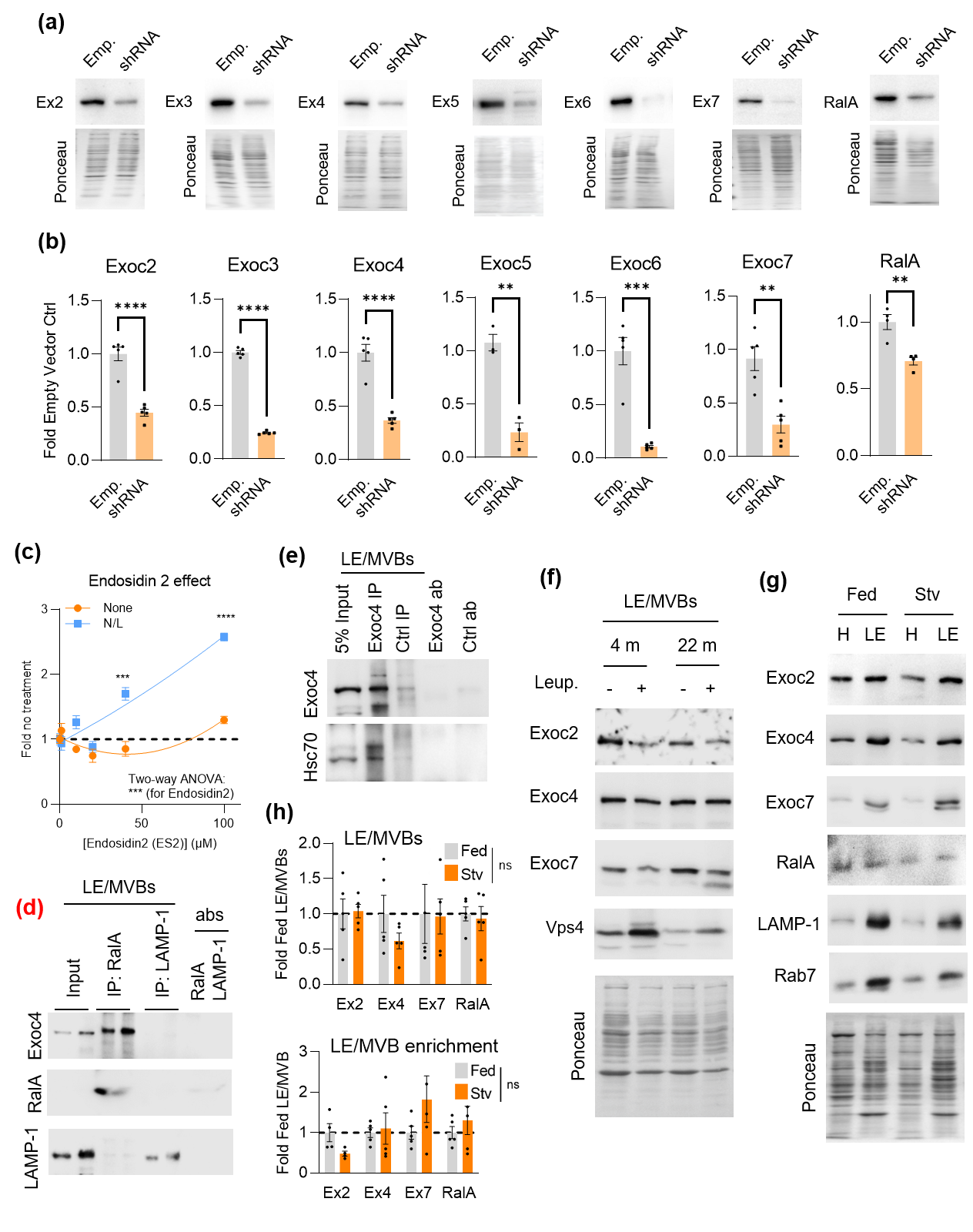
**

**Supplementary Figure 6. Characterization of the exocyst complex as a regulator of eMI in LE/MVBs.** (a,b) Immunoblot (a) and quantification (b) to demonstrate the efficacy of knock-down of the indicated proteins in NIH3T3 mouse fibroblasts. Values expressed as relative to those in cells transfected with an empty vector (Emp.). n=5 independent experiments (ie). (c) Quantification of eMI in NIH3T3 fibroblasts stably expressing the KFERQ-split Venus reporter treated with the exocyst complex inhibitor Endosidin2 (ES2) and incubated alone (None) or in the presence of N/L. Values are expressed as number of fluorescent puncta per cell relative to those in untreated cells. n≥2,500 cells from 1 ie. (d) Immunoblot for the indicated proteins after immunoprecipitation (IP) of LE/MVB with antibodies against RalA and LAMP-1 (L1) as positive and negative controls of interaction with Exoc4 for main Figure 6e. Antibodies used for IP are shown on the right. n=2 ie. (e) Immunoblot for the indicated proteins after immunoprecipitation (IP) of LE/MVB with an antibody against Exoc4 or control IgG (Ctrl). Antibodies used for IP are shown on the right. n=2 ie. (f) Immunoblots for the indicated proteins in LE/MVBs isolated from 4 m and 22 m old animals injected with saline (-) or with the endolysosomal protease inhibitor leupeptin (+). n=4 independent experiments (ie). (g,h) Representative immunoblots (g), quantification of protein levels (h top) and enrichment in LE/MVB over the respective homogenates (h bottom) for the indicated exocyst complex proteins in mice Fed and starved (Stv) for 24 hours. n=5 mice. Data are mean+s.e.m. and individual values. Unpaired two-sided t-test (b), two-way ANOVA with Tukey’s multiple comparisons post hoc test (c), and one sample multiple t-tests (h) were used. Differences were significant for *p<0.05, **p<0.01, ***p<0.001 ****p<0.0001. ns: not significant.

**
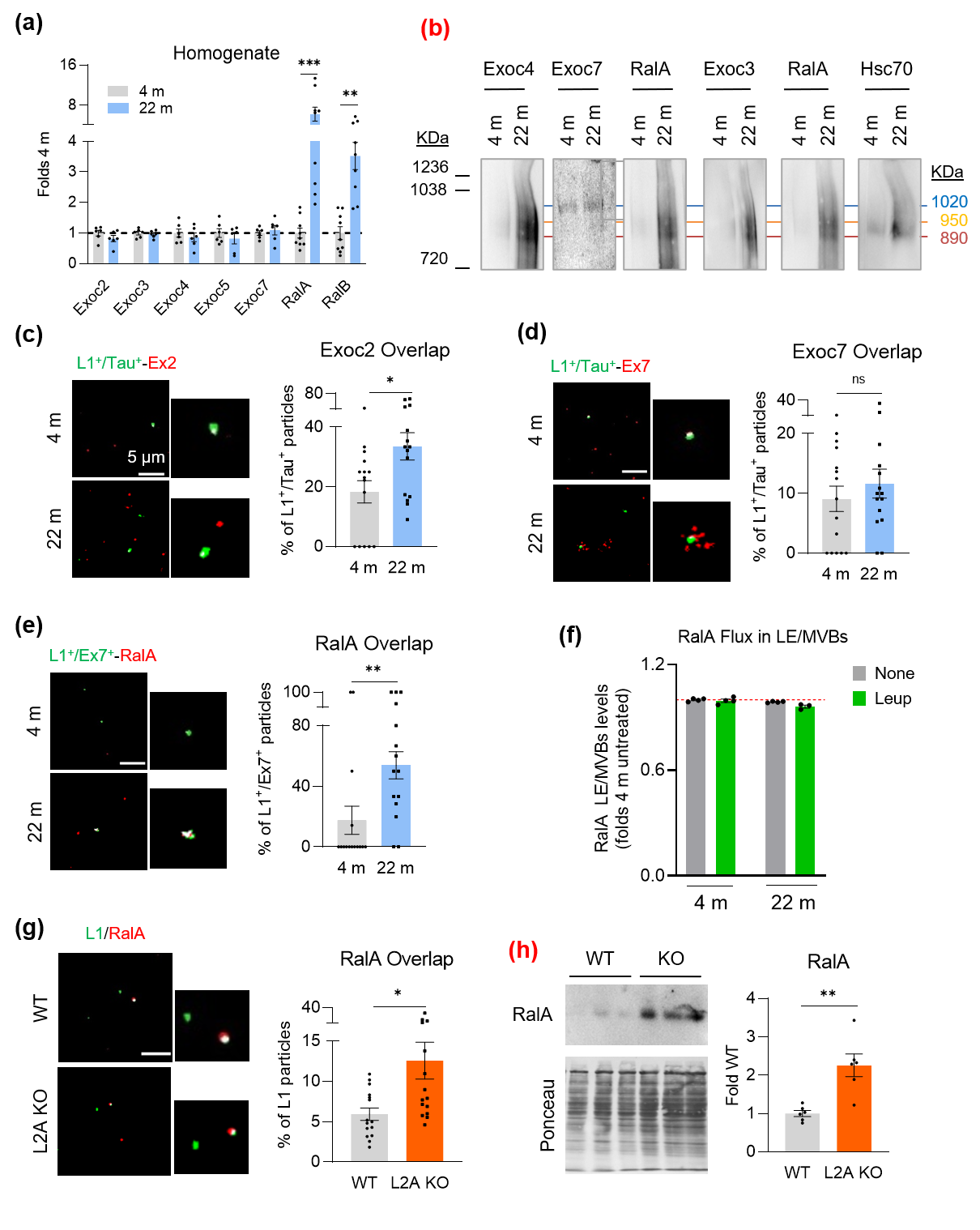
**

**Supplementary Figure 7. Changes in the exocyst complex in LE/MVBs with age.** (a) Quantification of exocyst component abundance in total liver homogenate from samples shown in Figure 7a. n=6 (Exoc2, Exoc3, Exoc4, Exoc5, Exoc7) or 9 (RalA, RalB) mice. (b) High molecular weight region of the representative immunoblots for the indicated proteins after blue-native electrophoresis of 4 m and 22 m old LE/MVBs shown in main Figure 7e,f). Samples for Exoc 4, Exoc 7, and RalA were run in one membrane and for Exoc 3, RalA and Hsc70 in a second membrane. RalA complexes were used for alignment between membranes. Main identified complexes are marked by color lines with their corresponding calculated molecular weight on the right. (c-e) Representative confocal microscopy images with colocalization masks of LE/MVBs isolated from 4 m and 22 m old mice incubated (c, d) or not (e) with tau protein and stained with the indicated antibodies. Colocalization masks represent overlap of all three fluorophores with respect to double positive particles in green. Insets: higher magnification images. Right: Quantification of particle colocalization with respect to indicated reference population. n=15 fields from 3 mice of each group. (f) Levels of RalA detected by mass spectrometry in LE/MVBs isolated from 4 m and 22 m old mice injected with saline (None) or with leupeptin (Leup). (g) Representative confocal microscopy images with colocalization masks for LAMP-1 and RalA in LE/MVBs isolated from wild-type (WT) or L2A knock-out mice (L2A KO). Insets: higher magnification images. Right: Quantification of percentage of LAMP-1 positive LE/MVBs also positive for RalA. n=15 fields from 3 mice of each group. (h) Abundance of RalA in liver homogenate from WT or L2A KO mice, with representative immunoblot (left) and quantification (right). Data are mean+s.e.m. and individual values. One sample multiple t-tests (a) and unpaired two-sided t-tests (c-e, g, h) were used. Differences were significant for *p<0.05, **p<0.01, ***p<0.001. ns: not significant

**
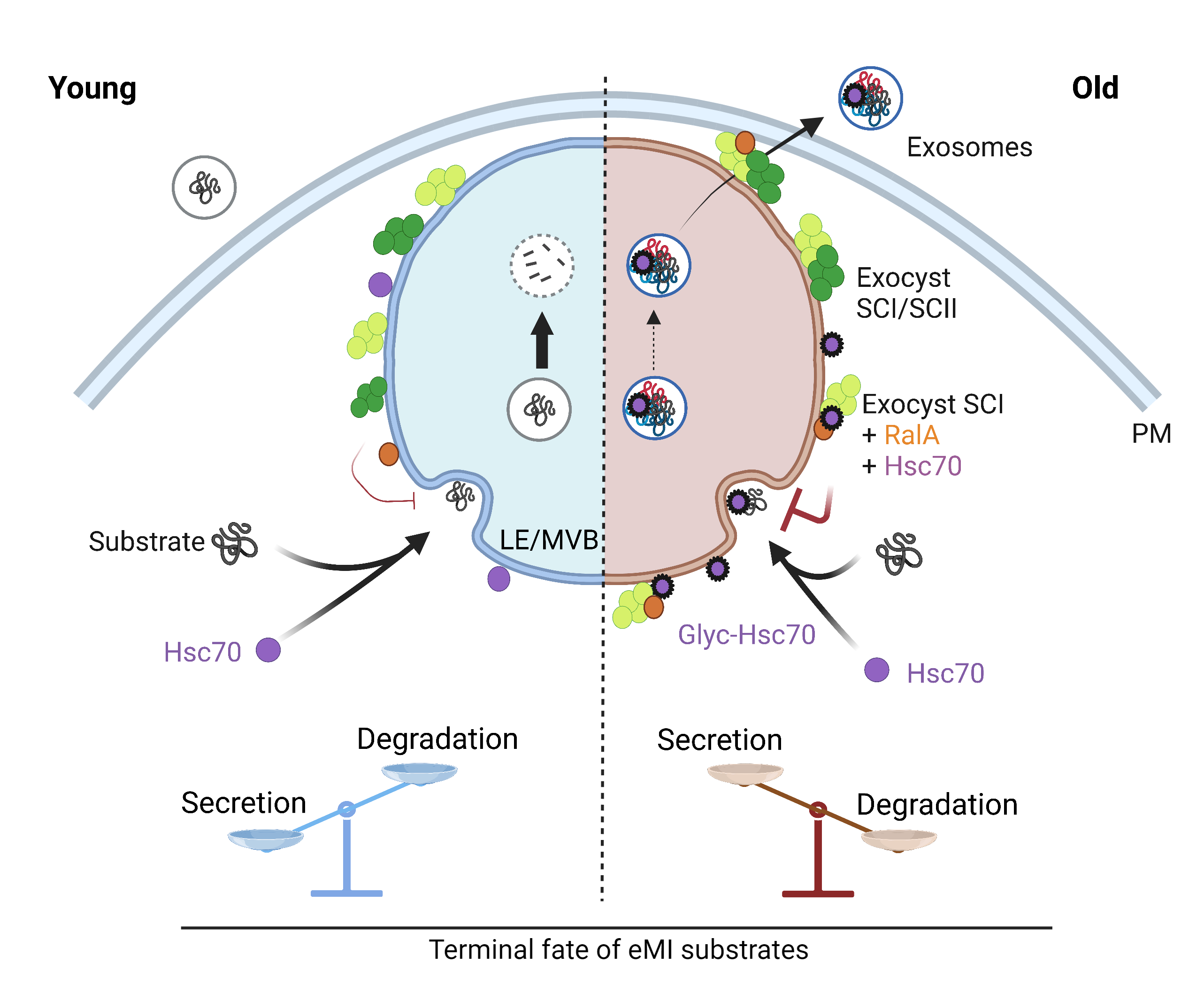
**

**Supplementary Figure 8. Working model of the basis and consequences of eMI malfunctioning in aging.** *Left:* Endosomal microautophagy (eMI) is responsible for selective autophagy of cytosolic proteins targeted to late endosomes/multivesicular bodies (LE/MVBs) by the chaperone Hsc70. In this work, we have identified that components of the exocyst complex and their associated GTPase RalA, exert a physiological negative regulatory role on eMI at the level of substrate internalization.r *Right: eMI* activity decreases in aging due to age-related changes in the LE/MVB including: i) altered membrane dynamics and age-dependent glycation of Hsc70, ii) elevated levels of RalA, iii) formation of high molecular weight complexes containing glycated Hsc70, RalA and subunits of the exocyst complex I (SCI) and iv) increased in abundance of SCI and SCII containing complexes. We propose that these changes in the organization of the exocyst complexes and RalA in old LE/MVBs and mediates fusion of this compartment with the plasma membrane (PM) and the subsequent extracellular release of proteins. We propose that increased recruitment of RalA to LE/MVBs with age mediates a switch from protein intracellular degradation to protein secretion.

**Supplementary Tables**

**Table S1. Sequence of shRNA used in this work**

| **Species** | **Gene** | **Oligo ID** | **Sequence (5'-3')** | **Source** |
| --- | --- | --- | --- | --- |
| *Mus musculus* | Exoc2 | TRCN0000195832 | CGCAGGCTACTTTGATTGGAA | TRC Genome-Wide shRNA collection |
| *Mus musculus* | Exoc3 | TRCN0000111625 | CCTCTTGACATGGGTCCTAAA | TRC Genome-Wide shRNA collection |
| *Mus musculus* | Exoc4 | TRCN0000111727 | CCAGAGTATCACAGAACGCAT | TRC Genome-Wide shRNA collection |
| *Mus musculus* | Exoc5 | TRCN0000093548 | GCACAGGAGGTATTCAAGATT | TRC Genome-Wide shRNA collection |
| *Mus musculus* | Exoc6 | TRCN0000115296 | CGTGAGACAATAACTTGTTTA | TRC Genome-Wide shRNA collection |
| *Mus musculus* | Exoc7 | TRCN0000191757 | GCAGATGATTAAGGAACGTTT | TRC Genome-Wide shRNA collection |
| *Mus musculus* | RalA | TRCN0000077722 | CAGGAGGACTATGCTGCAATT | TRC Genome-Wide shRNA collection |
| *Mus musculus* | shRNA control | TRC empty vector |  | TRC Genome-Wide shRNA collection |

**Table S2. Sequence of qPCR primers used in this work**

| **Gene** | **Species** | **Source** |  | **sequence (5'-3')** |
| --- | --- | --- | --- | --- |
| RalA | *Mus musculus* | Eurofins | Forward | GCTCGCGGTGCAGATTTTT |
|  |  |  | Reverse | TCCTGTAGCTGTCCGCTTTG |
| β-actin | *Mus musculus* | Eurofins | Forward | AAGGACTCCTATAGTGGGTGACGA |
|  |  |  | Reverse | ATCTTCTCCATGTCGTCCCAGTTG |

**Table S3. CRISPRi results from genes elated with the endolysosomal system**

| #TSS | Symbol | GeneInfo | Epsilon # sgRNAs | Epsilon Phenotype | Epsilon P value | log pValue |
| --- | --- | --- | --- | --- | --- | --- |
| EXOC2__P1P2 | EXOC2 | exocyst complex component 2 | 5 | -1.4115 | 0.0015 | 2.8155 |
| EXOC5__P1P2 | EXOC5 | exocyst complex component 5 | 5 | -1.3162 | 0.0030 | 2.5218 |
| EXOC4__P1P2 | EXOC4 | exocyst complex component 4 | 5 | -1.0026 | 0.0009 | 3.0394 |
| ENO2__P1P2 | ENO2 | enolase 2 (gamma, neuronal) | 5 | -0.9958 | 0.0469 | 1.3288 |
| LYZL2__P1P2 | LYZL2 | lysozyme-like 2 | 5 | -0.6765 | 0.0989 | 1.0049 |
| ATP6V1B1__P1P2 | ATP6V1B1 | ATPase, H+ transporting, lysosomal 56/58kDa, V1 subunit B1 | 5 | -0.5830 | 0.0666 | 1.1762 |
| GNPTG__P1P2 | GNPTG | N-acetylglucosamine-1-phosphate transferase, gamma subunit | 5 | 0.5857 | 0.0836 | 1.0776 |
| ATP6V1H__P1P2 | ATP6V1H | ATPase, H+ transporting, lysosomal 50/57kDa, V1 subunit H | 5 | 0.6748 | 0.0084 | 2.0766 |
| GALNT15__P1P2 | GALNT15 | UDP-N-acetyl-alpha-D-galactosamine:polypeptide N-acetylgalactosaminyltransferase 15 | 5 | 0.8380 | 0.0349 | 1.4573 |
| ATP6V1A__P1P2 | ATP6V1A | ATPase, H+ transporting, lysosomal 70kDa, V1 subunit A | 5 | 0.0000 | 0.0082 | 2.0845 |
| ATP6AP1__P1P2 | ATP6AP1 | ATPase, H+ transporting, lysosomal accessory protein 1 | 5 | 0.0000 | 0.0815 | 1.0890 |
| GUSB__P1P2 | GUSB | glucuronidase, beta | 5 | 0.5985 | 0.1107 | 0.9560 |
| ATP6V1B2__P1P2 | ATP6V1B2 | ATPase, H+ transporting, lysosomal 56/58kDa, V1 subunit B2 | 5 | 0.0000 | 0.1120 | 0.9506 |
| PIK3CA__P1P2 | PIK3CA | phosphatidylinositol-4,5-bisphosphate 3-kinase, catalytic subunit alpha | 5 | -0.7254 | 0.1177 | 0.9293 |
| ATP6V0A4__P1 | ATP6V0A4 | ATPase, H+ transporting, lysosomal V0 subunit a4 | 5 | -0.1403 | 0.1220 | 0.9135 |
| ATG4D__P1P2 | ATG4D | autophagy related 4D, cysteine peptidase | 5 | -0.3327 | 0.1281 | 0.8925 |
| SIAE__P1P2 | SIAE | sialic acid acetylesterase | 5 | -0.6869 | 0.1296 | 0.8873 |
| MANBA__P1P2 | MANBA | mannosidase, beta A, lysosomal | 5 | -0.2674 | 0.1312 | 0.8822 |
| SERPINA1__P1 | SERPINA1 | serpin peptidase inhibitor, clade A (alpha-1 antiproteinase, antitrypsin), member 1 | 5 | 0.4231 | 0.1359 | 0.8667 |
| ATP6V1C1__P1P2 | ATP6V1C1 | ATPase, H+ transporting, lysosomal 42kDa, V1 subunit C1 | 5 | -0.7946 | 0.1375 | 0.8616 |
| HYAL1__P1 | HYAL1 | hyaluronoglucosaminidase 1 | 5 | 0.4670 | 0.1425 | 0.8463 |
| SH3GLB1__P1P2 | SH3GLB1 | SH3-domain GRB2-like endophilin B1 | 5 | -0.4278 | 0.1442 | 0.8412 |
| PIK3CB__P1P2 | PIK3CB | phosphatidylinositol-4,5-bisphosphate 3-kinase, catalytic subunit beta | 5 | 0.4632 | 0.1493 | 0.8260 |
| ACACA__P1P2 | ACACA | acetyl-CoA carboxylase alpha | 5 | -0.6267 | 0.1654 | 0.7814 |
| NPC2__P1P2 | NPC2 | Niemann-Pick disease, type C2 | 5 | 0.9929 | 0.1673 | 0.7765 |
| MOGS__P1P2 | MOGS | mannosyl-oligosaccharide glucosidase | 5 | 0.4484 | 0.1692 | 0.7716 |
| NPC1__P1P2 | NPC1 | Niemann-Pick disease, type C1 | 5 | 0.7873 | 0.1829 | 0.7378 |
| ATP6V0E2__P1P2 | ATP6V0E2 | ATPase, H+ transporting V0 subunit e2 | 5 | 0.7798 | 0.1869 | 0.7283 |
| PLA2G15__P1P2 | PLA2G15 | phospholipase A2, group XV | 5 | 0.1693 | 0.2082 | 0.6814 |
| HS3ST3B1__P1P2 | HS3ST3B1 | heparan sulfate (glucosamine) 3-O-sulfotransferase 3B1 | 5 | -0.1514 | 0.2265 | 0.6449 |
| TCIRG1__P1P2 | TCIRG1 | T-cell, immune regulator 1, ATPase, H+ transporting, lysosomal V0 subunit A3 | 5 | 0.1876 | 0.2360 | 0.6270 |
| GBA__P1 | GBA | glucosidase, beta, acid | 5 | -0.5521 | 0.2434 | 0.6137 |
| ATP6V1G2__P1P2 | ATP6V1G2 | ATPase, H+ transporting, lysosomal 13kDa, V1 subunit G2 | 5 | -0.2582 | 0.2664 | 0.5745 |
| ATP6V1D__P1P2 | ATP6V1D | ATPase, H+ transporting, lysosomal 34kDa, V1 subunit D | 5 | -0.1834 | 0.2771 | 0.5574 |
| ATP6V0B__P1P2 | ATP6V0B | ATPase, H+ transporting, lysosomal 21kDa, V0 subunit b | 5 | -0.4777 | 0.2880 | 0.5406 |
| ATP6V1E2__P1P2 | ATP6V1E2 | ATPase, H+ transporting, lysosomal 31kDa, V1 subunit E2 | 5 | -0.6579 | 0.2993 | 0.5239 |
| MAN2A1__P1P2 | MAN2A1 | mannosidase, alpha, class 2A, member 1 | 5 | 0.4382 | 0.3036 | 0.5177 |
| ACP2__P1P2 | ACP2 | acid phosphatase 2, lysosomal | 5 | -0.5449 | 0.3079 | 0.5116 |
| CPN2__P1P2 | CPN2 | carboxypeptidase N, polypeptide 2 | 5 | 0.1623 | 0.3108 | 0.5075 |
| PIK3C2A__ENST00000265970.7 | PIK3C2A | phosphatidylinositol-4-phosphate 3-kinase, catalytic subunit type 2 alpha | 5 | -0.4351 | 0.3257 | 0.4872 |
| LAMP2__P1P2 | LAMP2 | lysosomal-associated membrane protein 2 | 5 | -0.3083 | 0.3287 | 0.4832 |
| NEU4__P2 | NEU4 | sialidase 4 | 5 | 0.4048 | 0.3348 | 0.4752 |
| NEU1__P1P2 | NEU1 | sialidase 1 (lysosomal sialidase) | 5 | 0.2234 | 0.3379 | 0.4712 |
| LAMP1__P1P2 | LAMP1 | lysosomal-associated membrane protein 1 | 5 | -0.3640 | 0.3472 | 0.4594 |
| ATP6V0D2__P1P2 | ATP6V0D2 | ATPase, H+ transporting, lysosomal 38kDa, V0 subunit d2 | 5 | -0.5538 | 0.3504 | 0.4555 |
| GALNS__P1P2 | GALNS | galactosamine (N-acetyl)-6-sulfate sulfatase | 5 | -0.7012 | 0.3697 | 0.4322 |
| CPB1__P1P2 | CPB1 | carboxypeptidase B1 (tissue) | 5 | -0.2561 | 0.3930 | 0.4057 |
| HEXB__P1P2 | HEXB | hexosaminidase B (beta polypeptide) | 5 | -0.3706 | 0.3964 | 0.4019 |
| HYAL2__P2 | HYAL2 | hyaluronoglucosaminidase 2 | 5 | 0.1986 | 0.4422 | 0.3544 |
| DPP4__P1P2 | DPP4 | dipeptidyl-peptidase 4 | 5 | -0.0643 | 0.4458 | 0.3508 |
| LAPTM5__P1P2 | LAPTM5 | lysosomal protein transmembrane 5 | 5 | 0.2341 | 0.4532 | 0.3437 |
| ATP6V0A2__P1P2 | ATP6V0A2 | ATPase, H+ transporting, lysosomal V0 subunit a2 | 5 | 0.1973 | 0.4794 | 0.3193 |
| ATP6V1E1__P1P2 | ATP6V1E1 | ATPase, H+ transporting, lysosomal 31kDa, V1 subunit E1 | 5 | -0.3393 | 0.4813 | 0.3176 |
| GLA__P1P2 | GLA | galactosidase, alpha | 5 | -0.4343 | 0.4909 | 0.3090 |
| ASRGL1__P1P2 | ASRGL1 | asparaginase like 1 | 5 | -0.3494 | 0.4947 | 0.3056 |
| GNPTAB__P1P2 | GNPTAB | N-acetylglucosamine-1-phosphate transferase, alpha and beta subunits | 5 | 0.3133 | 0.4947 | 0.3056 |
| ATP6V0C__P1P2 | ATP6V0C | ATPase, H+ transporting, lysosomal 16kDa, V0 subunit c | 5 | -0.4139 | 0.4986 | 0.3022 |
| FUCA1__P1P2 | FUCA1 | fucosidase, alpha-L- 1, tissue | 5 | -0.1966 | 0.4986 | 0.3022 |
| GLB1__P1P2 | GLB1 | galactosidase, beta 1 | 5 | -0.2427 | 0.4986 | 0.3022 |
| IDUA__P1P2 | IDUA | iduronidase, alpha-L- | 5 | -0.2967 | 0.5084 | 0.2938 |
| GLB1L__P1 | GLB1L | galactosidase, beta 1-like | 5 | -0.0824 | 0.5262 | 0.2788 |
| ARSD__P1P2 | ARSD | arylsulfatase D | 5 | -0.1158 | 0.5383 | 0.2690 |
| ACP5__P1 | ACP5 | acid phosphatase 5, tartrate resistant | 5 | 0.1209 | 0.5711 | 0.2433 |
| PGAM5__P1P2 | PGAM5 | phosphoglycerate mutase family member 5 | 5 | -0.0704 | 0.5753 | 0.2401 |
| GGT2__ENST00000401924.1 | GGT2 | gamma-glutamyltransferase 2 | 5 | -0.2982 | 0.5836 | 0.2339 |
| GLB1L__P2 | GLB1L | galactosidase, beta 1-like | 5 | -0.2532 | 0.5900 | 0.2292 |
| ATP6V0A1__P1P2 | ATP6V0A1 | ATPase, H+ transporting, lysosomal V0 subunit a1 | 5 | -0.2565 | 0.5942 | 0.2261 |
| ASAH1__P1P2 | ASAH1 | N-acylsphingosine amidohydrolase (acid ceramidase) 1 | 5 | -0.2183 | 0.5963 | 0.2245 |
| ALPL__P2 | ALPL | alkaline phosphatase, liver/bone/kidney | 5 | -0.1116 | 0.6134 | 0.2122 |
| DNASE2__P1P2 | DNASE2 | deoxyribonuclease II, lysosomal | 5 | -0.0873 | 0.6177 | 0.2092 |
| NAAA__P1P2 | NAAA | N-acylethanolamine acid amidase | 5 | 0.0731 | 0.6307 | 0.2002 |
| PLD1__P1P2 | PLD1 | phospholipase D1, phosphatidylcholine-specific | 5 | -0.1620 | 0.6307 | 0.2002 |
| NAGLU__P1P2 | NAGLU | N-acetylglucosaminidase, alpha | 5 | -0.2245 | 0.6395 | 0.1942 |
| GALC__P1P2 | GALC | galactosylceramidase | 5 | 0.2426 | 0.6438 | 0.1912 |
| PM20D1__P1P2 | PM20D1 | peptidase M20 domain containing 1 | 5 | -0.0740 | 0.6482 | 0.1883 |
| GBA__P2 | GBA | glucosidase, beta, acid | 5 | 0.2702 | 0.6526 | 0.1853 |
| GSAP__P1P2 | GSAP | gamma-secretase activating protein | 5 | -0.0154 | 0.6615 | 0.1795 |
| ATP6V0A4__P2 | ATP6V0A4 | ATPase, H+ transporting, lysosomal V0 subunit a4 | 5 | -0.0748 | 0.6704 | 0.1737 |
| GANAB__P1P2 | GANAB | glucosidase, alpha; neutral AB | 5 | -0.1829 | 0.6749 | 0.1708 |
| EEA1__P1P2 | EEA1 | early endosome antigen 1 | 5 | -0.1185 | 0.6793 | 0.1679 |
| LIPK__ENST00000404190.1 | LIPK | lipase, family member K | 5 | -0.0508 | 0.7110 | 0.1481 |
| OSGEP__P1P2 | OSGEP | O-sialoglycoprotein endopeptidase | 5 | 0.1132 | 0.7110 | 0.1481 |
| DNASE2B__P1P2 | DNASE2B | deoxyribonuclease II beta | 5 | -0.1583 | 0.7248 | 0.1398 |
| DPP7__P1 | DPP7 | dipeptidyl-peptidase 7 | 5 | 0.1368 | 0.7248 | 0.1398 |
| PIK3C2B__P1P2 | PIK3C2B | phosphatidylinositol-4-phosphate 3-kinase, catalytic subunit type 2 beta | 5 | 0.1800 | 0.7248 | 0.1398 |
| ATG4C__P1P2 | ATG4C | autophagy related 4C, cysteine peptidase | 5 | 0.0835 | 0.7478 | 0.1262 |
| HYAL1__P2 | HYAL1 | hyaluronoglucosaminidase 1 | 5 | -0.1343 | 0.7571 | 0.1208 |
| HYAL3__P1P2 | HYAL3 | hyaluronoglucosaminidase 3 | 5 | -0.1343 | 0.7571 | 0.1208 |
| MAN2B1__P2 | MAN2B1 | mannosidase, alpha, class 2B, member 1 | 5 | 0.3813 | 0.7665 | 0.1155 |
| LIPA__P1P2 | LIPA | lipase A, lysosomal acid, cholesterol esterase | 5 | 0.4033 | 0.7876 | 0.1037 |
| PLCD4__P1P2 | PLCD4 | phospholipase C, delta 4 | 5 | -0.0727 | 0.8041 | 0.0947 |
| SMPD1__P1P2 | SMPD1 | sphingomyelin phosphodiesterase 1, acid lysosomal | 5 | 0.1468 | 0.8089 | 0.0921 |
| ATG4A__P1P2 | ATG4A | autophagy related 4A, cysteine peptidase | 5 | 0.1733 | 0.8136 | 0.0896 |
| HGSNAT__P1P2 | HGSNAT | heparan-alpha-glucosaminide N-acetyltransferase | 5 | 0.0612 | 0.8136 | 0.0896 |
| GGH__P1P2 | GGH | gamma-glutamyl hydrolase (conjugase, folylpolygammaglutamyl hydrolase) | 5 | -0.1028 | 0.8184 | 0.0870 |
| ATP6V1C2__P1P2 | ATP6V1C2 | ATPase, H+ transporting, lysosomal 42kDa, V1 subunit C2 | 5 | -0.0261 | 0.8279 | 0.0820 |
| ATP6V0D1__P1P2 | ATP6V0D1 | ATPase, H+ transporting, lysosomal 38kDa, V0 subunit d1 | 5 | -0.1163 | 0.8327 | 0.0795 |
| CTNS__P1P2 | CTNS | cystinosin, lysosomal cystine transporter | 5 | -0.1731 | 0.8470 | 0.0721 |
| ATG4B__P1P2 | ATG4B | autophagy related 4B, cysteine peptidase | 5 | 0.0606 | 0.8518 | 0.0696 |
| IDS__P1P2 | IDS | iduronate 2-sulfatase | 5 | 0.0223 | 0.8518 | 0.0696 |
| SMG1__P1P2 | SMG1 | smg-1 homolog, phosphatidylinositol 3-kinase-related kinase (C. elegans) | 5 | -0.0412 | 0.8518 | 0.0696 |
| ARSA__P1P2 | ARSA | arylsulfatase A | 5 | -0.1109 | 0.8566 | 0.0672 |
| LAMP3__P1P2 | LAMP3 | lysosomal-associated membrane protein 3 | 5 | 0.0056 | 0.8566 | 0.0672 |
| PIK3C3__P1P2 | PIK3C3 | phosphatidylinositol 3-kinase, catalytic subunit type 3 | 5 | -0.1416 | 0.8566 | 0.0672 |
| NEU4__P1 | NEU4 | sialidase 4 | 5 | 0.1184 | 0.8615 | 0.0648 |
| ATP6V1F__P1P2 | ATP6V1F | ATPase, H+ transporting, lysosomal 14kDa, V1 subunit F | 5 | -0.1276 | 0.8663 | 0.0623 |
| GAA__P1P2 | GAA | glucosidase, alpha; acid | 5 | -0.0806 | 0.8663 | 0.0623 |
| ALPL__P1 | ALPL | alkaline phosphatase, liver/bone/kidney | 5 | 0.0703 | 0.8807 | 0.0552 |
| MGAM__P1P2 | MGAM | maltase-glucoamylase (alpha-glucosidase) | 5 | 0.0327 | 0.8807 | 0.0552 |
| PIK3C2G__P1P2 | PIK3C2G | phosphatidylinositol-4-phosphate 3-kinase, catalytic subunit type 2 gamma | 5 | -0.0706 | 0.8904 | 0.0504 |
| ATP6V0E1__P1P2 | ATP6V0E1 | ATPase, H+ transporting, lysosomal 9kDa, V0 subunit e1 | 5 | 0.2228 | 0.8928 | 0.0492 |
| HYAL2__P1 | HYAL2 | hyaluronoglucosaminidase 2 | 5 | -0.1521 | 0.8953 | 0.0481 |
| MAN2B1__P1 | MAN2B1 | mannosidase, alpha, class 2B, member 1 | 5 | 0.1108 | 0.9001 | 0.0457 |
| ATP6AP1L__P1P2 | ATP6AP1L | ATPase, H+ transporting, lysosomal accessory protein 1-like | 5 | 0.1182 | 0.9049 | 0.0434 |
| MAN2C1__P1P2 | MAN2C1 | mannosidase, alpha, class 2C, member 1 | 5 | 0.0530 | 0.9049 | 0.0434 |
| DPP7__P2 | DPP7 | dipeptidyl-peptidase 7 | 5 | 0.0410 | 0.9098 | 0.0411 |
| PLCXD2__P1P2 | PLCXD2 | phosphatidylinositol-specific phospholipase C, X domain containing 2 | 5 | -0.1880 | 0.9098 | 0.0411 |
| FIG4__P1P2 | FIG4 | FIG4 homolog, SAC1 lipid phosphatase domain containing (S. cerevisiae) | 5 | 0.0477 | 0.9195 | 0.0364 |
| AGA__P1P2 | AGA | aspartylglucosaminidase | 5 | 0.2577 | 0.9244 | 0.0341 |
| ATP6V1G1__P1P2 | ATP6V1G1 | ATPase, H+ transporting, lysosomal 13kDa, V1 subunit G1 | 5 | -0.1105 | 0.9292 | 0.0319 |
| P4HB__P1P2 | P4HB | prolyl 4-hydroxylase, beta polypeptide | 5 | -0.0549 | 0.9341 | 0.0296 |
| ATHL1__P1P2 | ATHL1 | ATH1, acid trehalase-like 1 (yeast) | 5 | -0.2148 | 0.9365 | 0.0285 |
| PIK3CG__P1P2 | PIK3CG | phosphatidylinositol-4,5-bisphosphate 3-kinase, catalytic subunit gamma | 5 | -0.1131 | 0.9487 | 0.0229 |
| GANC__P1P2 | GANC | glucosidase, alpha; neutral C | 5 | -0.0514 | 0.9634 | 0.0162 |
| OSGEPL1__P1P2 | OSGEPL1 | O-sialoglycoprotein endopeptidase-like 1 | 5 | -0.1024 | 0.9634 | 0.0162 |
| CPA2__P1P2 | CPA2 | carboxypeptidase A2 (pancreatic) | 5 | 0.1230 | 0.9682 | 0.0140 |
| DNPEP__P1P2 | DNPEP | aspartyl aminopeptidase | 5 | -0.0372 | 0.9682 | 0.0140 |
| PRCP__P2 | PRCP | prolylcarboxypeptidase (angiotensinase C) | 5 | 0.0146 | 0.9682 | 0.0140 |
| RILP__P1P2 | RILP | Rab interacting lysosomal protein | 5 | -0.1007 | 0.9780 | 0.0097 |
| LYST__P1P2 | LYST | lysosomal trafficking regulator | 5 | 0.0770 | 0.9976 | 0.0011 |
| PI4K2B__P1P2 | PI4K2B | phosphatidylinositol 4-kinase type 2 beta | 5 | -0.2164 | 0.9976 | 0.0011 |

**Uncropped immunoblots**

**
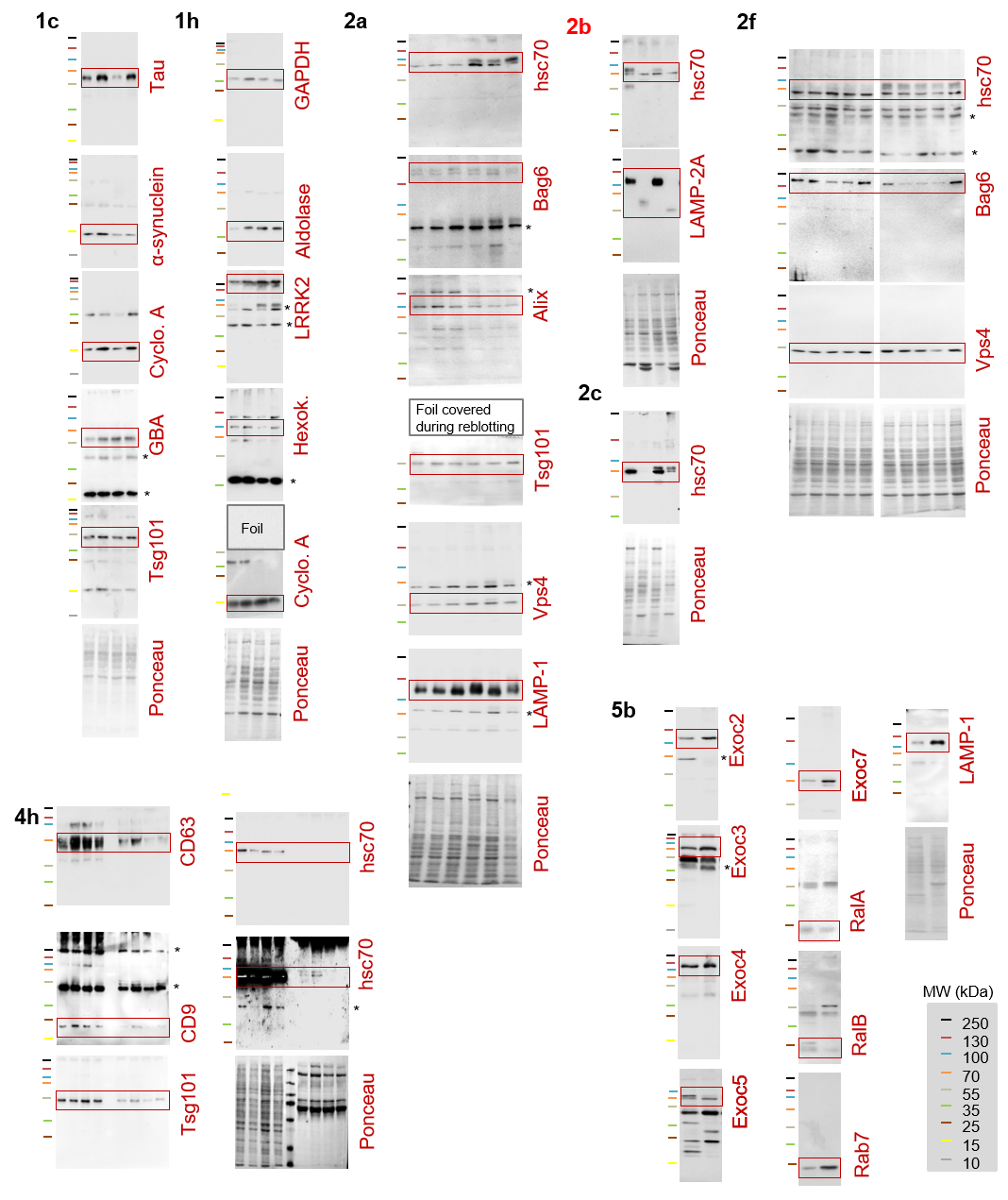
**

**Supplementary Data 1. Uncropped gels from Main Figures 1-5**. Dotted red boxes mark cropped areas shown in the main figure of the membrane blotted for the protein indicated on the right. Ponceau staining is shown in those instances in which total protein per line was used for normalization in the densitometric analysis.

**
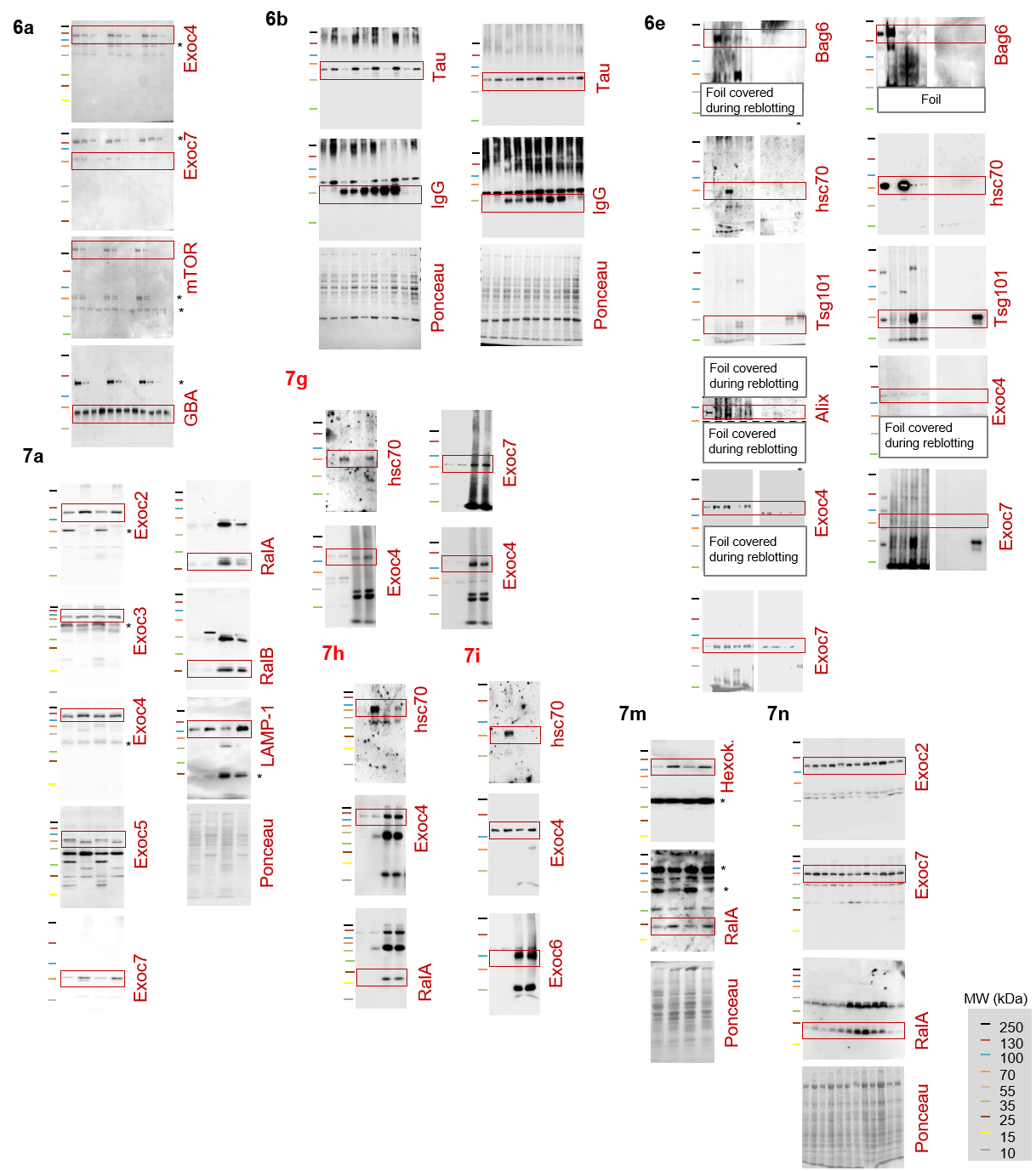
**

**Supplementary Data 2. Uncropped gels from Main Figures 6 and 7**. Dotted red boxes mark cropped areas shown in the main figure of the membrane blotted for the protein indicated on the right. Ponceau staining is shown in those instances in which total protein per line was used for normalization in the densitometric analysis.

**
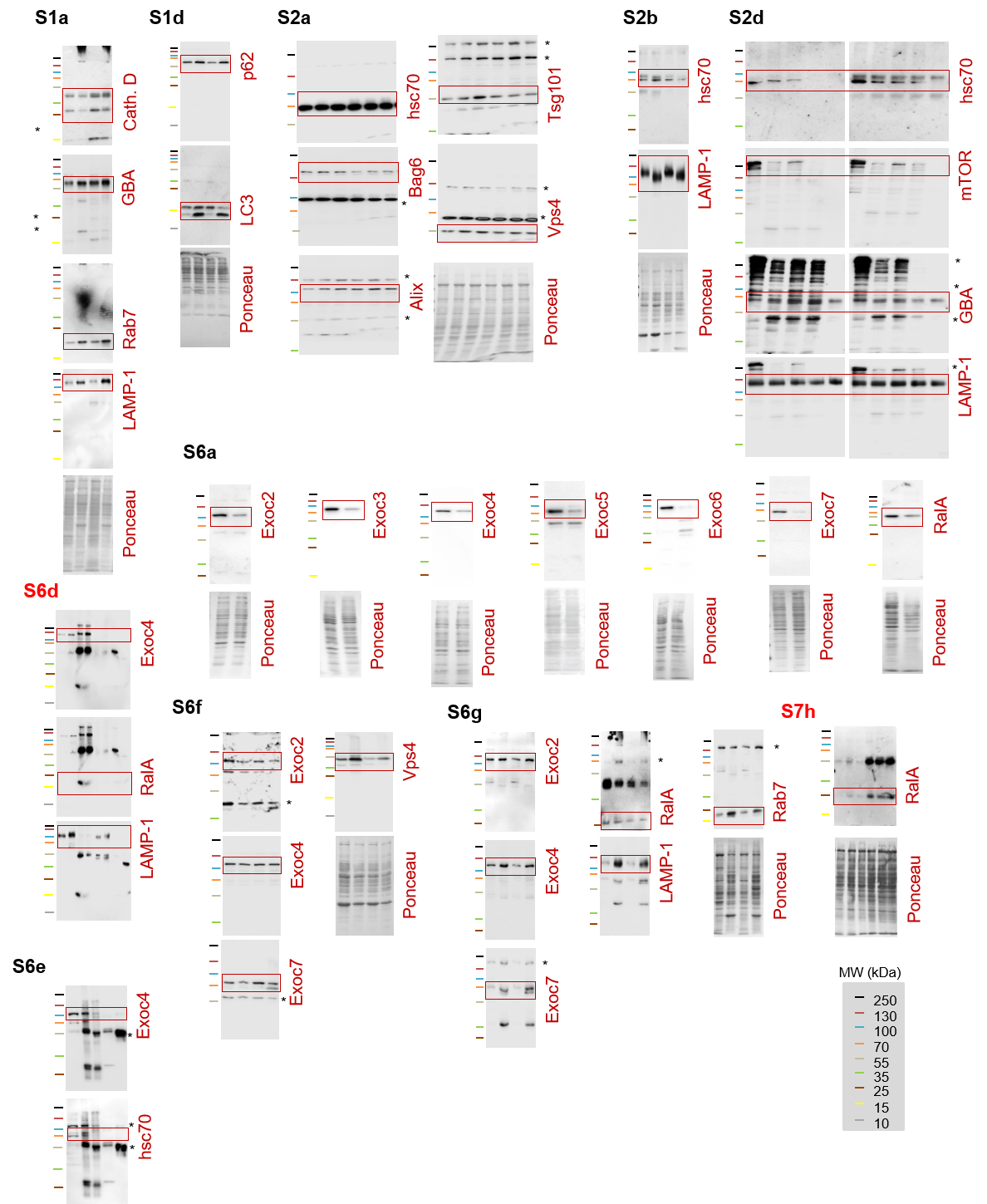
**

**Supplementary Data 3. Uncropped gels from Supplementary Figures 1-5**. Dotted red boxes mark cropped areas shown in the main figure of the membrane blotted for the protein indicated on the right. Ponceau staining is shown in those instances in which total protein per line was used for normalization in the densitometric analysis.
